# Small molecules that disrupt RAD54-BLM interaction hamper tumor proliferation in colon cancer chemoresistance models

**DOI:** 10.1172/JCI161941

**Published:** 2024-02-29

**Authors:** Ekjot Kaur, Ritu Agrawal, Rimpy Arun, Vinoth Madhavan, Vivek Srivastava, Dilip Kumar, Pragyan Parimita Rath, Nitin Kumar, Sreekanth Vedagopuram, Nishant Pandey, Swati Priya, Patrick Legembre, Samudrala Gourinath, Avinash Bajaj, Sagar Sengupta

**Affiliations:** 1 Biotechnology Research Innovation Council—National Institute of Immunology (BRIC-NII), New Delhi, India.; 2 Singapore Immunology Network, Agency for Science, Technology and Research (A*STAR), Singapore.; 3 School of Life Sciences, Jawaharlal Nehru University, New Delhi, India.; 4 Regional Centre for Biotechnology, NCR Biotech Science Cluster, Faridabad, Haryana, India.; 5 UMR CNRS 7276, INSERM U1262, CRIBL, Université Limoges, Limoges, France.; 6 Biotechnology Research Innovation Council—National Institute of Biomedical Genomics (BRIC-NIBMG), Kalyani, India.

**Keywords:** Oncology, Colorectal cancer, DNA repair

## Abstract

RAD54 and BLM helicase play pivotal roles during homologous recombination repair (HRR) to ensure genome maintenance. BLM amino acids (aa 181–212) interact with RAD54 and enhance its chromatin remodeling activity. Functionally, this interaction heightens HRR, leading to a decrease in residual DNA damage in colon cancer cells. This contributes to chemoresistance in colon cancer cells against cisplatin, camptothecin, and oxaliplatin, eventually promoting tumorigenesis in preclinical colon cancer mouse models. ChIP-Seq analysis and validation revealed increased BLM and RAD54 corecruitment on the MRP2 promoter in camptothecin-resistant colon cancer cells, leading to BLM-dependent enhancement of RAD54-mediated chromatin remodeling. We screened the Prestwick small-molecule library, with the intent to revert camptothecin- and oxaliplatin-induced chemoresistance by disrupting the RAD54-BLM interaction. Three FDA/European Medicines Agency–approved candidates were identified that could disrupt this interaction. These drugs bound to RAD54, altered its conformation, and abrogated RAD54-BLM–dependent chromatin remodeling on G5E4 and MRP2 arrays. Notably, the small molecules also reduced HRR efficiency in resistant lines, diminished anchorage-independent growth, and hampered the proliferation of tumors generated using camptothecin- and oxaliplatin-resistant colon cancer cells in both xenograft and syngeneic mouse models in BLM-dependent manner. Therefore, the 3 identified small molecules can serve as possible viable candidates for adjunct therapy in colon cancer treatment.

## Abstract 2

Three FDA/EMA-approved small molecules revert chemoresistance in colon cancer by disrupting RAD54-BLM mediated chromatin remodeling.

## Graphical abstract

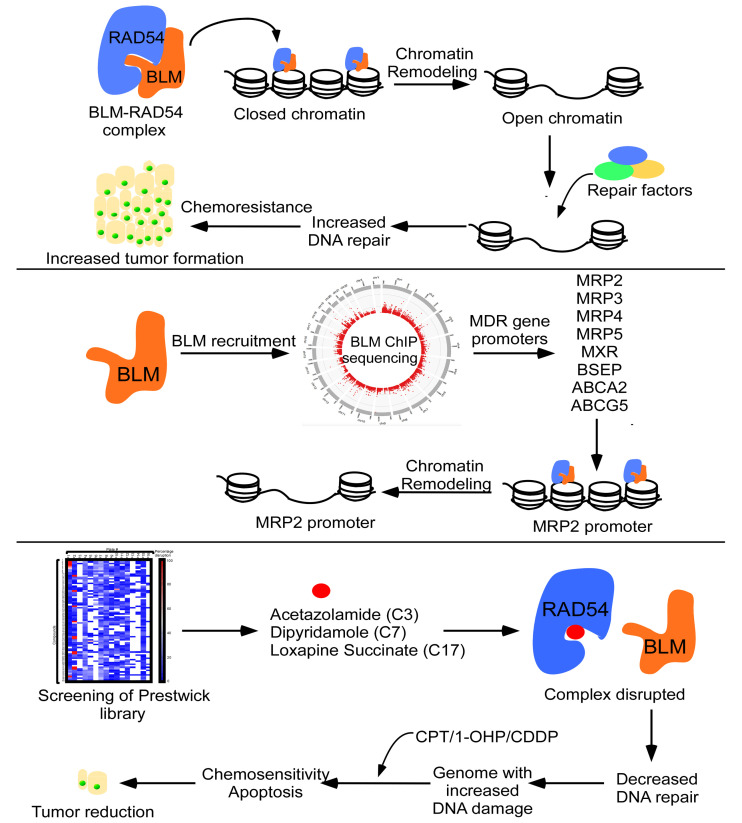

## Introduction

Cancer therapy usually involves utilization of DNA-damaging agents that eliminate cancer cells more efficiently than the normal tissue cells. However, the efficacy of these toxic agents can be modulated by the sensing and subsequent repair of the damaged DNA (1). Dysregulation of one or more DNA repair pathways has been associated with tumor initiation and progression. In addition, heightened DNA repair capacity in the cancer cells has been implicated in therapy resistance and thus poses a major challenge in the management of cancer (2). In particular, homologous recombination repair (HRR) has been implicated in cancer development and drug resistance (3).

BLM is a 1,417–amino acid–containing multifunctional protein that functions both during DNA damage sensing and DNA repair (4). During DNA damage sensing, BLM functionally interacts with multiple key proteins during DNA damage response (5). Furthermore, during the repair phase, BLM functions throughout HRR using several different mechanisms (4). Lack of functional BLM protein has been associated with a rare genetic disorder called Bloom syndrome (BS) (6). Typical characteristics of patients with BS include increased sensitivity toward DNA-damaging agents, which include hydroxyurea (HU), camptothecin, and ionizing radiation, thereby predisposing these patients to a wide spectrum of cancers, including solid cancers, leukemias, and lymphomas (7). Recent reports have suggested that, in multiple cancers, including colon cancer, BLM protein is aberrantly overexpressed, and this occurrence has been linked to poor patient outcome (4). Another core factor in the HRR pathway, RAD54, is involved in multiple crucial steps in this repair pathway in concert with the central homologous pairing protein, RAD51 (8).

Remodeling of chromatin occurs at different steps during DNA damage response. RAD54 has been demonstrated to function as a chromatin remodeler, both in vitro (9) and in cells (10). The remodeling complexes also play a critical role in repositioning of the nucleosomes immediately after exposure to DNA damage in order to provide repair enzymes access to the damaged DNA, thereby ensuring that the response pathway becomes operative and thus preventing genomic alterations (11, 12). The N-terminal (aa 1–212) region of BLM enhanced the chromatin remodeling activity of RAD54 (13).

We now demonstrate that an internal stretch of 32 amino acids in the N-terminal region of BLM was sufficient to stimulate RAD54-mediated chromatin remodeling. This led to decreased levels of damaged DNA, which culminated in enhanced chemoresistance to camptothecin, cisplatin, and oxaliplatin in cellulo and in vivo. We established that the multidrug resistance–associated protein 2 (MRP2, also known as ABCC2) was targeted by RAD54-BLM–mediated chromatin remodeling, leading to its enhanced transcription. Screening of the Prestwick small-molecule library led to the identification and subsequent validation of 3 candidates that could disrupt the RAD54-BLM interaction. These molecules bound to RAD54, abrogated BLM-mediated enhancement of the chromatin remodeling activity, and reduced HRR efficiency. In mouse models, each of the 3 drugs in combination with camptothecin and oxaliplatin diminished tumor growth in BLM-dependent manner. Therefore, these drugs are viable candidates for colon cancer adjunct therapy in combination with the chemotherapeutic drugs used in clinics.

## Results

### BLM (aa 181–212) enhanced RAD54-dependent chromatin remodeling.

It has been previously demonstrated that the N-terminal region of BLM (aa 1–212) enhanced RAD54-mediated chromatin remodeling (13). To characterize the RAD54-BLM interaction in more detail, we checked the relative levels of the 2 proteins in the whole cell extracts from normal colon epithelial cells (CCD 841 CoN) and a colon cancer cell line (HCT116). In normal cells, the levels of both BLM and RAD54 were very low (Supplemental Figure 1A; supplemental material available online with this article; https://doi.org/10.1172/JCI161941DS1), which possibly resulted in decreased RAD54-BLM interaction, as determined by proximity ligation assay (Supplemental Figure 1, B and C). Like in HCT116 cells, the RAD54-BLM interaction was also observed in murine protein lysates obtained from CT26 cells (Supplemental Figure 1D).

**Figure 1:**
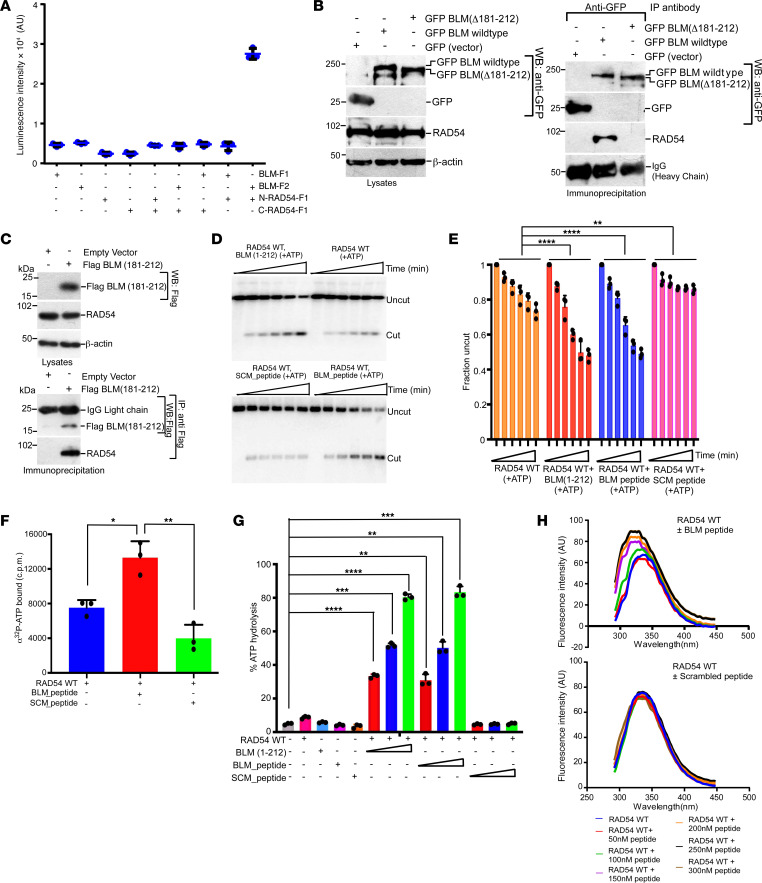
BLM (aa 181–212) enhanced RAD54-mediated chromatin remodeling. (**A**) The N-terminal region of RAD54 interacted with BLM (aa 181–212) in cells. N-RAD54-F1, C-RAD54-F1, BLM-F1, and BLM-F2 were transfected in HEK293T cells. Then, Renilla luciferase–based PCAs were carried out with the indicated combination of expressed proteins. (**B**) Lack of amino acids 181–212 in BLM abrogates its interaction with RAD54 in cells. HCT116 BLM^–/–^ cells were transfected with GFP BLM WT or GFP BLM (aa Δ181–212), and lysates were made. Immunoprecipitations were carried out with anti-GFP antibodies and probed for RAD54. One representative experiment is shown. (**C**) BLM (aa 181–212) interacts with endogenous RAD54 in cells. As in **B**, except HCT116 BLM^–/–^ cells were transfected with p3XFlag-Myc-CMV24 BLM (aa 181–212) or the empty vector. (**D** and **E**) BLM (aa 181–212) enhanced ATP-dependent RAD54-mediated chromatin remodeling. (**D**) REA assays were carried out with chromatinized G5E4 array using indicated experimental conditions in presence of ATP. The reactions were stopped after 0, 2, 4, 6, 8, and 10 minutes. (**E**) Quantitation of **D**. (**F**) BLM (aa 181–212) enhanced the ATP binding capacity of RAD54. Quantitation of the ATP binding assays carried out as indicated. (**G**) BLM (aa 181–212) peptide increased the ATPase activity of RAD54. Quantitation of the ATPase activity carried out as indicated. (**H**) BLM (aa 181–212) peptide altered the conformation of RAD54. Tryptophan fluorescence assays were carried out with RAD54 WT or RAD54 WT in presence of concentrations of BLM_peptide or SCM_peptide. Experiment was repeated 3 times. One representative experiment is shown. (**A** and **E**–**G**) Data are shown as the mean ± SD. Data are from 3 independent experiments. *P < 0.05, **P < 0.01, ***P < 0.001, ****P < 0.0001, (**E**) 2-way ANOVA, (**F**) 1-way ANOVA; (**G**) paired *t* test.

Our earlier results have indicated that a stretch of 32 amino acids in BLM was sufficient for the RAD54-BLM interaction (13). Using a Renilla luciferase–based protein complementation assay (PCA) (14), we determined that BLM (aa 181–212) cloned to luciferase fragment (BLM-F2) was sufficient to interact with the N-terminal region of RAD54 (aa 1–212, N-RAD54-F1) (Figure 1A). Both in vitro interactions with recombinant proteins (Supplemental Figure 1E) and immunoprecipitation in cells (Figure 1B) indicated complete loss of RAD54-BLM interaction when BLM (aa 181–212) was deleted. Furthermore, Flag-tagged NLS BLM (aa 181–212) interacted directly with endogenous RAD54 (Figure 1C). We next wondered whether BLM (aa 181–212) was sufficient to enhance RAD54-mediated chromatin remodeling. Therefore, measurement of the chromatin remodeling activity was carried out by using a restriction enzyme accessibility (REA) assay on chromatinized G5E4 array (15) (Supplemental Figure 1G, top), using recombinant RAD54, BLM (aa 1–1,417), or BLM (aa Δ181–212), BLM (aa 1–212) (Supplemental Figure 1F), BLM peptide (aa 181–212) (termed BLM_peptide), or a scrambled peptide (termed SCM_peptide) having the same amino acid composition but different sequences. We found that BLM (aa 1–1,417), BLM (aa 1–212), and BLM_peptide — but neither BLM (aa Δ181–212) nor SCM_peptide — could enhance the chromatin remodeling activity of RAD54 (Figure 1, D and E, and Supplemental Figure 1, G and H). BLM_peptide carried out this function by enhancing the binding of ATP to RAD54 (Figure 1F), leading to increased ATP hydrolysis (Figure 1G). To further determine how BLM peptide affects ATP binding and hydrolysis, tryptophan fluorescence assays were carried out using full-length recombinant RAD54 in presence of either BLM_peptide or SCM_peptide (Figure 1H). Increasing amounts of BLM_peptide led to progressively enhanced fluorescence quenching, thereby indicating that BLM alters the conformation of RAD54 by interacting via the internal 32 amino acids (aa 181–212).

### Interaction of BLM with RAD54 enhanced cell proliferation.

**Figure 2:**
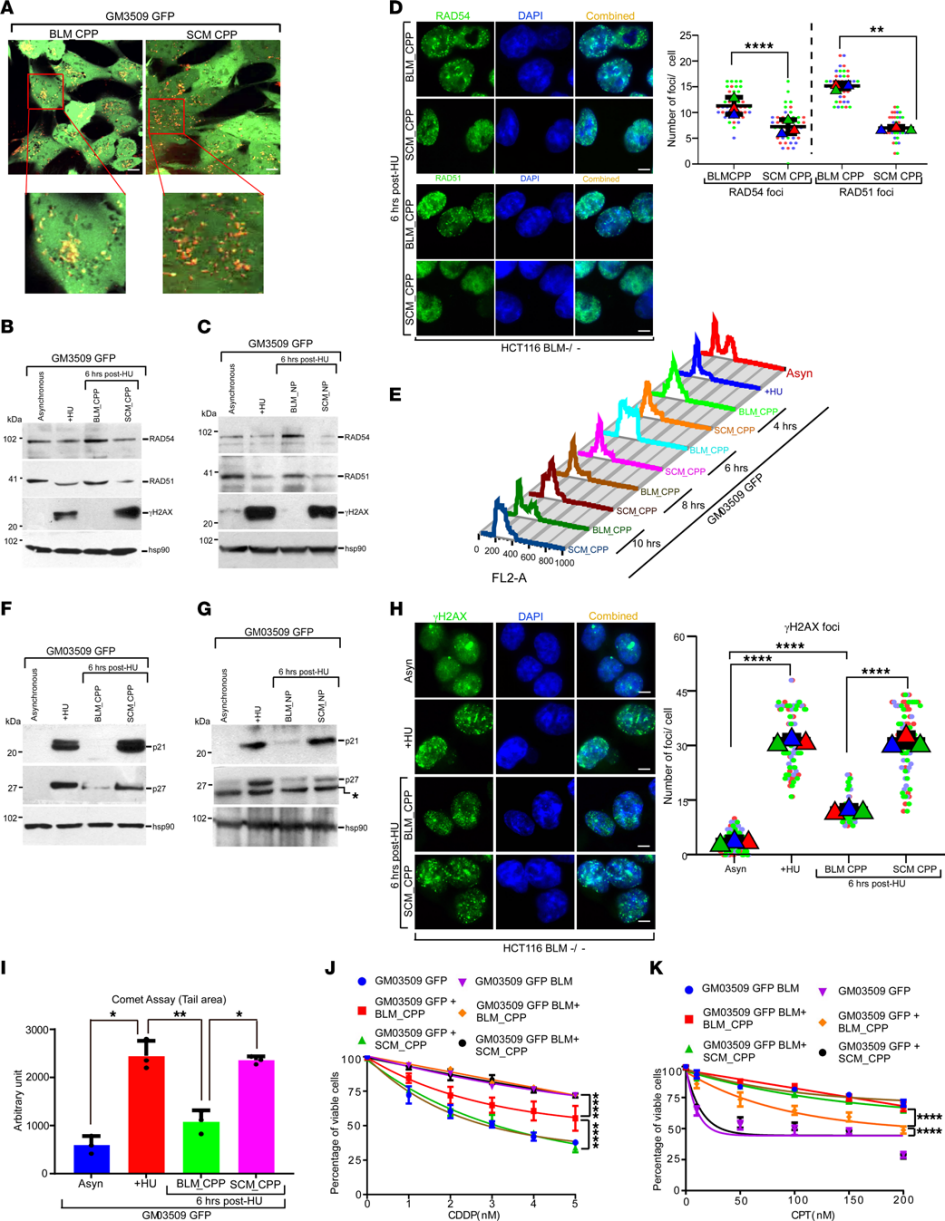
Interaction between BLM (aa 181–212) and RAD54 enhanced chemoresistance in cells. (**A**) Cellular uptake of BLM (aa 181–212) cell-permeable peptide (BLM_CPP) and scrambled cell–permeable peptide (SCM_CPP). The intake of the TAMRA tagged peptides was monitored by live-cell imaging. (**B** and **C**) Levels of RAD51, RAD54, and γH2AX were altered after treatment with BLM_CPP. GM03509 GFP cells were grown in presence of HU for 16 hours or 6 more hours after washing away HU, in presence of (**B**) 180 nM BLM_CPP or SCM_CPP or (**C**) BLM_NP or SCM_NP. Lysates made were probed with indicated antibodies. Experiment was repeated 4 times, and representative blots are presented. (**D**) RAD51 and RAD54 foci numbers were increased after treatment with BLM (aa 181–212) peptide. As in **B** and **C**, except HCT116 BLM^–/–^ cells were fixed and processed for immunofluorescence with indicated antibodies. (**D** and **H**) Experiment was repeated 3 times, and representative images and quantitation (foci/cell) are presented. Number of cells analyzed = 45. (**E**) Presence of BLM_CPP allowed GM03509 GFP cells to proliferate. As in **B** and **C**, except cells released after HU treatment were grown for 4 hours with 180 nM BLM_CPP or SCM_CPP, washed, and allowed to grow for the indicated time intervals. Cells were analyzed by flow cytometry. (**F** and **G**) Presence of BLM_CPP or BLM_NP decreased the levels of the CDK inhibitors. GM03509 GFP-BLM and GM03509 GFP cells released after HU treatment were grown for 6 hours with (**F**) 180 nM BLM_CPP or SCM_CPP or (**G**) BLM_NP or SCM_NP. Lysates made were probed with indicated antibodies. Experiment was repeated 4 times, and representative blots are presented. The asterisk represents a cross-reactive band in **G**. (**H**) γH2AX foci numbers were decreased after treatment with BLM_CPP. As in **B** and **C**, except HCT116 BLM^–/–^ cells were processed for immunofluorescence with γH2AX antibody. (**I**) BLM_CPP decreased the levels of cellular DNA damage. Cells treated with HU (16 hours) were grown for 6 hours with 180 nM BLM_CPP or SCM_CPP, after which Comet assays were carried out. (**J** and **K**) BLM_CPP increased cellular resistance to cisplatin and camptothecin. Cells were treated with 180 nM BLM_CPP or SCM_CPP in presence of (**J**) 1, 2, 3, 4, or 5 nM CDDP or (**K**) 10 nM, 50 nM, 100 nM, 150 nM, 200 nM of CPT. The percentage of viable cells was determined by MTT assays. The data are from (**J**) 4 and (**I** and **K**) 3 independent experiments. Data are shown as the mean ± SD. *P < 0.05, **P < 0.01, ****P < 0.0001, (**I**) 1-way ANOVA; (**D**, **H**, **J**, and **K**) 2-way ANOVA. Scale bar: 5 μM.

We next evaluated whether the RAD54-BLM interaction altered the repair response and thereby influenced cell growth. To study the effect of the RAD54-BLM interaction within the cells, we generated a TAMRA-tagged cell-permeable peptide for BLM (aa 181–212) (termed BLM_CPP) and the scrambled sequence (termed SCM_CPP). Both the peptides were linked to an N-terminal SV40-derived nuclear localization signal (NLS). We tested the peptides in GM03509 GFP cells lacking BLM expression. Using live-cell imaging, we found that both BLM_CPP and SCM_CPP entered the nucleus (Figure 2A). The presence of both BLM_CPP and the nanoparticle coated BLM peptide (BLM_NP) led to enhancement in the endogenous levels of prorecombination proteins RAD51 and RAD54, even after the damage inducer HU had been washed off (Figure 2, B and C). Consequently, the number of RAD51 and RAD54 foci increased upon BLM_CPP exposure, indicating enhanced DNA repair, which is known to be associated with cell cycle progression and tumor growth (Figure 2D).

To determine the consequences of the presence of BLM_CPP within the cells, GM03509 GFP cells were treated with HU, which arrested the cells in the G_1_/S boundary. Subsequently, HU was washed off and the cells were released for different time intervals in the presence of either BLM_CPP or SCM_CPP. Flow cytometry analysis revealed that BLM_CPP allowed the cells to enter into proliferation mode much earlier than the cells treated with SCM_CPP (Figure 2E). Western analysis revealed that the levels of cyclin-dependent kinase inhibitors p21 and p27 were reduced when cells were treated with either BLM_CPP or BLM_NP (Figure 2, F and G), thereby allowing increased proliferation under these conditions. This was accompanied by decreased levels of residual DNA damage in BLM_CPP–treated GM03509 GFP cells, as measured by γH2AX foci and protein levels (Figure 2, B, C, and H) and Comet assays (Figure 2I).

We hypothesized that the proproliferative effect might promote tumor resistance to chemotherapeutic drugs. Isogenic lines GM03509 GFP and GM03509 GFP-BLM were exposed to a gradient of cisplatin (CDDP) or camptothecin (CPT). Compared with GM03509 GFP-BLM cells, GM03509 GFP cells were more sensitive to both the tested drugs. However, GM03509 GFP cells pretreated for 6 hours with BLM_CPP (but not SCM_CPP) displayed resistance to the drugs (Figure 2, J and K). These findings were confirmed using 5 different colon cancer cells (HCT116, DLD1, HT-29, SW480, and SW620) and their respective isogenic controls, in which the expression of BLM was ablated (Supplemental Figure 2, A, E, G, I, and K). Addition of BLM_CPP or BLM_NP consistently enhanced the resistance to CDDP, CPT, or CPT_NP in all the cases (Supplemental Figure 2, B–D, F, H, J, and L).

### BLM enhanced RAD54-mediated chromatin remodeling on MRP2 gene promoter.

**Figure 3:**
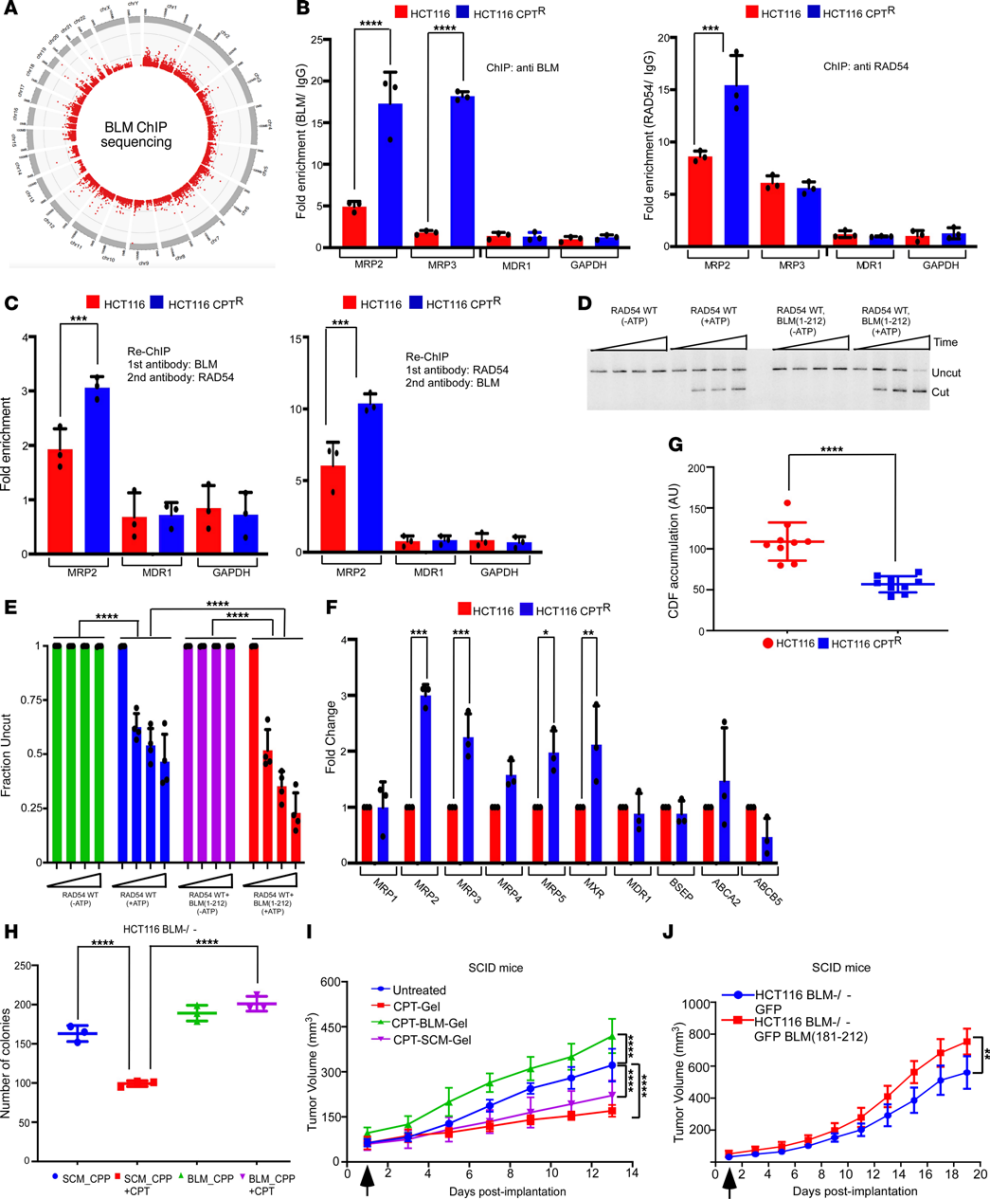
Chromatin remodeling by RAD54-BLM complex on *MRP2* promoter enhances chemoresistance. (**A**) Circos plot obtained from BLM Chip-Seq analysis carried out on GM03509 BLM Clone 9.6 cells. (**B** and **C**) Both BLM and RAD54 were corecruited to *MRP2* promoter. Chromatin isolated from HCT116 WT and HCT116 IC60 CPT^R^ cells was used for (**B**) ChIP or (**C**) Re-ChIP. DNA obtained was used to determine the enrichment on (**B**) *MRP2*, *MRP3*, *MDR1*, and *GAPDH* promoters and (**C**) *MRP2*, *MDR1*, and *GAPDH* promoters by qPCR. Data are from 3 independent experiments. (**D** and **E**) BLM (aa 1–212) enhanced ATP-dependent RAD54-mediated chromatin remodeling. (**D**) REA assays were carried out with chromatinized *MRP2* array. Reactions were stopped after 1, 5, and 10 minutes. (**E**) Quantitation of **D**. Data are from 4 independent experiments. (**F**) Enhanced transcription of *MDR* genes occurred in HCT116 IC60 CPT^R^ cells. RNA isolated from HCT116 WT and HCT116 IC60 CPT^R^ cells was used for RT-qPCR. The levels of MRP1, MRP2, MRP3, MRP4, MRP5, MXR, MDR1, BSEP, ABCA2, and ABCB5 were quantitated from 3 independent experiments. (**G**) HCT116 WT IC60 CPT^R^ cells have enhanced MRP2 efflux activity. HCT116 WT and HCT116 WT IC60 CPT^R^ cells were incubated with MRP2 substrate (CDF) for 30 minutes at 37°C. The accumulation of fluorescent product CDF was determined as a measure of MRP2 activity. The experiment was carried out 9 times. (**H**) BLM_CPP enhanced the anchorage-independent growth of HCT116 BLM^–/–^ cells. Soft agar assay was carried out in HCT116 BLM^–/–^ cells by treating them with 180 nM BLM_CPP or SCM_CPP in absence or presence of CPT (120 nM). The number of soft agar colonies in each condition was counted. Data are from 3 independent experiments. (**I**) Treatment with CPT-BLM-Gel enhanced tumor growth in a xenograft mice model. HCT116 BLM^–/–^ cells were injected into SCID mice (*n* = 7 in each group). On day 1, when the tumors were 50 mm^3^, CPT-Gel was injected at the base of the tumors alone or along with the injection of CPT-BLM-Gel or CPT-SCM-Gel. The volume of the tumors was estimated for the indicated days. (**J**) BLM (aa 181–212) region enhanced tumor growth in xenograft mice model. HCT116 BLM^–/–^ cells stably expressing EGFP or EGFP-BLM (aa 181–212) were injected into SCID mice (*n* = 7 in each group). The volume of the tumors was estimated for the indicated days. Data are shown as the mean ± SD. *P < 0.05, **P < 0.01, ***P < 0.001, ****P < 0.0001, (**B**, **C, E, F, I**) 2-way ANOVA; (**G**) Mann-Whitney test; (**H**) 1-way ANOVA; (**J**) Wilcoxon’s test.

Next, we wanted to determine the mechanistic aspects of RAD54-BLM interaction–induced chemoresistance in colon cancer cells. For this purpose, we generated GM03509 BLM clone 9.6 cells by correcting the mutation in the *BLM* gene (c.1784C>A) in the GM03509 fibroblasts obtained from a patient with BS using CRISPR/Cas9-assisted homology directed repair (Supplemental Figure 3A). The expression of BLM protein in GM03509 BLM clone 9.6 cells was similar to that observed in HCT116 WT cells (Supplemental Figure 3B). BLM protein in the CRISPR/Cas9-corrected cells formed foci upon HU treatment (Supplemental Figure 3C) and decreased the high levels of sister chromatin exchanges seen in GM03509 cells (Supplemental Figure 3, D and E). Using these cells, we performed genome-wide mapping of BLM by ChIP sequencing (ChIP-Seq) to determine where BLM is recruited in absence of any damage. We observed widespread BLM binding on various chromosomal locations, as shown in a circos plot (Figure 3A). We then set out to determine whether BLM is recruited to different promoters within 5 kb of TSS of gene promoters. Among others, enrichment could be specifically seen on *MDR* gene promoters — a set of genes known to contribute to chemoresistance. In fact, BLM was recruited to 8 of the 10 tested *MDR* gene promoters, namely *MRP2*, *MRP3*, *MRP4*, *MRP5*, *MXR*, *BSEP*, *ABCA22*, and *ABCG5* (Supplemental Figure 4A).

To further understand the role of the *MDR* genes with respect to RAD54-BLM interaction–induced chemoresistance, we used HCT116 WT cells and generated a cell line that was resistant to camptothecin (HCT116 IC60 CPT^R^). To determine whether the RAD54-BLM complex was specifically recruited onto the *MDR* gene promoters, we performed ChIP-qPCR experiments using BLM and RAD54 antibodies and the parental and resistant cells. The region used to check BLM and RAD54 recruitment by ChIP-qPCR was selected from the BLM ChIP-Seq data set. Both BLM and RAD54 were highly enriched on the tested *MRP2* promoter (Figure 3B). Sequential ChIP (Re-ChIP) experiments further confirmed that BLM and RAD54 were both corecruited onto the *MRP2* promoter with higher occupancy seen in the resistant cells as compared with the WT cells (Figure 3C). We next created an array for REA using sequences from the *MRP2* promoter (Supplemental Table 1). We found that, in parallel assay conditions, RAD54-mediated chromatin remodeling was equivalent in both the G5E4 and *MRP2* promoter array (Supplemental Figure 4, B and C). More importantly, presence of BLM (aa 1–212) substantially increased RAD54-mediated chromatin remodeling of the chromatinized *MRP2* array (Figure 3, D and E). BLM_peptide (but not SCM_peptide) also enhanced RAD54-mediated remodeling activity on *MRP2* array (Supplemental Figure 4, D and E). The enhanced remodeling by RAD54-BLM complex resulted in increased transcription of multiple *MDR* genes (including *MRP2*) in HCT116 IC60 CPT^R^ cells (Figure 3F). In fact, the resistant cells also displayed higher MRP2 activity as compared with the HCT116 WT cells, as seen by the lower levels of CDF fluorescence (Figure 3G).

### Interaction of BLM with RAD54 enhanced neoplastic transformation.

Next, we wanted to determine whether enhanced chemoresistance due to RAD54-BLM had any effect on neoplastic transformation. We first tested whether the presence of BLM_CPP had an effect on the anchorage-independent growth of HCT116 BLM^–/–^ cells. Indeed BLM_CPP (but not SCM_CPP) enhanced the number of soft agar colonies, even in the presence of CPT (Figure 3H). Furthermore, we tested the proliferative capacity of BLM (aa 181–212) in SCID mice in which tumors were developed by subcutaneously implanting HCT116 BLM^–/–^ cells. The mice bearing 50 mm^3^ tumors were randomized into 4 groups: left untreated; injected at the base of the tumors with CPT entrapped in gel (CPT-Gel); CPT and BLM peptide entrapped gel (CPT-BLM-Gel); or CPT and scrambled peptide entrapped gel (CPT-SCM-Gel). Injection of CPT-Gel impaired the tumor growth. However, the presence of CPT-BLM-Gel (but not CPT-SCM-Gel) enhanced the volume of the tumors (Figure 3I). These results were further validated in another SCID mice–based xenograft model in which tumor formation was monitored by implanting HCT116 BLM^–/–^ cells stably expressing either GFP-BLM (aa 181–212) or GFP alone. Expression of GFP-BLM (aa 181–212) augmented the rate of tumorigenesis (Figure 3J), indicating that the 32–amino acid stretch in BLM that interacted with RAD54 promoted tumor growth, even in the presence of the chemotherapeutic drug CPT.

### FDA-approved small molecules disrupt RAD54-BLM interaction.

**Figure 4:**
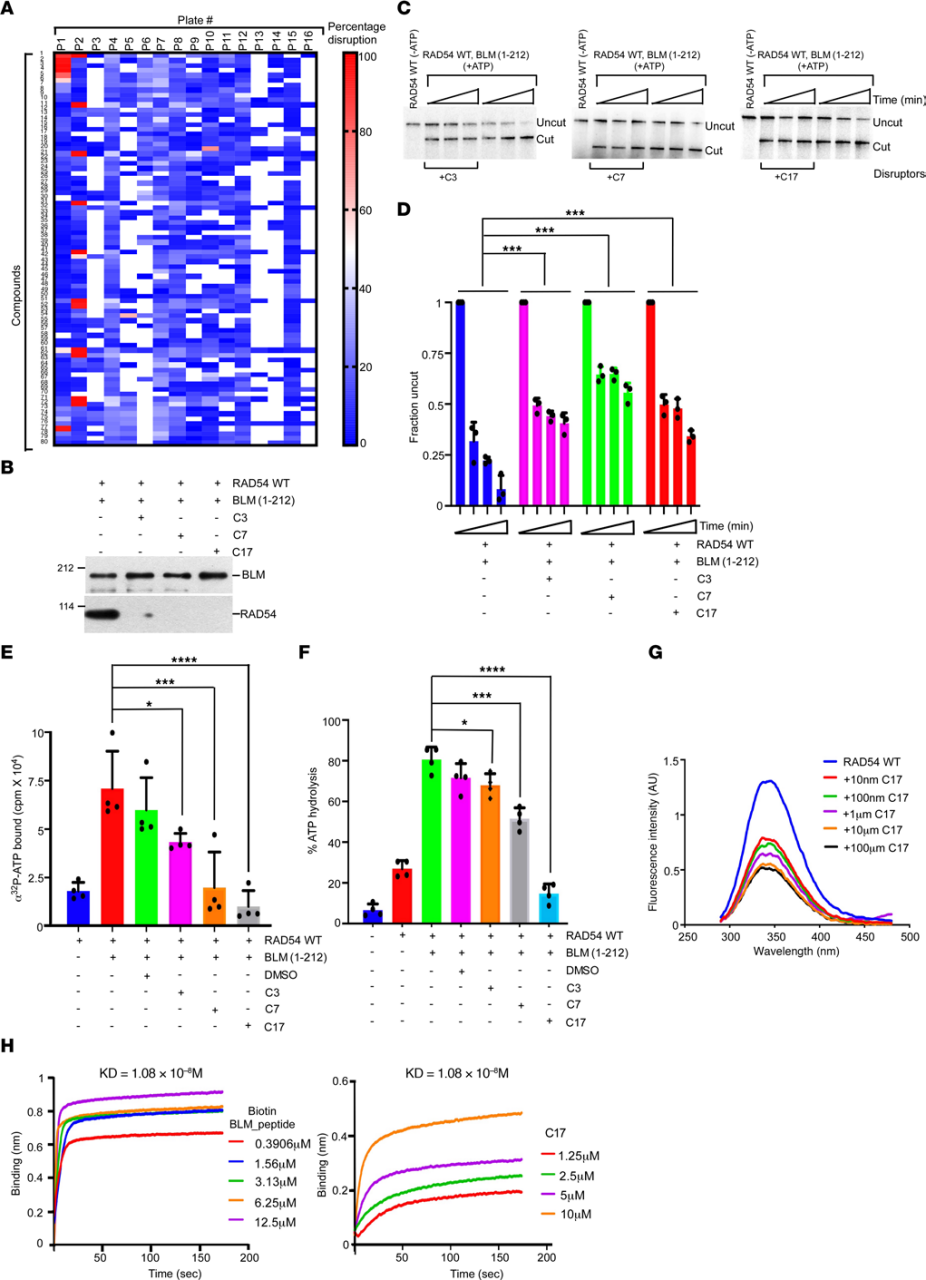
Disruption of RAD54-BLM interaction by small molecules decreased chromatin remodeling. (**A**) Disruption of RAD54-BLM interaction by small molecules was done by screening the Prestwick chemical library using Renilla luciferase–based PCA. Percentage disruption of the interaction between BLM F2 and N-RAD54 F1 was plotted in form of a heatmap. (**B**) C3, C7, and C17 disrupted RAD54-BLM interaction in vitro. In vitro interactions were carried out between bound GST-BLM WT and soluble His-RAD54 WT in the absence or presence of 10 μM C3, C7, or C17. Levels of bound RAD54 were determined by immunoblotting. (**C** and **D**) C3, C7, and C17 decreased the efficiency of BLM-dependent enhancement of RAD54 chromatin remodeling activity. (**C**) REA assays were carried out as indicated using *MRP2* array. The reactions were stopped after 1, 5, and 10 minutes. (**D**) Quantitation of **C**. The data are from 3 independent experiments. (**E**) C3, C7, and C17 decreased BLM-dependent enhancement of the binding of ATP by RAD54. Quantitation of the ATP binding assays was carried out. Data are from 3 independent experiments. (**F**) C3, C7, and C17 decreased BLM-dependent enhancement of the ATPase activity of RAD54. Quantitation of the ATPase activity was carried out. Data are from 4 independent experiments. (**G**) C17 altered the conformation of RAD54. Tryptophan fluorescence assays were carried out with His-RAD54 WT, alone or in presence of the indicated concentrations of C17. RAD54 fluorescence was measured in a fluorometer. Experiment was repeated 3 times, and a representative experiment is shown. (**H**) The affinity of RAD54 to C17 was similar to that of biotinylated BLM (aa 181–212) peptide. Octet BLI-based studies were performed to determine the dissociation constant of the interaction of different concentrations of biotin BLM_peptide and C17 with His-RAD54 WT immobilized onto Ni-NTA-sensor. The affinity constant (KD) ± SD is shown. The experiment was repeated 3 times, and 1 representative experiment is shown. Data are shown as the mean ± SD. *P < 0.05, ***P < 0.001, ****P < 0.0001, (**D**) 2-way ANOVA; (**E** and **F**) 1-way ANOVA.

Having established that RAD54-BLM interaction caused chemoresistance in colon cancer cells, we reasoned that breaking the RAD54-BLM interaction should resensitize colon cancer cells to the chemotherapeutic drugs. Using the Renilla luciferase–based PCA (Figure 1A), we screened 1280 FDA/European Medicines Agency–approved small molecules present in the Prestwick chemical library. The disruption of the RAD54-BLM interaction was determined by a decrease in the Renilla luciferase activity as compared with control untreated cells. The extent of RAD54-BLM disruption by all the tested compounds is shown in the form of heatmap (Figure 4A). Seventeen compounds showed at least 70% disruption of the RAD54-BLM interaction (at 10 μM concentration). Disruption (up to 12%–20%) was observed even when the concentration of the disruptors was 1 nM (Supplemental Figure 5A). Of these, the 3 most potent compounds based on their EC_50_ values (Supplemental Table 2) and the ability to specifically decrease the chemoresistance in the HCT116^–/–^ cell line when challenged with BLM_CPP (data not shown) were acetazolamide (C3), dipyridamole (C7) and loxapine succinate (C17).

Both in vitro (Figure 4B) and cell-based (Supplemental Figure 5B) interaction assays confirmed that these 3 compounds disrupted the RAD54-BLM interaction. Furthermore, attenuation in the BLM-dependent enhancement of RAD54-mediated chromatin remodeling activity on both G5E4 array (Supplemental Figure 4, C and D) and *MRP2* promoter array (Figure 4, C and D) was observed in presence of C3, C7, or C17. The presence of the disruptors also led to a decrease in the ATP binding to RAD54 (Figure 4E) and thereby reduced the extent of ATP hydrolysis (Figure 4F) — both effects probably contributing to the decrease in chromatin remodeling activity by these 3 small molecules (as seen in Figure 4, C and D, and Supplemental Figure 5, C and D).

We further investigated the mechanism by which C3, C7, and C17 affected the RAD54-BLM interaction. We observed that C3, C7, and C17 quenched the tryptophan fluorescence of RAD54, even at a concentration of 10 nM (Figure 4G and Supplemental Figure 5E). This suggested that these small molecules could disrupt the RAD54-BLM interaction by binding and altering the conformation of RAD54.

To quantitate the plausible interaction between C3, C7, and C17 and RAD54 protein, we performed binding kinetics using biolayer interferometry (BLI). Increasing biotinylated BLM (aa 181–212) peptide concentrations led to a sharp association curve with the bound His-RAD54. Moreover, this interaction quickly stabilized and plateaued for all the concentrations. This confirmed a stable interaction between RAD54 and BLM (aa 181–212), with an affinity constant of 1.08 × 10^–8^ M (Figure 4H). Similar experiments were also performed to check the affinity for the compounds C3, C7, and C17 toward bound His-RAD54. All 3 small molecules were able to bind RAD54 with varying affinities. C17 bound with the maximum affinity of 8.03 × 10^–8^ M, which was comparable to that of the BLM peptide (KD = 1.08 × 10^–8^ M) (Figure 4H). C7 and C3 had affinity constants of 5.04 × 10^–6^ M and 5.58 × 10^–5^ M, respectively (Supplemental Figure 5F). The association curves for C17 represent an initial sharper binding followed by slow stabilization (Figure 4H). In the case of C7, we observed a complete saturation at its maximum concentration used, while for C3 a gradual binding kinetics can be observed (Supplemental Figure 5F). Hence, BLI analysis revealed the following order of affinity of the 3 drugs for RAD54: C17 > C7 > C3.

### RAD54-BLM disruptors reverted chemoresistance.

**Figure 5:**
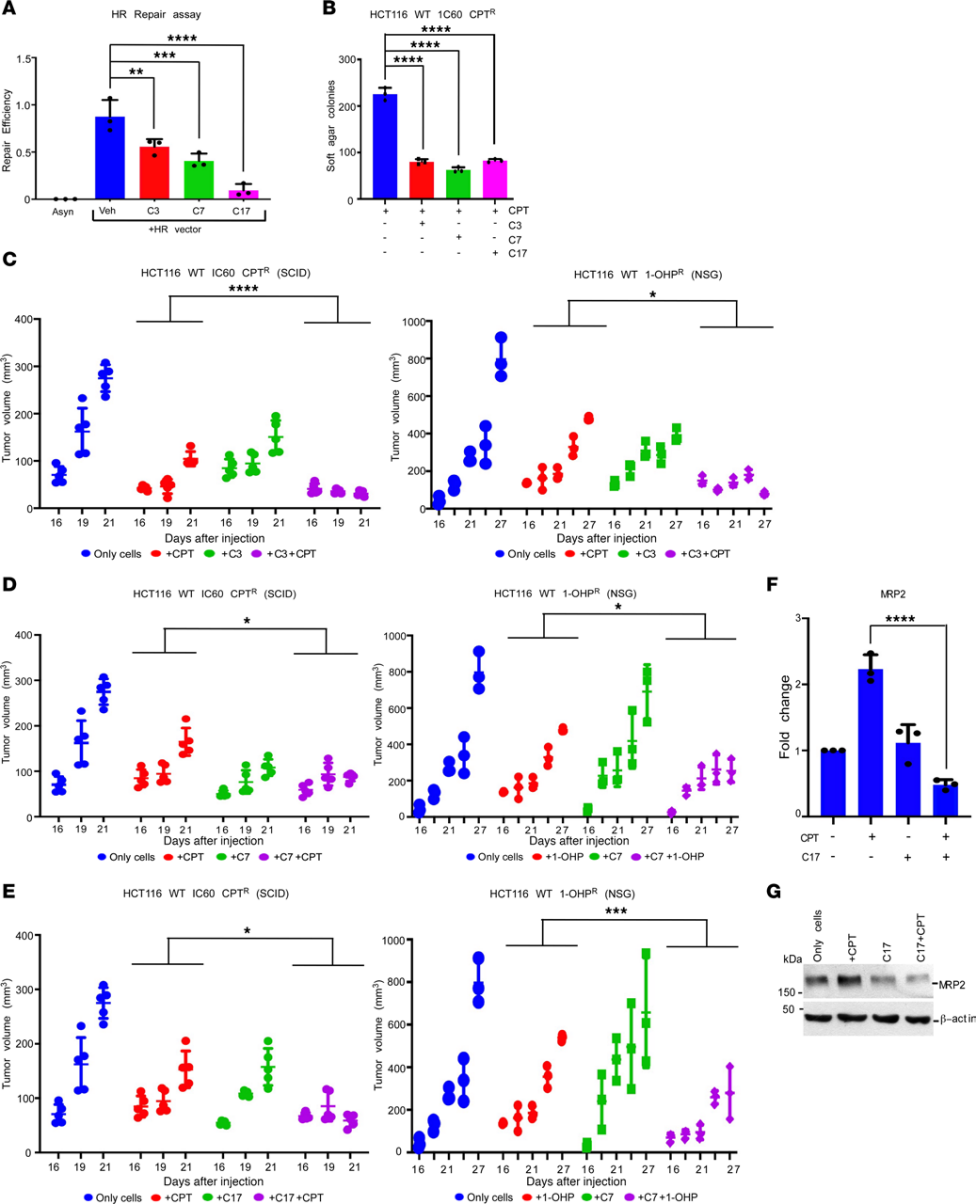
C3, C7, and C17 enhanced the effect of CPT and 1-OHP–mediated decrease in tumor volume in preclinical mice model. (**A**) C3, C7, and C17 decreased the levels of the HRR in HCT116 IC60 CPT^R^ cells. HCT116 IC60 CPT^R^ cells were transfected with the HR substrate for 72 hours, and the levels of HRR were determined in absence or presence of 100 nM C3, C7, and C17. Data are from 3 independent experiments. (**B**) C3, C7, and C17 decreased anchorage-independent cell growth of HCT116 IC60 CPT^R^ cells. Soft agar assay was carried out in HCT116 IC60 CPT^R^ cells by treating them with 100 nM C3, C7, and C17 along with 120 nM CPT. The number of soft agar colonies in each condition was counted. Data are from 3 independent experiments. (**C**–**E**) C3, C7, and C17 decreased tumor formation by camptothecin- and oxaliplatin-resistant cells in 2 xenograft models. HCT116 IC60 CPT^R^ or HCT116 1-OHP^R^ cells were injected into SCID mice (*n* = 5 in each group) or NSG mice (*n* = 3 in each group). The groups were made as indicated. The volume of the tumors was estimated for the indicated days. Data are shown as the mean ± SD. Data for C3 are shown in **C**, C7 in **D**, and C17 in **E**. (**F** and **G**) Treatment with both CPT and C17 decreased MRP2 transcript and protein levels. (**F**) RNA and (**G**) protein was isolated from tumors obtained at the end point of the xenograft experiment. RNA and the protein levels of MRP2 were determined by (**F**) RT-qPCR and (**G**) Western blotting with anti-MRP2 antibody. For each group, 3 tumor samples were analyzed. Data for **F** are from 3 mice. Data for **G** are from 1 mouse and are representative of the 3 mice analyzed. Data are shown as the mean ± SD. *P < 0.05, **P < 0.01, ***P < 0.001, ****P < 0.0001, (**A**, **B**, and **F**) 1-way ANOVA; (**C**–**E**) 2-way ANOVA.

To understand the biological consequences of RAD54-BLM disruptions, we first examined the effect of C3, C7, and C17 on the viability of 3 resistant lines, namely HCT116 IC60 CPT^R^, HCT116 IC60 CDDP^R^ (lines resistant to CPT and CDDP, created for this study), and HCT116 1-OHP^R^ (16). The 3 resistant lines and their WT counterpart HCT116 were exposed to a gradient of CPT, CDDP, or 1-OHP. Treatment with all molecules led to a reduction in the resistance of HCT116 IC60 CPT^R^, HCT116 IC60 CDDP^R^, and HCT116 IC60 1-OHP^R^ to the chemotherapeutic drugs (Supplemental Figure 6, A–C), with a corresponding decrease in the EC_50_ values (Supplemental Tables 3 and 4). In HCT116 IC60 CPT^R^ cells, the extent of the RAD54-BLM interaction was found to be in fact lower compared with HCT116 — thereby indicating that the complex is probably more stabilized in the resistant cells, which leads to phenotype (Supplemental Figure 6D). However, these compounds themselves did not cause any statistically significant change in the expression levels of *MDR* genes (Supplemental Figure 6E). Treatment with the 3 drugs led to decrease in the rate of HRR (Figure 5A). Together with the effect on cell viability, these compounds diminish anchorage-independent growth of camptothecin-treated HCT116 IC60 CPT^R^ cells (Figure 5B).

We also wanted to understand whether the reverted chemoresistance due to C3, C7, and C17 was via modulation of MRP2 activity. As expected, HCT116 IC60 CPT^R^ cells displayed higher MRP2 activity as compared with HCT116 WT cells. Even upon treatment with only CPT or only small molecules the same effect was observed. However, the combinatorial treatment of C3, C7, and C17 with CPT led to enhanced accumulation of CDF dye, suggesting a decreased MRP2 activity in presence of the 3 compounds (Supplemental Figure 6F).

Finally, we evaluated whether C3, C7, and C17 reverted chemoresistance and thereby allowed better efficacy of CPT and 1-OHP in mouse xenograft models by using different resistant lines created in either HCT116 (named as HCT116 IC60 CPT^R^ or HCT116 1-OHP^R^) or HT-29 (named as HT-29 1-OHP^R^) cells. Tumors were generated in either SCID or NSG mice using these cells implanted subcutaneously and injected with either CPT or 1-OHP alone or in combination with C3, C7, and C17. As compared with the mice treated with CPT or 1-OHP alone, the dual treatment of either of the drugs with C3, C7, and C17 inhibited the tumor growth of HCT116 IC60 CPT^R^, HCT116 1-OHP^R^, or HT-29 1-OHP^R^ cells (Figure 5, C–E, and Supplemental Figure 7, A–C). Tumors from different groups were excised at the end of the experiments. Both RNA and protein levels of multiple *MDR* genes (including *MRP2*) were decreased in tumors that had been cotreated with CPT and C17 (Figure 5, F and G, and Supplemental Figure 7, D–G). These results were recapitulated in a syngeneic model using the murine CT26 cells. For this purpose, CT26 CPT^R^ cells were created and injected into nonimmunocompromised BALB/c mice. Like in the xenograft model, dual treatment of CPT with C3, C7, and C17 led to decreased tumor growth (Supplemental Figure 8, A–C), which possibly occurred due to decreased expression of the *MDR* genes at both RNA and protein levels (Supplemental Figure 8, D and E). Further decreased cell proliferation, as seen by decreased Ki67 and PCNA levels (Supplemental Figure 9, A–C) and increased apoptosis, as observed by the enhanced TUNEL positivity (Supplemental Figure 9, D and E), was observed in tumors that had received the dual treatment (CPT and C3, C7, and C17), thus demonstrating the importance of disruption of RAD54-BLM interaction in enhancing the therapeutic response to frontline chemotherapeutic drugs used for the treatment of colon cancer.

**Figure 6:**
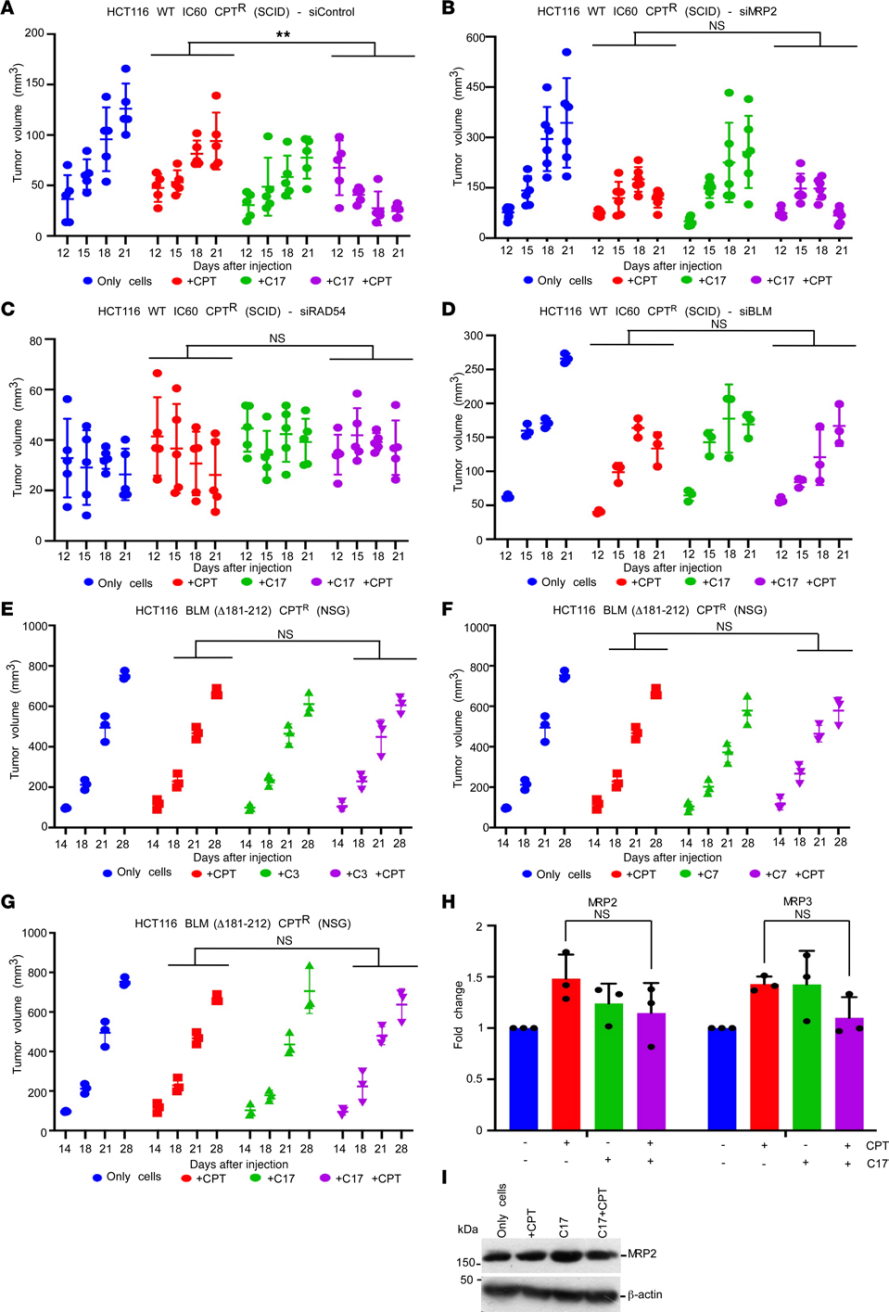
Lack of BLM (aa 181–212) rescues decrease in tumor volume due to CPT and C17 treatment. (**A**–**D**) Ablation of MRP2, RAD54, and BLM in xenograft mice models. HCT116 IC60 CPT^R^ cells were injected into SCID mice to form tumors. The groups made are indicated. When tumors were approximately 50 mm^3^, they were delivered with (**A**) siControl (*n* = 5 mice in each group), (**B**) siMRP2 (*n* = 6 mice in each group), (**C**) siRAD54 (*n* = 5 mice in each group), and (**D**) siBLM (*n* = 3 mice in each group) via TAC6 polymer–mediated in vivo delivery. The volume of the tumors was estimated for the indicated days. (**E**–**G**) Lack of BLM (aa 181–212) residues abrogates C3-, C7-, and C17-mediated decreased tumor formation by camptothecin-resistant cells in xenograft model. HCT116 (aa Δ181–212) CPT^R^ cells were injected into NSG mice (*n* = 3 in each group). The groups were made as indicated. The volume of the tumors was estimated for the indicated days. Data for C3 are shown in **E**, C7 in **F**, and C17 in **G**. (**H** and **I**) Lack of BLM (aa 181–212) residues prevents the decreased MRP2 transcript and protein levels due to treatment with both CPT and C17. (**H**) RNA and (**I**) protein were isolated from tumors obtained at the end point of the xenograft experiment. RNA and the protein levels of the indicated *MDR* genes were determined by (**H**) RT-qPCR for MRP2 and MRP3 and (**I**) Western blotting with anti-MRP2 antibody. For each group, 3 tumor samples were analyzed. Data for **H** are from 3 mice. Data for **I** are from 1 mouse and are representative of the 3 mice analyzed. Data are shown as the mean ± SD. *P < 0.05, **P < 0.01, ***P < 0.001, ****P < 0.0001, (**A**–**F** and **H**) 2-way ANOVA.

To understand whether the 3 key players (MRP2, BLM, and RAD54) were indispensable for reverting chemoresistance in colon cancer, we carried out siRNA-based ablation in xenograft studies using SCID mice. Once the tumor volume reached 50 mm^3^, siControl, siRAD54, siBLM, or siMRP2 were injected at the base of the tumor. The experiment was stopped after 21 days, after which the levels of RAD54, BLM, and MRP2 transcripts were analyzed by RT-qPCR to validate the downregulation of the cognate genes (Supplemental Figure 10). As expected, compared with the use of only CPT, usage of both C17 and CPT in mice injected with siControl led to decreased tumor volume (Figure 6A). Lack of MRP2 led to decreased tumor development upon C17 and CPT treatment. This is probably because of greater retention of CPT inside the tumors, which thereby allowed increased chemotherapeutic potential of the drug (Figure 6B). Loss of RAD54 did not lead to any tumor growth under any of the 4 conditions (Figure 6C), probably because of the role of RAD54 during proliferation and maintenance of genome stability (17). Importantly, ablation of BLM rescued tumor growth even in presence of both C17 and CPT (Figure 6D). Furthermore, a resistant line, HCT116 (aa Δ181–212) CPT^R^ (lacking the 32 amino acids essential for RAD54-BLM in interaction), was created. A xenograft model using HCT116 BLM (aa Δ181–212) CPT^R^ did not show any decrease in the tumor growth due to the dual treatment of CPT with C3, C7, and C17 (Figure 6, E–G). The levels of the tested *MDR* genes at both RNA and protein levels also remain unchanged in the tumors obtained in the mice treated with CPT and C17 (Figure 6, H and I). This established that the 32–amino acid stretch in BLM (i.e., amino acids 181–212) was mandatory to protect cancer cells from the chemotherapeutic drugs. These results together indicated that chemoresistance to camptothecin or oxaliplatin was primarily mediated by BLM (and via its effect on RAD54-mediated chromatin remodeling).

## Discussion

Earlier work has demonstrated that physical interaction between two key HRR factors, BLM helicase and RAD54, enhanced the activity of RAD54 as a chromatin remodeler (13). However, the context in which this interaction had implications on biological or pathological processes had been yet unknown. In this study we extend the earlier observation and demonstrate that enhanced RAD54-BLM functional interaction resulted in increased ability to repair the DNA lesion, thereby decreasing the load of residual cellular damage, which ultimately resulted in enhanced proliferation culminating in chemoresistance in colon cancer cells toward multiple genotoxic agents, as seen in both cellular and preclinical models.

The results of our genome-wide ChIP-Seq study suggest that the extent of RAD54-BLM–mediated chromatin remodeling is one of the key factors that causes chemoresistance to drugs used in colon cancer therapy. One of the targets of the chromatin remodeling by RAD54-BLM is the *MRP2* promoter. MRP2 is a unidirectional efflux transporter that has been implicated in the removal of multiple anticancer agents, such as cisplatin (18), camptothecin (19), and oxaliplatin (20). Furthermore, the levels of MRP2 are higher in patients with colon cancer and contribute to chemoresistance (21). We demonstrate that the enhanced chromatin remodeling mediated by RAD54-BLM on the *MRP2* promoter in the camptothecin-resistant cells possibly allowed greater efflux of the chemotherapeutic drug, thereby leading to chemoresistance. Until now, not much has been known about how *MRP2* is regulated during drug response. At present, MRP2 is only known to be downregulated posttranscriptionally by miR-297 (22). However, the chromatin remodeler SWI/SNF complex was shown to control the transcription of another efflux pump, ABCB1 (23). Thus, this study links genomic organization and chemoresistance and possibly provides hints on how this linkage can be broken to enhance sensitivity to the chemotherapeutic compounds. While we identify MRP2 to be regulated by the RAD54-BLM interaction, identification of other genes that are coregulated by both BLM and RAD54 will also better understanding of the cancer resistance. The results also suggest that the RAD54-BLM interaction would be one of the key factors responsible for the chemotherapy resistance in colon cancer. This is probably due to the fact that this interaction positively affects chromatin remodeling, which is an upstream enabling event that allows greater accessibility to all the different cellular DNA repair pathways activated upon CPT, CDDP, or 1-OHP treatment.

### Clinical correlation of the study.

Both BLM and RAD54 have been implicated in chemo- and/or radioresistance. For example, RAD54^–/–^ mice have been shown to reduce the resistance to ionizing radiation contributed by the compromised HR repair (24). Reciprocally, the chicken B cell line DT40 lacking functional RAD54 showed increased X-ray sensitivity compared with WT cells (25). Recent evidence has shown that RAD54L regulation contributed to radioresistance and neoplastic transformation in glioblastoma (26, 27) and head and neck cancer (28). Overexpression of BLM and RAD51 facilitates HRR and thereby causes resistance of BCR/ABL-positive leukemia cells to DNA damaging drugs (29). Patients with BS are known to be sensitive to both chemotherapy and radiotherapy (7, 30, 31). Therefore, this study provides a firm mechanistic basis on how two key HRR proteins, BLM and RAD54, cause resistance to the common chemotherapeutic drugs used for the treatment of colon cancer. However, BLM helicase is also recognized as a tumor suppressor protein that is involved in DNA damage sensing and DNA repair processes, predominantly HRR (4). The answer to this apparent paradox probably lies in the levels of BLM and RAD54 before and after they undergo neoplastic transformation. BLM is overexpressed in colon cancer cells and patients with shorter relapse-free survival (32). Our unpublished in silico analysis with The Cancer Genome Atlas Colon Adenocarcinoma data set revealed that both BLM and RAD54 were highly overexpressed in the colon cancer samples and that their expression pattern was strongly correlated. Based on these observations, we disrupted the RAD54-BLM interaction with FDA-approved drugs with the aim to identify compounds that could be repositioned as chemosensitizers for patients with colon cancer.

Screening of FDA-approved compounds using a Renilla luciferase–based PCA led to the discovery of 3 compounds that disrupted RAD54-BLM interaction at a nanomolar concentration range in cells and were effective in lowering the tumor load in preclinical mouse models. RAD54-BLM complex disruption abrogated the chromatin remodeling activity of RAD54, limited the accessibility of the repair proteins to the sites of DNA damage, and enhanced the apoptosis in these resistant cells. It is noteworthy that, though C3, C7, and C17 can disrupt the interaction between BLM and RAD54 at a low nanomolar range, we had carried out the chromatin remodeling assays in presence of 10 μM of the compounds. This was necessitated owing to the differential sensitivity of the assay systems, and, therefore, the stated hierarchy of the compounds (C17 > C7 > C3, as obtained from the BLI experiment) may not be strictly true, especially in vivo. Mechanistically, we discovered that these 3 compounds altered the conformation of RAD54 and effectively inhibited the RAD54-BLM interaction. It is noteworthy that, even though the ability of C3 and C7 to affect the RAD54-BLM interaction was lower than that of C17, all the 3 compounds were capable of overcoming drug resistance in preclinical models and thus potentially act as chemosensitizers during camptothecin-, oxaliplatin-, and cisplatin-based colon cancer treatment. Notably, C3, C7, and C17 were not classified as part of the pan-assay interference (PAINS) category of compounds (33, 34). The known targets of these compounds from the ChEMBL library (https://www.ebi.ac.uk/chembl/) include carbonic anhydrase I, II, VI, and XII (acetazolamide); equilibrative nucleoside transporter 1 and 3′,5′-cyclic phosphodiesterase (dipyridamole); and serotonin 2a and 2c and D2-like dopamine receptor (loxapine succinate).

Most of the chemotherapeutic drugs approved by the FDA (both with regular and accelerated approvals) have a low complete response rate and, therefore, are unlikely to be an effective cure to a broad spectrum of patients with cancer (35). Several reports have suggested that drug repurposing could overcome the hurdles of discovering newer anticancer drugs (36, 37). Drug repurposing to overcome resistance to different types of therapies in colorectal cancer has been attempted (38). Here, for the first time to our knowledge we show that the 3 FDA-approved small molecules could target the chromatin remodeling, disrupting the enhanced DNA repair in colon cancer cells, and thereby serve as potent chemosensitizers. We believe that the combination of the newly identified drugs targeting the RAD54-BLM interaction with conventional chemotherapeutic regimens might represent an attractive therapeutic option and, thereby, serve as adjunct therapy for patients with colon cancer.

## Methods

### Sex as a biological variant.

Both male and female mice were used in this study in equal proportion. Therefore, in this study, sex was not considered sex as a biological variable.

### Animal studies.

To determine whether CPT-BLM-Gel enhanced tumor growth in a xenograft mouse model, 3 × 10^6^ HCT116 BLM^–/–^ cells, mixed with Matrigel, were injected into SCID mice. The day when the approximate volume of the tumor was 50 mm^3^ was considered as day 1. On day 1, treatment with CPT-Gel was initiated at the base of the tumors either alone or along with the injection of either CPT-BLM-Gel or CPT-SCM-Gel. To authenticate the effect of BLM (aa 181–212) on tumor formation, xenograft studies were carried out in SCID mice with 2 stable lines in which either GFP or GFP NLS BLM (aa 181–212) were expressed in HCT116 BLM^–/–^ cells. 3 × 10^6^ cells were mixed with Matrigel (1:1 ratio) and then injected subcutaneously. To determine the effect of C3, C7, and C17 on their ability to diminish tumor formation, the xenograft models were carried out in SCID or NSG mice using HCT116 WT IC60CPT^R^, HCT116 BLM^–/–^ CPT^R^ BLM (aa Δ181–212), HCT116 1-OHP^R^, or HT29 1-OHP^R^ cells. Upon tumor formation (~50 mm^3^), CPT (1.25 mg/kg) alone; C3, C7, and C17 alone (5 mg/kg); or CPT and C3, C7, and C17 in combination were administered intraperitoneally after every 3 days. For experiments with HCT116 1-OHP^R^ and HT29 1-OHP^R^ cells, 1-OHP was administered at 2 mg/kg dose either alone or in combination with C3, C7, and C17 (5 mg/kg). For experiments involving the shutdown of MRP2, BLM, or RAD54 in HCT116 WT IC60CPT^R^ cells, cells were injected for tumor formation in mice. TAC6 polymer–mediated delivery of siControl, siMRP2, siBLM, and siRAD54 was carried out in the mice bearing 50 mm^3^tumors as described earlier (39). The delivery of the siRNAs (200 ng of siRNA per dose) was done every alternate day 4 times until the mice were sacrificed 21 days after initiation of the experiment. A syngeneic model was generated in BALB/c mice using CT26 CPT^R^ cells. Upon tumor formation, (~50 mm^3^), CPT was administered intraperitoneally at a 1.5 mg/kg dose either alone or in combination with C3, C7, and C17 (5 mg/kg) for every alternate day till the mice were sacrificed. In all cases tumor volume were measured at the indicated days after injection using the following formula: tumor volume = ½ (length × width^2^). Details regarding all animals used are in Supplemental Table 7. Sections of tumors at the end point of the experiment were subjected to multiple downstream assays — like for apoptosis and proliferation assays. Total tumor lysates were also subjected to immunoblotting and RNA isolated was used for RT-qPCR.

### Supplemental materials.

The Supplemental Methods include information about antibodies (Supplemental Table 5), recombinants, reagents, cells, small-molecule library, peptides, Renilla luciferase–based PCA, chromatin remodeling and REA assay, RT-qPCR, ChIP-qPCR, Re-ChIP qPCR, ChIP sequencing, generation of sgRNA-Cas9–expressing vectors, virus production, generation of double-cut donor vector, establishment of CRISPR/Cas9-mediated BLM-corrected BS cell line, stable cell line generation in BLM^–/–^ CPT^R^ cells, purification of proteins, ATPase assay, ATP binding assay, in vitro interaction assays, BLI, tryptophan fluorescence assay, MTT assay, alkaline Comet assay, soft agar assay, HRR assay and sister chromatid exchange, preparation of lipid nanoparticles of camptothecin (CPT_NPs) and peptides (BLM_NPs, SCM_NPs), hydrogel preparations of camptothecin and BLM_peptide, MRP2 activity assay, immunofluorescence and TUNEL assay, and proximity ligation assay. Recombinants and cells that were obtained as gifts have been indicated in Supplemental Table 6 and Supplemental Table 7, while the sequences of all primers have been included in Supplemental Table 8.

### Statistics.

Data are presented as the mean ± SD. Details about the number of samples analyzed for each experiment are mentioned in figure legends. *P* values of less than 0.05 were considered significant. Details about the statistical tests used for each experiment are provided in the figure legends and include paired, 2-tailed *t* test, Mann-Whitney test, Wilcoxon’s test, 1-way ANOVA, and 2-way ANOVA. The software used for statistical analysis was Graph Pad Prism.

### Study approval.

All animal studies were carried out at the National Institute of Immunology, which approved animal ethics protocols (IAEC no. 357/14, IAEC no. 398/15, IAEC no. 567/20).

### Data availability.

Next-generation sequencing data have been deposited in ArrayExpress (a MINSEQE-compliant public database) (accession no. E-MTAB-11372). Values for all data points in graphs are reported in the Supporting Data Values file. Additionally all the data (Western blots and quantitation) have been deposited in a public database (Mendeley Data, 10.17632/zb42k8z4kc.1). Any other data can be requested from the corresponding author.

## Author contribution

EK, R Agrawal, R Arun, VS, and SP performed biochemical experiments. EK, R Agrawal, VM, and NP performed cell biology experiments, including generation of resistant lines. EK and DK made the CRISPR/Cas9-corrected cells. PPR, EK, and SG contributed to biophysical experiments. EK and PL contributed in Prestwick library screening. EK, R Agrawal, SV, AB, VM, and NK carried out animal experiments. EK, R Agrawal, R Arun, VS, DK, PPR, R Agrawal, R Arun, SV, PL, SG, AB, and SS analyzed the data. EK, R Agrawal, and R Arun wrote the first draft of the manuscript, based on which SS wrote the manuscript. The assignment of EK, R Agrawal, and R Arun as equal co–first authors is solely based on their relative contributions toward data generation, initiation, and completion of the studies. EK is the first of the three equal first authors as she carried out a major part of the initial experiments, while R Agrawal and R Arun completed the experiments listed in the revision phase. Since R Agrawal carried out the in vivo experiments in the revision phase, she is listed as the second of the three equal first authors.

## Supplementary Material

Supplemental data

Unedited blot and gel images

Supporting data values

## Version 1 02/29/2024

In-Press Preview

## Version 2 04/15/2024

Electronic publication
