# Supplementary material for: Small molecules that disrupt RAD54-BLM interaction hamper tumor proliferation in colon cancer chemoresistance models
Source: J Clin Invest. 2024 Feb 29;134(8):e161941. doi: 10.1172/JCI161941 (PMC11014671; doi:10.1172/JCI161941)
Supplement: Supplemental data [file jci-134-161941-s061.pdf]

# Supplemental Material

# Small molecules that disrupt RAD54-BLM interaction hamper tumor proliferation in colon cancer chemoresistance models

**Ekjot Kaur[1,10], Ritu Agrawal[1,10], Rimpy Arun[1,10], Vinoth Madhavan[1], Vivek Srivastava[1,2], Dilip Kumar[3], Pragyan Parimita Rath[4,5], Nitin Kumar[1], Sreekanth Vedagopuram[6], Nishant Pandey[6], Swati Priya[1], Patrick Legembre[7], Samudrala Gourinath[4], Avinash Bajaj[6], Sagar Sengupta[1,8,9]**

**[1]National Institute of Immunology, Aruna Asaf Ali Marg, New Delhi 110067, India.**
**[2]Present address: Department of Chemistry and Biochemistry, School of Basic Sciences and Research, Sharda University, Greater Noida-201308, UP, India.**
**[3]Singapore Immunology Network (SIgN), A*STAR (Agency for Science, Technology and Research), Singapore, Singapore**
**[4]School of Life Sciences, Jawaharlal Nehru University, New Delhi 110067, India.**
**[5]Present address: International Centre for Genetic Engineering and Biotechnology, Aruna Asaf Ali Marg, New Delhi 110067, India.**
**[6]Regional Centre for Biotechnology, NCR Biotech Science Cluster, Faridabad, Haryana 121001, India.**
**[7]UMR CNRS 7276, INSERM U1262, CRIBL, Université Limoges, Limoges, France.**
**[8] National Institute of Biomedical Genomics, PO: NSS, Kalyani, 741251, India.**

**[9]Corresponding author:**
**Sagar Sengupta,**
**National Institute of Biomedical Genomics,**
**PO: NSS, Kalyani, 741251,**
**India.**
**Phone: 91-33-29772151**
**Email: ssg2@nibmg.ac.in**

**[10]Equal contribution**

# Supplemental Methods

## Antibodies, recombinants, reagents

All antibodies used are listed in Table S5. All recombinants used have been described in Table S6. All reagents (including chemicals, recombinant proteins, cell lines, peptides, oligonucleotides, animals, deposited data and other materials) used are described in Table S7. All primers are described in Table S8. For ablating proteins in cells 200pmoles of the gene specific and control siRNAs were used. For Protein Complementation Assay (PCA), N-RAD54-F1, C-RAD54-F1, BLM-F1 and BLM-F2 were generated by cloning the humanized Rluc gene and RAD54/BLM fragments into pcDNA3.1 [strategy followed was as described in (1)]. RAD54/BLM PCR fragments were cloned in EcoR1 and ClaI sites of pcDNA3.1. To obtain pET28b-RAD54 (1-747), full-length RAD54 was amplified and cloned into the NotI site of pET28b and orientation confirmed. pEGFP-C1 NLS BLM (181-212) was obtained by cloning the SV40 Large T antigen Nuclear localization Signal in frame with BLM (181-212) sequence into the EcoR1 and BamH1 sites of pEGFP-C1. p3XFlag-Myc-CMV24 NLS BLM (181-212) was obtained by cloning the SV40 Large T antigen Nuclear localization Signal in frame with BLM (181-212) sequence into the NotI and Kpn1 sites of p3XFlag-Myc-CMV24. pLVX AcGFP N1 BLM (Δ181-212) was obtained by sequentially cloning (a) BLM (1-180) into pLVX AcGFP N1 vector at XhoI/EcoR1 sites using BLM (1-1417) EcoR1/BmaH1 mutant as the template, (b) cloning BLM (213-1417) into pLVX AcGFP N1 BLM (1−180) using EcoR1/BamH1 sites. pGEX4T-1 BLM (Δ181-212) was obtained by sequentially cloning (a) BLM (1-180) into pGEX4T-1 vector at BamH1/EcoR1 sites using BLM (1-1417) EcoR1/BmaH1 mutant as the template, (b) cloning BLM (213-1417) into pGEX4T-1 BLM (1−180) using the EcoR1/NotI sites. pUC57-MRP2 was generated by gene synthesis using

sequences from the MRP2 promoter containing 12 nucleosomes with a centrally located HhaI site occluded by one nucleosome (see Table S1 for the MRP2 array sequence).

**Cells**

All cell lines used are described in Table S7. All HCT116 derived cells were grown in McCoy's 5A media, HT-29, DLD1, HEK293T, GM03509 GFP-BLM, GM03509 GFP were grown in DMEM while SW480, SW620 were grown in L15 medium. Cells were grown in 10% Fetal bovine serum supplemented with glutamine and anti-mycotic and anti-bacterial antibiotics. To generate stable lines EGFP-C1 or pEGFP-C1 NLS BLM (181-212) were transiently transfected in HCT116 BLM-/- cells using Lipofectamine 2000. Forty-eight hours post-transfection, cells were selected with G418 (1mg/ml) for 5 days after which the mass obtained was maintained in presence of 200μg/ml G418. All cells were tested to be free from mycoplasma contamination.

To generate camptothecin and cisplatin resistant cells (named as HCT116 IC60 $CPT^R$, HCT116 IC60 $CDDP^R$ respectively), HCT116 wild type were seeded in a 10cm dish to a confluency of 80%. The IC20 concentrations of cisplatin (3.38μM) and camptothecin (12.6 nM) was added for 2 hours and 6 hours, respectively. Post treatment, the cells were washed twice with 1X PBS and fresh media was added. After every 48 hours, the media was changed and IC20 concentration of the drugs was added only if the confluency of cells was at least 80%. The IC20 resistant HCT116 cells was established after 4 rounds after checking the increase in the resistant index as determined using MTT. Similarly, IC40 HCT116 (resistant to 15.5μM of cisplatin and 30.8nM of camptothecin) as well as IC60 HCT116 resistant cells (resistant to 35.09μM cisplatin and 92.6nM of camptothecin, respectively) are also generated following the same protocol. Resistant index was calculated using the following formula: Resistant index=

IC60 of the resistant cells/ IC60 of the parental cells. HT-29 1-OHP$^{R}$ cells (2) were resistant to 2µM of oxaliplatin.

CT26 cells were seeded in a 96-well plate at a density of 5000 cells/well. Camptothecin was serially diluted (50% dilution), ranging from a maximum of 10µM to 10nM. After 24h of cell seeding, the cells were treated with different drug dilutions in four technical replicates. Following a 48h incubation, the viability of the treated cells was assessed using the MTT (3-(4,5-dimethylthiazol-2-yl)-2,5-diphenyltetrazolium bromide) assay, and the concentration determined at which 50% of cell viability is inhibited. For generating resistant CT26 cells, cells grown in a T25 flask (50% confluency) were exposed to camptothecin at 10nM, and kept for 2-3 weeks till its proliferation kinetics matches with the wild type cells. Camptothecin concentration was then increased incrementally up to 70nM, to generate the resistant line.

**Small molecule library, peptides**

Each of the small molecules in Prestwick Chemical Library were dissolved in DMSO to make 100mM stocks. For in vitro and assays in cells, subsequent dilutions were made in 1XPBS, while for in vivo studies, the compounds were dissolved in 1XPBS containing 5% DMSO. The sequence encoding BLM (181-212) was: STAQKSKKGKRNFFKAQLYTTNTVKTDLPPPS (designated as BLM_peptide). A control peptide sequence, ALGFTDQKTPKSKRTVNSKQPFKTPSKNLTAY (designated as the SCM_peptide) was designed which had the same amino acid composition and did not bear homology to any known protein in human protein database. The Cell Permeable Peptide (CPP) versions (BLM_CPP and SCM_CPP) were tagged with SV40 Large T antigen nuclear localization signal (PKKKRKVEDPYC) in the N-terminus and TAMRA in the C-terminus. The biotinylated

version of BLM_peptide was named as Biotin BLM_Peptide. The corresponding versions in nanoparticle form were designated as BLM_NP and SCM_NP.

**Renilla Luciferase based Protein Complementation Assay**

Renilla Luciferase (Rlu) based Protein complementation assay was carried out as described in (1). For preliminary screening of the Prestwick Chemical Library (containing 1280 FDA/EMA approved small molecules), HEK293T cells were seeded in the 6-well plates a day prior to the transfection. Next day the cells were transfected by 2µg each of pcDNA3.1 N-RAD54 F1, pcDNA3.1 C-RAD54 F1, pcDNA3.1 BLM F1, pcDNA3.1 BLM F2 plasmids (alone or in different combinations) using Lipofectamine 2000 (according to the manufacturer's protocol). 36 hours post-transfection, approximately $10^5$ cells were seeded in a 96-well plate and incubated at 37ºC for 2 hours with or without 10µM of the 1280 small molecules present in the library. 10µM of Coelenterazine h was added into each well and Rluc activity was monitored for the first 10 seconds in Varioskan Microplate Reader (Thermo Fisher Scientific). The validation screen was performed with 17 small molecules at 100µM, 10µM, 1µM, 100nM, 10nM and 1nM concentrations. The percentage reduction in the protein-protein interaction was then calculated as compared to the control wells (without any small molecule).

**Chromatin remodeling and restriction enzyme accessibility assay**

The p2085S-G5E4 was digested with Asp718 and ClaI to release the 2.5kb insert. pUC57-MRP2 array was digested with EcoR1 and HindIII to release the 2.6kb insert. The released inserts fragments were end labelled with [$\gamma^{32}$P]ATP using T4 Polynucluotide Kinase enzyme. Nucleosomal reconstitution on the purified fragments were carried out using gradient salt dialysis method in presence of HeLa core histones. The radiolabelled reconstituted arrays were checked (in a 1% agarose gel), and subsequently used in restriction enzyme accessibility (REA)

assays. For each reaction, 500nM RAD54 (wildtype or mutants), 500nM BLM protein (wildtype or all the mutants used) or 180nM BLM_peptide or SCM_peptide, radiolabelled array (1nM) was used in 1X REA buffer (20mM HEPES, pH 7.9; 40mM KCl, 4mM $MgCl_2$) in the presence of 0.4 U/µl of HhaI. The concentration of ATP used in the reactions was 2mM. C3, C7, C17 were added in the reaction mixture at a concentration of 10µM. The reactions were incubated at 30°C for different time intervals (as indicated in the figure legends). BLM-RAD54 disruptors were added at final concentration of 10µM. The reactions were stopped by addition of the stop buffer (50mM EDTA, 1.2% SDS), digested with Proteinase K (1 mg/ml) at 37°C for 30 minutes. Proteins were removed by phenol-chloroform extraction, DNA was ethanol precipitated, washed and analyzed on 1% agarose gels. The intensity of uncut products was measured from Phosphoimager scans taken in Typhoon™ laser-scanner platform (Cytiva) and ratio of the cut and the uncut products was quantitated using the inbuilt software.

**RT-qPCR, ChIP-qPCR, Re-ChIP qPCR**

Total RNA was isolated from cells and tissues using TRIZOL reagent containing 1% β-mercaptoethanol. cDNA was generated using Reverse transcriptase core kit according to manufacturer's protocol. RAD54 and BLM ChIP have been carried out according to published procedures (3, 4). The sequential Re-ChIP was performed as described earlier (5). The concentrations of input samples, ChIP DNA, Re-ChIP DNA were determined by Qubit using dsDNA HS assay kit. 1ng of ChIP DNA or Re-ChIP DNA was used for qPCR with primers which amplify *MRP2*, *MRP3* and *MDR1* promoters. Recruitment onto the GAPDH promoter was used as the internal control. All primer sequences are in Table S8. All qPCR reactions were carried out in were carried out in QuantStudio 3 Real-time PCR system.

**ChIP sequencing**

The ChIP DNA libraries were constructed using NEBNext Ultra II DNA Library Prep with Sample Purification Beads according to the manufacturer's instructions. Next generation sequencing of libraries was performed using Illumina HiSeq 2500 rapid run V2 kit for 1x50 bp. The quality of the raw reads was determined using FastQC (http://www.bioinformatics.babraham.ac.uk/projects/fastqc/), followed by removal adaptors using Trimmomatic (6). Bases with high Phred score (more than 30) were aligned to the current human genome (hg38 assembly) using Bowtie 1 (7). Unique reads were then analyzed to determine the BLM enrichment on TSS as described (8). The data was visualized as circos plot using RStudio and IGV tools.

**Generation of sgRNA-Cas9 expressing vectors**

The homozygous transversion from C>A at nucleotide 1784 (exon 7) in the BLM gene in GM03509 BS cell line was corrected using oligomer-based gRNA-CRISPR-Cas9 lentivirus approach. The approach is based on two sets of LR Gateway compatible plasmids; sgRNAs pDonor and a lentiviral expression plasmid created in this study (PHASE-DEST-CAS9-P2A-GFP). To clone the BLM specific sgRNA targeting the region of interest within BLM gene locus, one predicted sgRNA (CACTGGAAGACAGTCTGTCT) near to the site of interest was selected. A set of forward and reverse 25 bases long oligomers representing the selected sgRNA; CACCGCACTGGAAGACAGTCTGTCT and its inverse complementary primer; AAACAGACAGACTGTCTTCCAGTGC were custom synthesized. The forward and reverse oligonucleotides were mixed at equimolar concentrations, phosphorylated, annealed, ligated to BbsI digested pDonor and transformed in TOP10 cells and plated onto the Kanamycin selection plate. Positive clones of BLM specific gRNAs in pDonor vector were identified through Sanger sequencing and were further shuttled efficiently to the DEST containing lentiviral CAS9 expression plasmid (PHASE-DEST-CMV-CAS9-P2A-GFP) using LR-gateway reaction.

**Virus production**

Viral particles were produced by co-transfection of lentiviral expression plasmid (PHASE-gRNA-CMV-CAS9-P2A-GFP) together with packaging plasmids (pMDLg/pRRE, pRSV/REV, pMD2.G/V-SVG) [1 (8µg): 2 (4µg): 3 (2.66µg) plasmid ratio)] in Lenti-X HEK293T cell line using Xfect transfection reagent (1: 0.5 ratio) in a 10 cm plates. Viral supernatants were collected 72h post transfection and filtered using 0.45µm filter followed by concentration using LentiX concentrator and titrated by qPCR (determination of number of transducing or infectious units per ml) on HeLa cells. The titer for lentivirus were $6\times10^7$ TU/ml. The cleavage efficiency of the sgRNA was determined by performing Surveyor assay using Surveyor Mutation Detection Kit as per manufacturer instructions.

**Generation of double cut donor vector**

The double cut HDR donor vector was generated as described (9). To obtain the HDR donor vector the following steps were sequentially performed (a) antisense strand of BLM sgRNA and the flanking PAM sequence was cloned into the XhoI/HindIII sites of mCherry2-C1; (b) sense strand of BLM sgRNA and the flanking PAM sequence was cloned into the KpnI/BamH1 sites of mCherry2-C1; (c) 1200bp region spanning the site of mutation was PCR amplified from HCT116 WT genomic DNA was cloned into pUC18 at HindIII/KpnI sites, followed by mutating the PAM sequence for the sgRNA; (d) subcloning of the 1200 bp wildtype BLM sequence from pUC18 into mCherry2-C1 using HindIII/KpnI enzymes to generate the double cut donor vector.

**Establishment of CRISPR/Cas9 mediated BLM corrected BS cell line**

GM03509 cells were transduced with the lentiviral vector expressing BLM sgRNA and GFP Cas9 at MOI of 0.1 using 10µg/ml of polybrene. After 48 hours of transduction, 10µg of double cut donor vector was transfected using FuGENE® HD Transfection Reagent. The cells positive for mCherry and GFP were flow sorted and single cell was seeded in each well of the 96-well plate. The clones were screened for correction in the mutation by T7 enzyme mismatch cleavage (EMC) assay or surveyor analysis. PCR product of clone GM03509 BLM Clone 9.6 was cloned into TA vector (according to the manufacturer's protocol) and Sanger sequencing was carried out for 20 sub-clones which confirmed the correction in the genome of GM03509 cells. The expression of BLM in GM03509 BLM Clone 9.6 was then examined using the western blot and immunofluorescence assays. The functionality of the expressed BLM in GM03509 BLM Clone 9.6 cells was demonstrated by SCE assays.

**Stable cell line generation in BLM-/- $CPT^R$ cells**

Viral particles were produced by co-transfection of lentiviral expression plasmid (pLVX-AcGFP1-N1- ΔBLM 181-212) together with packaging plasmids (pMD2.G, psPAX2) (in 2:2:1 ratio) were carried out in HEK293T using Lipofectamine 3000 (in 1: 2 ratio). Viral supernatants were collected 72 hours post-transfection and filtered using a 0.45µm filter, followed by concentrating the virus using a LentiX concentrator. Transduction was performed in HCT116 BLM-/- $CPT^R$ cells using polybrene (10µg/ml). Cells were selected with puromycin 1µg/ml for 14 days.

**Purification of proteins**

All GST-tagged and His-tagged proteins were expressed in *E. coli* BL-21 DE3 competent cells at 16°C overnight and subsequently purified according to standard protocols by binding to either Glutathione-S-Sepharose (for GST-tagged proteins) or Nickel-NTA beads (for His

tagged proteins). The bound proteins were eluted out using either 2mM reduced glutathione (for GST-tagged proteins) or 200mM imidazole (for His tagged proteins). The eluted fractions were pooled, dialysed and used for the assays.

**ATPase assay**

Assay for RAD54 ATPase activity was carried out as per the previously published protocol (10). His-RAD54 (120nM) was incubated with the GST-BLM (1-212) or BLM_peptide or SCM_peptide (180nM). Where gradient of proteins was used, the concentrations have been indicated in the figure legends. C3, C7, C17 were added in the reaction mixture, so that the concentration of 10µM was obtained. φX174RF1 DNA (22µM base pairs) was added to initiate the ATP hydrolysis reaction at 30°C for 15 minutes and 20 nCi of [$\gamma^{32}$P]ATP. 1µl of the sample was spotted on the polyethyleneimine-coated TLC plate, resolved in 1M Formic Acid and 0.3M LiCl. The products of ATP hydrolysis were then visualized by Phosphoimager scans taken in Typhoon™ laser-scanner platform (Cytiva) and quantitated using the inbuilt software.

**ATP Binding assay**

A 20µl reaction was set up with 120nM of GST-RAD54 and 180nM of either BLM_peptide or SCM_peptide or BLM (1-212) using 4µl of 5XATP binding assay buffer (100mM Tris-HCl pH 7.5, 50 mM $MgCl_2$, 10 mM $MnCl_2$, 5 mM DTT) and 5 µCi of [$\alpha^{32}$P]dATP. C3, C7, C17 were added in the reaction mixture at a concentration of 10µM. Reactions were incubated at 30°C for 20 minutes and were stopped with 10% TCA and samples were spotted on P81 phosphocellulose paper followed by washing of paper strips with 75mM orthophosphoric acid. A minimum of 8 washes were given in 20 hrs of 1 hr each including the final wash extending for 12 hours. Following this, strips were dehydrated in ethanol for 10 min followed by liquid

scintillation counting. Reactions without the substrate were spotted as control and the same process repeated.

**In vitro interaction assays**

Interactions between soluble His-RAD54 and bound GST-BLM were carried out at $4^0$C in 500µl of GST buffer (50mM Tris pH7.5, 100mM KCl, 10mM $MgCl_2$, 5% glycerol and 0.5% NP-40) for 2 hours in absence or presence of C3, C7, C17, after which the beads were washed twice with GST buffer. 10µM of the 3 compounds were used in the in vitro interaction assays.

**Bio-layer interferometry (BLI)**

The interactions between RAD54 and biotinylated BLM_peptide (181-212) as well as the various compounds were studied using Bio-layer interferometry (BLI) using the Octet K2 system (ForteBio Systems). Approximately, 250µg of His-tagged RAD54 was immobilized onto a Ni-NTA chip for 600s and the unbound protein was washed using 10mM HEPES pH 7.5 buffer. Different concentrations of the biotinylated BLM (181-212) peptide (dissolved in water) and C3, C7, and C17 were prepared and loaded in the 96-well plate. Each of the reaction set was then used to check for their association with RAD54 captured on the sensor. The kinetics of the RAD54-BLM interaction was monitored for 180s each for the association and the dissociation curves by dipping the sensors in a series of increasing concentrations of the BLM_peptide. 500mM NaCl was used as the dissociation buffer. A control sensor with the immobilized RAD54 was used in parallel and was used to normalize the data. Washing in 350mM EDTA regenerated the sensors for the next cycle of binding kinetics. The experiments were performed at 25°C. All the real-time data was analyzed and KD values were determined using the Octet® Evaluation software using 1:1 fitting curve.

**Tryptophan fluorescence assay**

Tryptophan fluorescence measurements were performed on a Fluoromax Spectrofluorimeter (Horiba-Jobin Yvon Fluoromax 4). Recombinant His-RAD54 (500nM) was excited at 280nm and the emission intensity monitored at regular intervals from 295nm to atleast 450nm. The emission spectra of RAD54 was determined either (a) alone or (b) in presence of BLM_peptide or SCM_peptide (c) in presence of C3, C7, C17. The concentrations used for RAD54, BLM_peptide, SCM_peptide, C3, C7, C17 are presented in the figure legends.

**MTT assay**

Cells were seeded in a 96-well plate overnight. Next day, the culturing media was replaced with OptiMEM media, followed by incubation with BLM_CPP or SCM_CPP for 1hr. The final concentrations of the peptides were 450nM for cisplatin (CDDP) and 180nM (for all other drugs). The cells were then incubated with 100nM of the C3, C7 and C17 along with gradient of the drugs for 2 hours (for CDDP) or 6 hours (for all other drugs). The IC60 concentrations of C3, C7 and C17 for each cell type was used. Post treatment, the cells were washed with 1XPBS and fresh media was added. After 72 hours, MTT assay was carried out to determine the percentage cell viability of the cells with respect to the untreated cells. Cells without any drug but in presence of C3, C7, C17 were also kept to examine the effect of these compounds alone on the cell viability.

**Alkaline Comet assay**

Alkaline Comet assay was carried out according to the published protocol (11). For the assay, GM03509 GFP-BLM and GM03509 GFP cells were treated with HU treatment (16 hours). Cells released post HU treatment were grown for 6 hours with 180nM of BLM_CPP or

SCM_CPP, following which Comet assays were carried out. For each experiment 2000 cells were used.

**Soft agar assay**

3000 cells mixed with 0.4% agar were seeded on top of the 6-well plates pre-coated with 0.8% agar. For assay in HCT116 BLM -/- cells, the cells were seeded after 2-hour incubation with 180nM of BLM_CPP or SCM_CPP and 6 hours treatment with 120nM of CPT. BLM_CPP or SCM_CPP was replenished after every 24 hrs. For soft agar assay in resistant cells, the cells were treated with 92.6nM of CPT. Post-incubation cells were seeded. The media containing 92.6nM of CPT as well as 100nM of C3, C7, C17 was added at the start and replenished after every 3 days. The colonies were then counted after 15 days after staining with 0.25% Crystal Violet.

**Homologous Recombination Repair and Sister Chromatid Exchange assays**

For Homologous Recombination Repair (HRR) assay, 10µg of the HR plasmid (12) was digested with I-SceI, purified and eluted into 20µl of 10mM Tris HCl pH 8.0. 5µg of the linearized reporter cassette along with 100ng of tdTomato-N1 plasmid were transfected in an exponentially growing 10 cm dish of cells using FuGENE® HD Transfection Reagent. After 3 days of transfection, the cells were harvested. The GFP positive and TdRed positive cells were analysed by FACS. Sister Chromatid Exchange (SCE) assays were carried out as described earlier (13).

**Preparation of lipid nanoparticles of camptothecin (CPT_NPs) and peptides (BLM_NPs, SCM_NPs)**

Egg PC (1 mg), cholesterol (0.3 mg), DSPE-PEG2000-Amine (0.2 mg), Camptothecin (0.5 mg) were mixed in chloroform (0.2 mL) in 1: 0.3: 0.2: 0.5 weight ratio in a round bottom Wheaton glass vial. Chloroform was evaporated by using stream of nitrogen to form a dry thin film. In case of BLM_NPs and SCM_NPs, EggPC (1 mg), cholesterol (0.5 mg), and DSPE-PEG2000-Amine (0.2 mg) were used to form the thin film by above mentioned method, and thin films were kept under vacuum for overnight. Thin films were then hydrated at 4˚C for 6 h with 1 mL saline (for Camptothecin liposomes) or 1 mL saline containing either BLM_peptide or scrambled (SCM) peptide. These lipid suspensions were sonicated in a bath sonicator for 2 min and extruded through 200 nm (14 times each) polycarbonate membranes. Free Camptothecin or peptide was removed by passing through 1 mL Sephadex G-25 bed. Encapsulation efficiency (concentration of Camptothecin or peptide in liposomes) was determined using absorbance method. Liposomes (10 µL) were dissolved in methanol (190 µL), followed by measurement of Camptothecin absorbance at 370 nm or peptide absorbance at 275 nm.

**Hydrogel preparations of Camptothecin and BLM_peptide**

For animal experiments, we used hydrogel based localized delivery of camptothecin and BLM or SCM peptide. We prepared the camptothecin entrapped gel (CPT-Gel), camptothecin and BLM_peptide entrapped gel (CPT-BLM-Gel), and camptothecin and scrambled (SCM) peptide gel (CPT-SCM-Gel) using a lithocholic acid-derived hydrogelator (14). Typically, camptothecin (5mg) and 130mg of gelator in 2ml autoclaved water was heated to form clear solution. For combination hydrogels, 2.5 mg of peptide was added to heated solution containing Camptothecin. Solution was then sonicated and allowed to cool at room temperature to form hydrogel, and 0.2 mL of hydrogel was injected in each mouse near the tumor site.

**MRP2 activity assay**

The efflux activity of MRP2 was determined by measuring the accumulation of the fluorescent MRP2-substrate 5-(6)-carboxy-2′,7′-dichlorofluorescein (CDF) in the HCT116 WT and HCT116 WT IC60CPT$^{R}$ cells in the absence or presence of CPT, C3, C7 and C17 as per the published protocol (15). Briefly, 0.1 million cells were seeded and grown on a 12-well plate for 16 hours, incubated with 92.6nM of CPT either alone or in combination with 10µM of C3, C7 and C17 for 1 hour, followed by treatment 5µM CDF in phenol red free complete media for 30 minutes at 37$^{o}$C. After the incubation, the cells were washed with 1xDPBS twice and resuspended in 1ml of DPBS. The florescence associated with CDF accumulation was measured using Varioskan Flux at an excitation and emission wavelengths of 485nm and 530nm, respectively.

**Immunofluorescence and TUNEL Assay**

To determine the effect of BLM_CPP or SCM_CPP on RAD51, RAD54 and γH2AX foci number, HCT116 BLM-/- cells were treated with HU for 16 hours. Cells were washed with 1XPBS and growth continued for an additional 6 hours with either 180nM BLM_CPP or SCM_CPP. Cells were fixed with 4% paraformaldehyde and processed for immunofluorescence using antibodies against RAD51, RAD54 or γH2AX. Imaging and subsequent analysis was done in a motorized epifluorescence microscope (Upright Axioimager M1; Carl Zeiss) as previously described (16).

For immunofluorescence in tumors, the samples were fixed in 10% neutral buffered formalin and paraffin embedded blocks was prepared. For this purpose, 2-micron tissues sections were first deparaffinized using xylene for 20 minutes, followed by rehydration by immersing in 100%, 90% and 70% ethanol for 10 minutes each, followed by washing with water. The antigen

retrieval was then done using sodium citrate buffer (10mM Sodium Citrate, pH 6.0) in a decloaking chamber (Biocare Medical) for 10 minutes at 95ºC followed by 70ºC for 5 minutes. Tissue sections were permeabilized with 1% goat serum and 0.4% Triton X-100 in PBS, blocked with 5% goat serum in 0.01% PBS supplemented with Tween-20 (PBS-T) for 1 hour at room temperature and stained with Ki67 antibody overnight at 4 ºC. Next day, sections were washed twice with 1% goat serum in PBS-T for 10 minutes each and stained with Alexa 488 labelled secondary antibody for 1 hour. Post two washes, the nuclei were stained with DAPI.

TUNEL assay was carried using Fluorometric TUNEL System according to the manufacturer's instructions. Briefly, the deparaffinised, rehydrated tissue sections were fixed 4% formaldehyde in PBS for 15 minutes and then permeabilized using 100µl of a 20µg/ml Proteinase K solution incubated at room temperature for 8–10 minutes. Following this, the sections were refixed for 5 minutes, equilibrated and then 50µl of TdT reaction mix was added for 60 minutes at 37°C in a humidified chamber. The reaction was stopped by immersing slides in 2X SSC for 15 minutes. The nuclei were the counterstained with DAPI. All imaging was carried out in LSM 510 Meta System (Carl Zeiss, Germany) with 63x/1.4 oil immersion objective. The laser line used was Argon 458/477/488/514 nm.

**Proximity Ligation Assay**

HCT116 and CCD 841 CoN cells were grown on 12mm coverslips in 12 well plates for 24 hours. After washing with 1XPBS, cells were treated with a hypotonic lysis buffer containing 10 mM Tris-HCl pH 7.4, 2.5 mM $MgCl_2$, 1 mM PMSF, and 0.5 % Nonidet P-40 on ice for 8 min. They were then rinsed with 1XPBS and fixed with ice-cold absolute ethanol. After washing again with 1XPBS, the cells were permeabilized with 1XPBS containing 0.2% Tween 20 for 10 min on ice. Proximity Ligation Assay (PLA) was performed using Duolink In Situ Red Starter Kit Mouse/Rabbit (Sigma-Aldrich) following the manufacturer's instructions.

Images were captured using a confocal microscope (Zeiss LSM980) with a 63x oil objective lens.

# Supplemental Figures and Figure Legends

Figure S1
Kaur et al.,

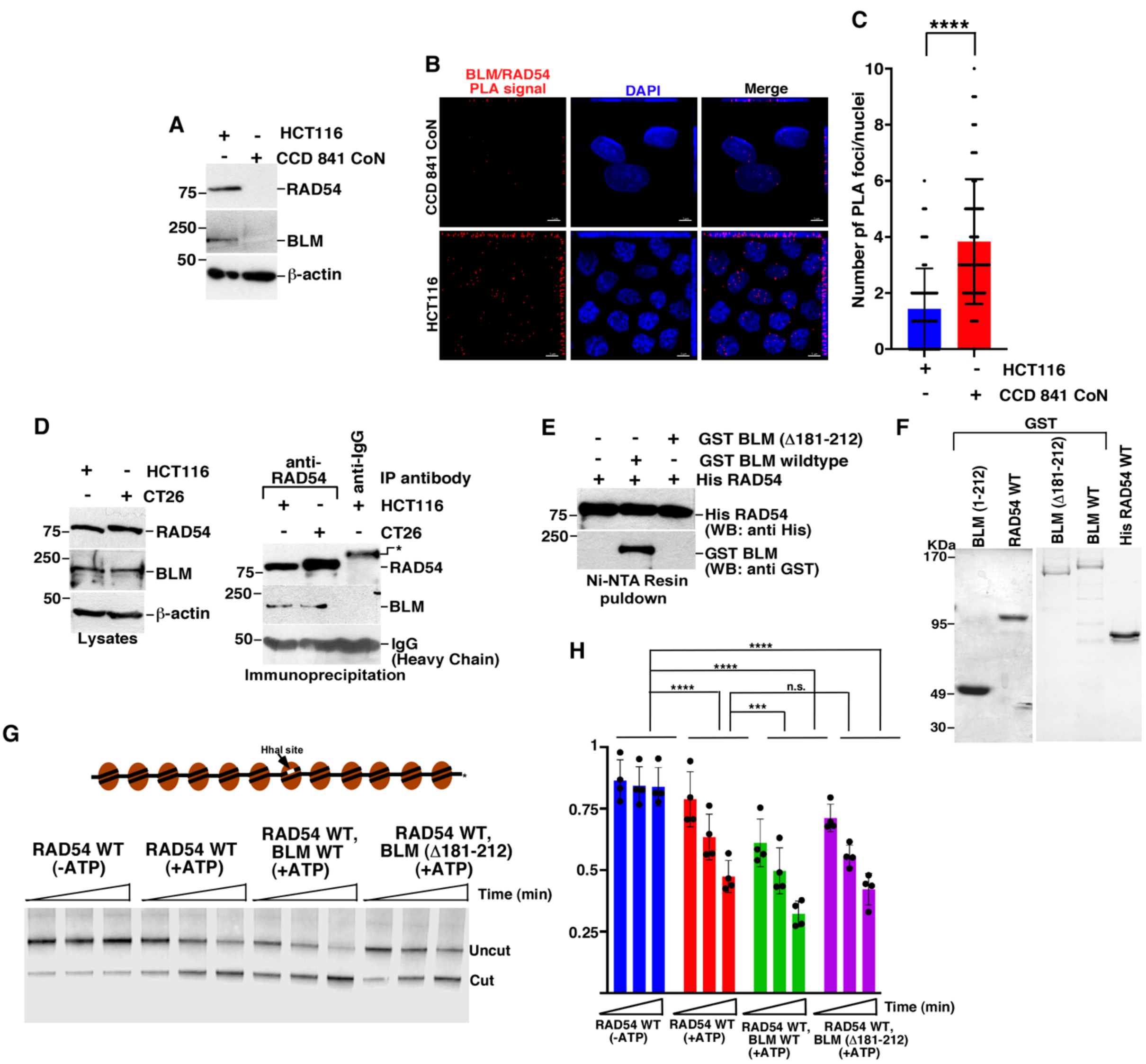


**Figure S1: BLM (181-212) plays a key role in BLM-RAD54 interaction.**

A-C. BLM-RAD54 interaction was greater in cancer cells than normal cells. HCT116 (derived from colon cancer) and CCD 841 CoN (derived from normal colon epithelium) were used. (A) Lysates made from both cell types were subjected to immunoblotting with anti-BLM and anti-RAD54 antibodies. (B, C) Proximity Ligation Assay (PLA) was carried out with antibodies against BLM and RAD54, followed by immunofluorescence and imaging in a confocal

microscope. Representative image is shown in (B). Bar 5μM. (C) Quantitation of (B). Mean ± S.D. Total number of cells analyzed in each case was 78 over two biological replicates. Statistical test: Unpaired t-test.

D. BLM-RAD54 interaction occur in both human HCT116 and murine CT26 cells. (Left) Lysates made from HCT116, CT26 cells were probed for the expression of RAD54 and BLM. (Right) Lysates were immunoprecipitated with anti-RAD54 antibodies and immunoprecipitates probed with anti-BLM antibodies. The experiment was repeated three times. One representative experiment has been shown. (*) represents a cross-reactive band.

E. Lack of amino acids (181-212) in BLM abrogates its interacts with RAD54 in vitro. Bound His-RAD54 was incubated with soluble GST BLM wildtype or GST BLM (Δ181-212). The protein bound to His-RAD54 was detected by using anti-BLM antibody. The experiment was repeated three times. One representative experiment has been shown.

F. Purified proteins used in biochemical and biophysical assays. Coomassie gels indicating the purified GST BLM (1-212), GST RAD54 (1-747) (WT), GST BLM (1-1417) (WT), GST BLM (Δ181-212), His RAD54 (1-747) (WT). The purification of each protein was repeated at least five times.

G, H. BLM (Δ181-212) did not enhance ATP-dependent RAD54 mediated chromatin remodeling. (G, Top) Schematic diagram of G5E4 array consisting of 12 nucleosomes with a centrally located HhaI site occluded by one nucleosome. (G, Bottom) REA assays were carried out with chromatinized G5E4 array using the experimental conditions as indicated. The reactions were stopped after 0 minute, 5 minutes and 10 minutes. (H) Quantitation of (G). The

fraction uncut is presented. The data is from three independent experiments. Mean ± S.D. Statistical test: 2way ANOVA.

Figure S2
Kaur et al.,

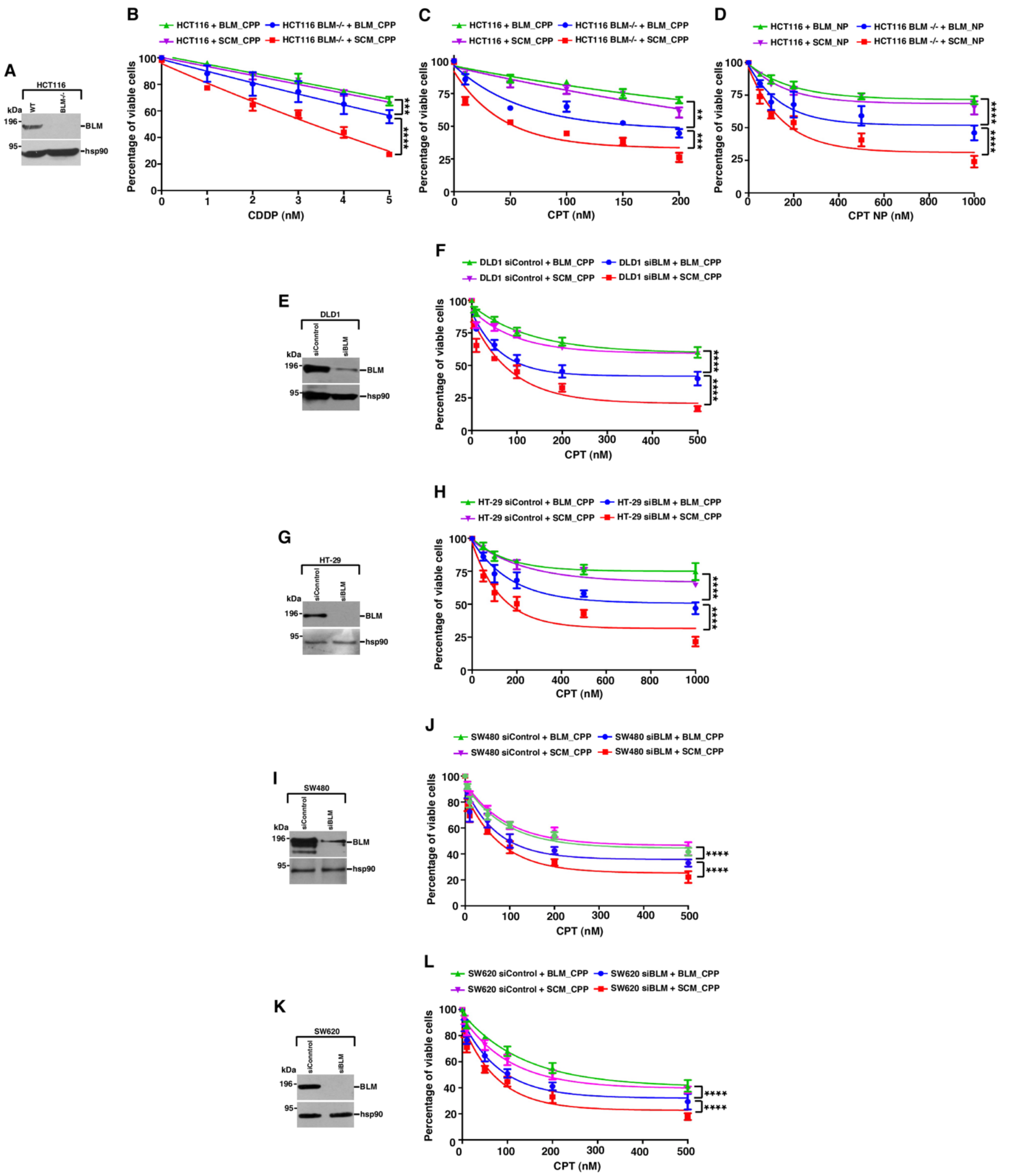


**Figure S2: BLM_CPP increases chemoresistance in colon cancer cells**

A, E, G, I, K. Loss or depletion of BLM in colon cancer cells. Lysates were made from (A) HCT116 WT, HCT116 BLM-/-, (E) DLD1 treated with either siControl or siBLM, (G) HT-29

treated with either siControl or siBLM, (I) SW480 treated with either siControl or siBLM and (K) SW620 treated with either siControl or siBLM, Western analysis was carried out with antibodies against BLM, hsp90. All the experiments were repeated three times and one representative experiment has been shown in each case.

B-D. BLM_CPP and BLM_NP increased cellular resistance to cisplatin and camptothecin in HCT116 isogenic system. HCT116 WT and HCT116 BLM-/- cells were treated with (B, C) 180nM of BLM_CPP or SCM_CPP or (D) BLM_NP or SCM_NP in presence of (B) 1nM, 2nM, 3nM, 4nM, 5nM of CDDP, (C) 10nM, 50nM, 100nM, 150nM, 200nM of CPT, (D) 50nM, 100nM, 200nM, 500nM, 1000nM of CPT. The percentage of viable cells were determined by MTT assays. The data is from three independent experiments. Mean ± S.D. Statistical test for (B-D): 2way ANOVA.

F, H, J, L. BLM_CPP increased cellular resistance to camptothecin in isogenic systems created in multiple colon cancer cell lines. Isogenic systems based on BLM depletion were created in (F) DLD1, (H) HT-29, (J) SW480, (L) SW620. The isogenic lines were treated with (F, J, L) 10nM, 50nM, 100nM, 200nM, 500nM of CPT, (H) 50nM, 100nM, 200nM, 500nM, 1000nM of CPT. The percentage of viable cells were determined by MTT assays. The data is from three independent experiments. Mean ± S.D. Statistical test for (F, H, J, L): 2way ANOVA.

Figure S3
Kaur et al.,

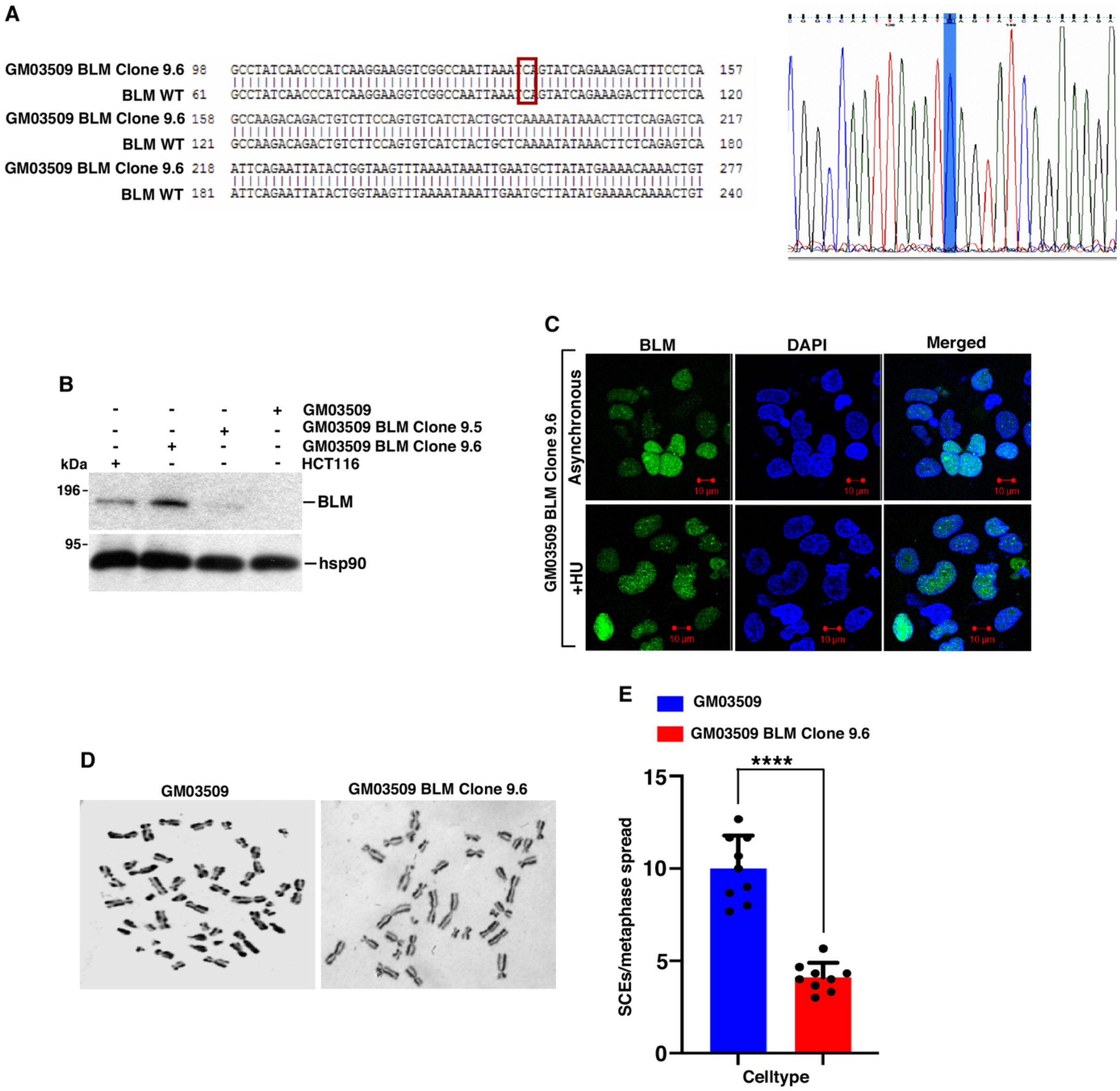


**Figure S3: Functional BLM is expressed in corrected GM03509 BLM Clone 9.6 cells**

A. BLM mutation is absent in GM03509 BLM Clone 9.6 cells. PCR product containing the genomic DNA from GM03509 BLM Clone 9.6 cells spanning the mutated site was cloned into TA vector. Sanger sequencing was carried out in 20 sub-clones. Result from a representative sub-clone is presented. (Left) Multiple alignment is shown for the sequence obtained from GM03509 BLM Clone 9.6 cells and that of wildtype BLM. The red rectangle shows the corrected nucleotide. (Right) The corresponding chromatogram as obtained after Sanger sequencing. The blue shaded rectangle shows the corrected nucleotide.

B. GM03509 BLM Clone 9.6 cells express BLM. Lysates were made from GM03509 BLM Clone 9.6 cells (GM03509 cells where BLM mutation has been corrected), GM03509 BLM Clone 9.5 cells (GM03509 cells where BLM mutation was not corrected completely, as a very low level of BLM expression is observed), GM03509 (parental BS fibroblast) and HCT116 WT cells. Western blot analysis was carried out using antibodies against BLM and hsp90. The experiment was repeated three times and a one replicate is shown.

C. GM03509 BLM Clone 9.6 cells form BLM foci post HU treatment. GM03509 BLM Clone 9.6 cells were treated with 1mM of HU for 16 hours. Cells were fixed and processed for immunofluorescence with anti-BLM antibody. The experiment was repeated three times and a representative image is shown. Bar 10µM

D, E. Correction of BLM mutation in GM03509 BLM Clone 9.6 cells decreases SCE levels. SCEs were carried out in GM03509 and GM03509 BLM Clone 9.6 cells. (D) Representative images are shown. (E) Quantitation of (D). The data is from three independent experiments (n=9 in each experiment). Mean ± S.D. Statistical test: Unpaired t-test.

Figure S4
Kaur et al.,

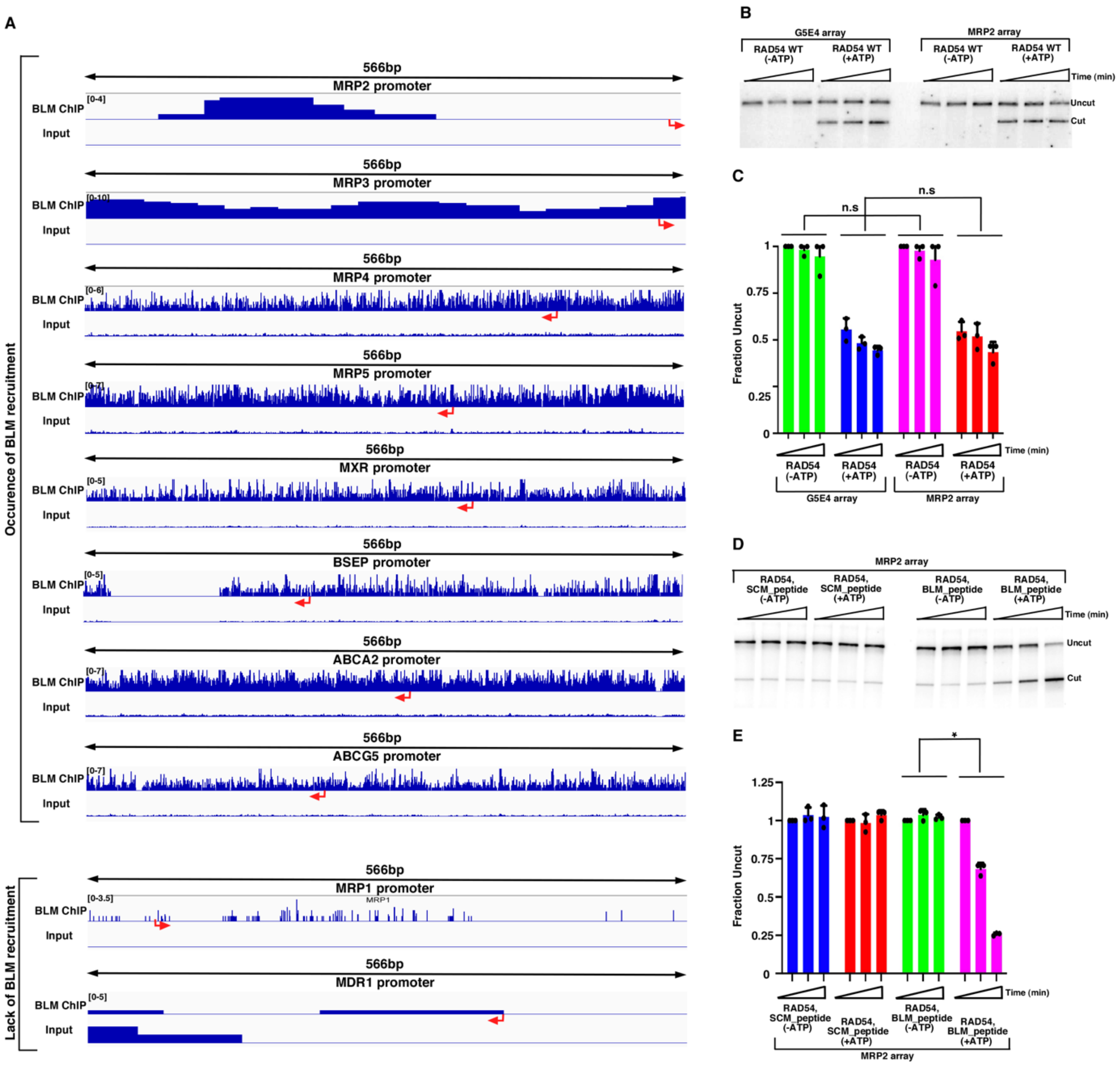


**Figure S4: BLM is recruited to the promoters of MDR genes.**

A. IGV browser tracks from BLM peaks and input signals around the TSS of (Top) MDR gene promoters where BLM is recruited: *MRP2*, *MRP3*, *MRP4*, *MRP5*, *MXR*, *BSEP*, *ABCA2* and *ABCG5* (Bottom) MDR gene promoters where BLM is not recruited: *MRP1*, *MDR1*. The interval range is indicated in brackets on left side of each track.

B, C. RAD54 enhanced chromatin remodeling equally on G5E4 and *MRP2* promoter arrays. (B) REA assays were carried out with chromatinized G5E4 or *MRP2* array using the following

experimental conditions: RAD54-ATP, RAD54 +ATP. The reactions were stopped after 1 minute, 5 minutes and 10 minutes. (C) Quantitation of (B). The fraction uncut is presented. The data was from three independent experiments. Mean ± S.D. Statistical test: 2way ANOVA.

D, E. BLM_peptide enhanced RAD54 mediated chromatin remodeling on MRP2 arrays. (D) REA assays were carried out with chromatinized MRP2 array using the following experimental conditions: RAD54 + SCM-peptide (-ATP), RAD54 + SCM-peptide (+ATP), RAD54 + BLM-peptide (-ATP), RAD54 + BLM-peptide (+ATP). The reactions were stopped after 1 minute, 5 minutes and 10 minutes. (E) Quantitation of (D). The fraction uncut is presented. The data was from three independent experiments. Mean ± S.D. Statistical test: 2way ANOVA.

Figure S5
Kaur et al.,

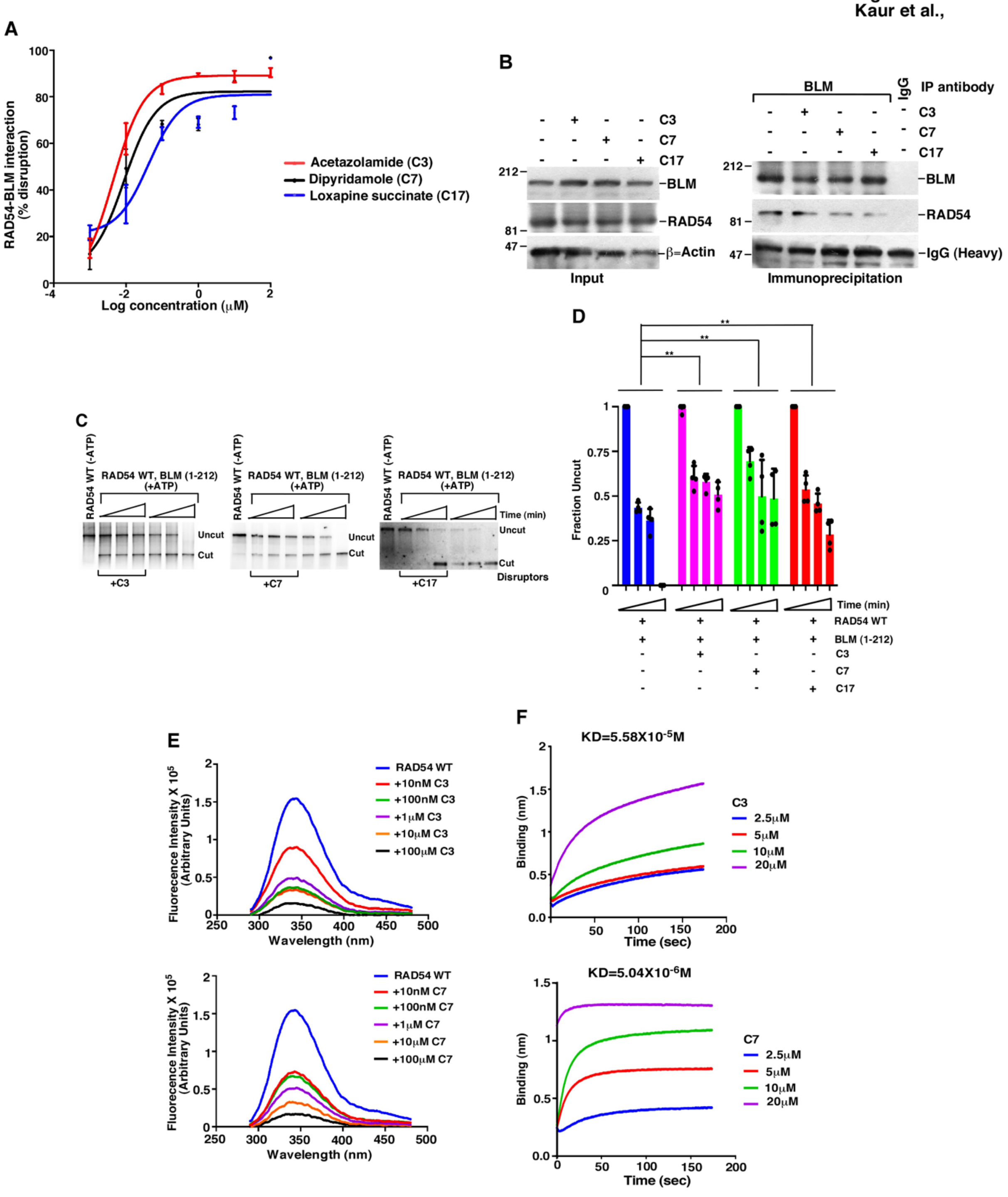


**Figure S5: C3, C7, C17 prevent BLM-RAD54 interaction.**

A. Dose response curve for C3, C7, C17 when tested for BLM-RAD54 complex disruption. Using PCA, dose response curve for C3, C7, C17 were determined using a range of concentrations (1nM, 10nM, 100nM, 1μM, 10μM and 100μM) of the three small molecules.

The percentage disruption of the interaction between BLM F2 and N-RAD54 F1 was plotted as a non-linear regression line. The data was from three independent experiments. Mean ± S.D.

B. C3, C7, C17 disrupt BLM-RAD54 interaction in cells. Lysates were made from HEK293T cells either left untreated or treated with 100nM C3, C7, C17 for 72 hours. (Left) Direct westerns were carried out with antibodies against BLM, RAD54, β−Actin. (Right) immunoprecipitation (IP) was carried out with anti-BLM antibody. Westerns were carried out with anti-BLM, anti-RAD54 antibodies. The experiment was repeated three times and one representative experiment has been shown.

C, D. C3, C7, C17 decrease the efficiency of BLM dependent enhancement of RAD54 chromatin remodeling activity. (C) REA assays were carried out with chromatinized G5E4 array using the following experimental conditions: RAD54 WT + BLM (1-212), RAD54 WT + BLM (1-212) + C3, RAD54 WT + BLM (1-212) + C7, RAD54 WT + BLM (1-212) + C17. All reactions were carried out in presence of ATP. The reactions were stopped after 1 minute, 5 minutes and 10 minutes. (D) Quantitation of (C). The fraction uncut is presented. The data is from three independent experiments. Mean ± S.D. Statistical test: 2way ANOVA.

E. C3, C7 alter the conformation of RAD54. Tryptophan fluorescence assays were carried out with His-RAD54 WT, either alone or in presence of the indicated concentrations of C3, C7. RAD54 fluorescence was measured in a fluorometer. The experiment was repeated three times and one representative experiment has been shown.

F. C3, C7 can bind to RAD54. Octet BLI based studies were performed to determine the dissociation constant of the interaction with the indicated concentrations of C3, C7 with His-

RAD54 WT immobilized onto Nickel-sensor. The affinity constant (KD) ± SD is shown in both cases. The experiment was repeated three times for both C3, C7. One representative experiment has been shown.

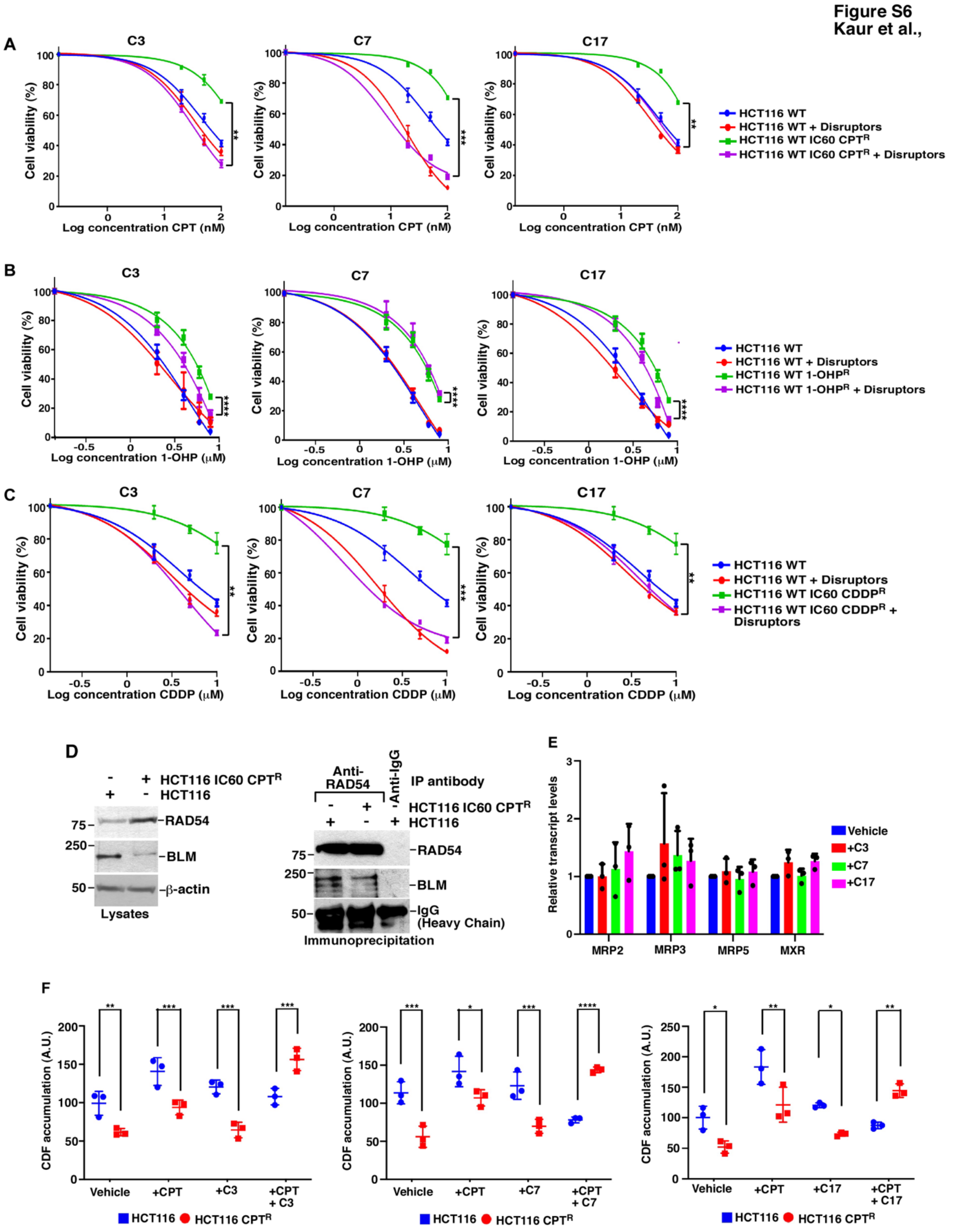


**Figure S6: C3, C7, C17 increased the sensitivity of resistant cells by enhancing drug accumulation by decreasing MRP2 activity.**

A-C. Treatment of C3, C7, C17 decrease drug resistance to multiple chemotherapeutic agents. (A) HCT116 IC60 $CPT^R$ or HCT116 cells (B) HCT116 IC60 1-$OHP^R$ or HCT116 cells (C) HCT116 IC60 $CDDP^R$ or HCT116 cells were treated with 100nM of C3, C7, C17 along with increasing concentrations of (A) CPT or (B) 1-OHP (C) CDDP for 72 hours. Concentrations of CPT used: 0.140nM, 20nM, 50nM, 1000nM. Concentration of 1-OHP: 0.14μM, 2μM, 4μM, 6μM, 8μM. Concentrations of CDDP used: 0.140μM, 2μM, 5μM, 10μM. The percentage of viable cells were determined by MTT assays. The data is from (A, C) three (B) nine independent experiments. Mean ± S.D. Statistical test for (A-C): 2way ANOVA.

D. BLM-RAD54 interaction is decreased in HCT116 IC60 $CPT^R$ cells. (Left) Lysates were made from the parental HCT116 and HCT116 IC60 $CPT^R$ cells. (Right) Immunoprecipitation (IP) was carried out with anti-RAD54 antibodies and the immunoprecipitates were probed with anti-BLM antibody. The experiment was repeated three times and a one replicate is shown.

E. Small molecules do not alter the transcript levels of MDR genes in HCT116 IC60 $CPT^R$ cells. RNA isolated from HCT116 IC60 $CPT^R$ cells after 72 hours treatment with 100nM of C3, C7 and C17 were used for RT-qPCR. The levels of MRP2, MRP3, MRP5, MXR were quantitated from three independent experiments. Mean ± S.D. Statistical test: 2way ANOVA.

F. C3/C7/C17 along with CPT decrease MRP2 activity in HCT116 WT IC60 $CPT^R$ cells. HCT116 WT and HCT116 WT IC60 $CPT^R$ cells were seeded and were either left untreated, or treated with CPT or C3/C7/C17 alone or CPT + C3/C7/C17. The cells were incubated with MRP2 substrate CDF for 30 minutes at $37^0$C. The accumulation of florescent product (CDF)

was determined as a measure of MRP2 activity. The data is from three independent experiments. Mean ± S.D. Statistical test: 2way ANOVA.

Figure S7
Kaur et al.,

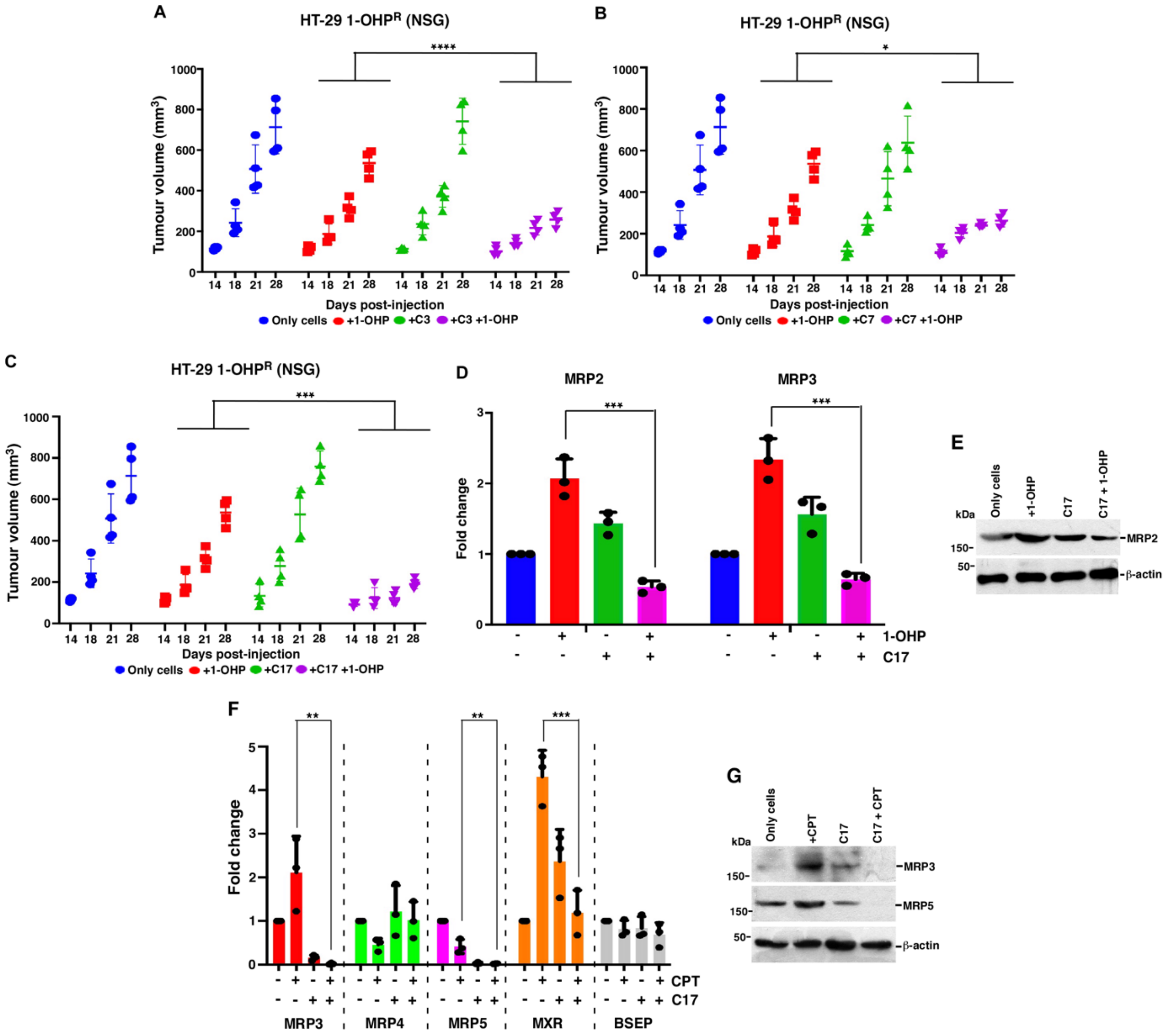


**Figure S7: C3, C7, C17 enhanced the effect of CPT and 1-OHP mediated decrease in tumor volume via MDR pathway.**

A-C. C3, C7, C17 decreased tumor formation by oxaliplatin resistant cells in HT-29 1-OHP$^R$ cells derived xenograft model. HT-29 1-OHP$^R$ cells were injected into NSG mice (n=3 in each group). The groups were made as indicated. The volume of the tumors was estimated for the

indicated days. Mean ± S.D. Data for C3 is shown in (A), C7 in (B) and C17 in (C). Statistical test for (A-C): 2way ANOVA.

D, E. Treatment with both CPT and C17 decreased MDR transcript and protein levels in tumors made with HT-29 1-OHP$^R$ cells. (D) RNA and (E) protein was isolated from tumors obtained at the end point of the xenograft experiment. RNA and the protein levels of the indicated MDR genes were determined by (D) RT-qPCR for MRP2, MRP3 and (E) western blotting with anti-MRP2 antibody. For each group three tumor samples were analyzed. Data for (D) is from three mice. Mean ± S.D. Data for (E) is from one mouse and is representative of the three mice analyzed. Statistical test for (D): 2way ANOVA.

F. Treatment with both CPT and C17 decreased the transcript levels of multiple MDR genes in tumors obtained with HCT116 IC60 CPT$^R$ cells. RNA was isolated from tumors obtained by injecting only HCT116 IC60 CPT$^R$ cells, tumors treated with CPT (1.25mg/kg), tumors treated with C17 (5mg/kg), tumors treated with CPT and C17. The levels of the indicated MDR genes were quantitated from three tumor samples from each group. Mean ± S.D. Statistical test: One-way ANOVA.

G. Treatment with both CPT and C17 decreased MDR protein levels in tumors obtained with HCT116 IC60 CPT$^R$ cells. Lysates were made from tumors obtained by injecting only HCT116 WTIC60 CPT$^R$ cells, tumors treated with CPT (1.25mg/kg), tumors treated with C17 (5mg/kg), tumors treated with CPT and C17 and probed with antibodies against MRP3, MRP5 and β-actin. The experiment was repeated three times and one representative experiment has been shown.

Figure S8
Kaur et al.,

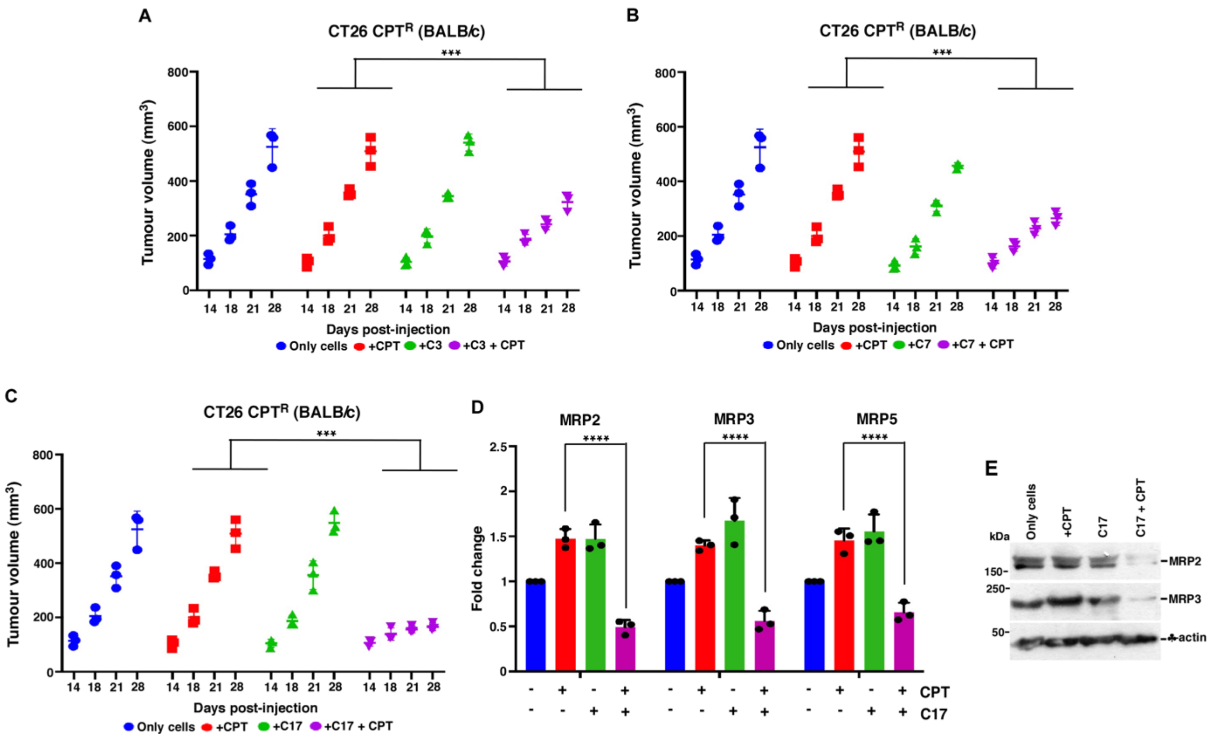


**Figure S8: C3, C7, C17 enhanced the effect of CPT mediated decrease in tumor volume in a syngeneic mice model.**

A-C. C3, C7, C17 decreased tumor formation by camptothecin resistant murine CT26 $CPT^R$ cells in a syngeneic mice model. CT26 $CPT^R$ cells were injected into BALB/c mice (n=3 in each group). The groups were made as indicated. The volume of the tumors was estimated for the indicated days. Mean ± S.D. Data for C3 is shown in (A), C7 in (B) and C17 in (C). Statistical test for (A-C): 2way ANOVA.

D, E. Treatment with both CPT and C17 decreased MDR transcript and protein levels in tumors made with CT26 $CPT^R$ cells. (D) RNA and (E) protein was isolated from tumors obtained at the end point of the syngeneic mice experiment. RNA and the protein levels of the indicated MDR genes were determined by (D) RT-qPCR and (E) western blotting with anti-MRP2, anti-MRP3 antibodies. For each group three tumor samples were analyzed. Data for (D) is from

three mice. Mean ± S.D. Data for (E) is from one mouse and is representative of the three mice analyzed. Statistical test for (D): 2way ANOVA.

Figure S9
Kaur et al.,

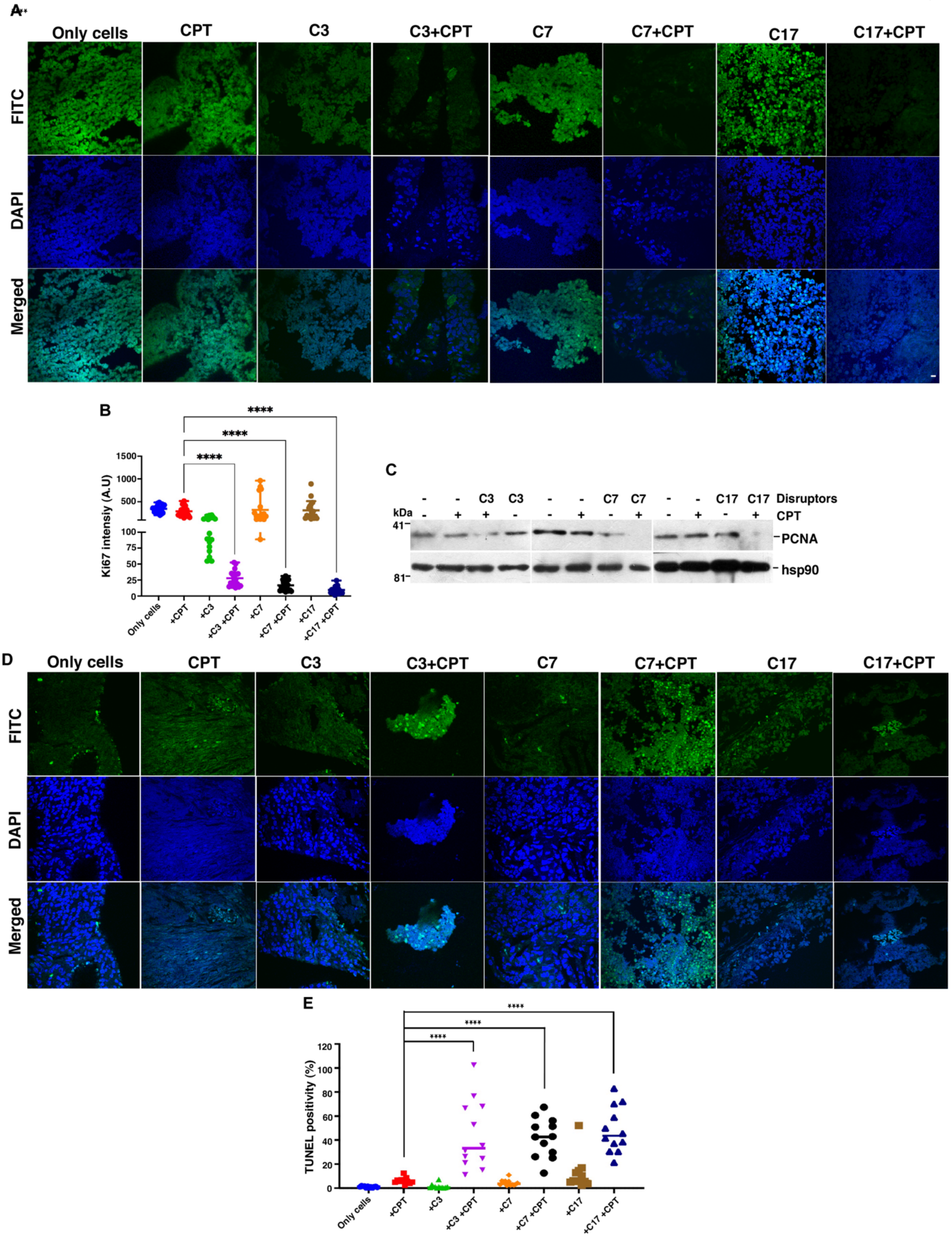


**Figure S9: Combination of C3, C7, C17 with camptothecin decreased proliferation and increased apoptosis in murine xenograft models.**

A-C. C3, C7, C17 treatment decreased proliferation and increased apoptosis in the xenografted tumors derived from HCT116 IC60 $CPT^R$ cells. Tumors were obtained from mice xenografted with HCT116 IC60 $CPT^R$ cells at the endpoint of the experiment. The tumors were obtained from the following groups: tumors made injecting only cells, tumors treated with CPT (1.25mg/kg) alone, tumors treated with C3/C7/C17 (all 5mg/kg), tumors treated with CPT and C3/C7C17. Sections from FFPE embedded tumors were (A) stained with anti-Ki67 antibody. Representative images are shown for one set of tumours with all different conditions. (B) Quantitation of cells Ki67 positive cells from sections imaged from six mice. The staining intensity was determined by ImageJ software. Mean ± S.D. Statistical test: One-way ANOVA. (C) Lysates made from the same tumours were probed with antibodies against PCNA and hsp90.

(D) Same as (A) except the tissues were subjected to TUNEL assay. Representative images are shown. (E) Number of positive cells per area in $\mu m^2$ depicting the TUNEL positive apoptotic cells determined (n=12). Mean ± S.D. Statistical test: One-way ANOVA.

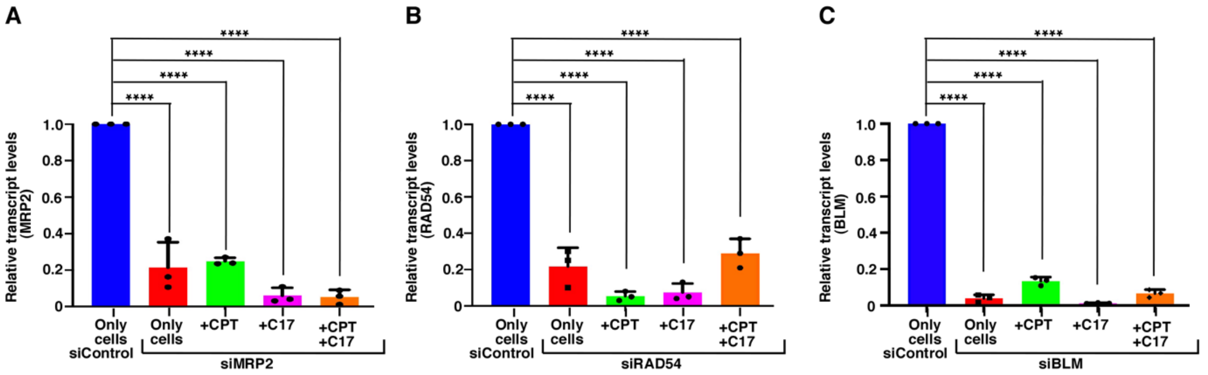


**Figure S10: Introduction of siMRP2, siRAD54, siBLM in xenograft tumors can ablate the expression of their cognate genes.**

SCID mice were used to generate tumors by injecting only HCT116 WT IC60 $CPT^R$ cells. Tumors were were either left untreated or treated with CPT (1.25mg/kg) or treated with C17 (5mg/kg) or treated with both CPT CPT (1.25mg/kg) and C17 (5mg/kg). When the tumors reach $50mm^3$, they were injected with either siControl or siMRP2 or siRAD54 or siBLM (as indicated) using TAC6 polymer mediated in vivo delivery. At the end-point of the experiment tumors were excised, RNA isolated and RT-qPCR carried out for (A) MRP2, (B) RAD54, (C) BLM. The data is from three tumors in each group. Mean ± S.D. Statistical test: One-way ANOVA.

# Supplemental Tables

**Table S1: Nucleotide sequence of pUC57-MRP2 used for Restriction Enzyme Accessibility (REA) assay**

TCGCGCGTTTCGGTGATGACGGTGAAAACCTCTGACACATGCAGCTCCCGGAGA
CGGTCACAGCTTGTCTGTAAGCGGATGCCGGGAGCAGACAAGCCCGTCAGGGCG
CGTCAGCGGGTGTTGGCGGGTGTCGGGGCTGGCTTAACTATGCGGCATCAGAGC
AGATTGTACTGAGAGTGCACCATATGCGGTGTGAAATACCGCACAGATGCGTAA
GGAGAAAATACCGCATCAGGCGCCATTCGCCATTCAGGCTGCGCAACTGTTGGG
AAGGGCGATCGGTGCGGGCCTCTTCGCTATTACGCCAGCTGGCGAAAGGGGGAT
GTGCTGCAAGGCGATTAAGTTGGGTAACGCCAGGGTTTTCCCAGTCACGACGTTG
TAAAACGACGGCCAGTGAATTCGAGCTCGGTACCTCGCGAATGCATCTAGATCC
GCATGGGTGGTTCATGATATCATGAAAAAAGCATGTTCTTAAAGCAATTTAAGTG
ACAGTACAAAAGGTTGGGTCAGGTGGGGCACGGTAGCTCATGCCTGCAATCCCA
GCCCTTTGGGAGGCCAAGGCAGAAGGATTGTTGAAGCCTGGAGTTTGAGACCAG
CCTGGGCAACATAGTGAGACCCCGTCCCTACAGAAAACATTTTCCGCATGGGTG
GTTCATGATATCATGAAAAAAGCATGTTCTTAAAGCAATTTAAGTGACAGTACAA
AAGGTTGGGTCAGGTGGGGCACGGTAGCTCATGCCTGCAATCCCAGCCCTTTGG
GAGGCCAAGGCAGAAGGATTGTTGAAGCCTGGAGTTTGAGACCAGCCTGGGCAA
CATAGTGAGACCCCGTCCCTACAGAAAACATTTTCCGCATGGGTGGTTCATGATA
TCATGAAAAAAGCATGTTCTTAAAGCAATTTAAGTGACAGTACAAAAGGTTGGG
TCAGGTGGGGCACGGTAGCTCATGCCTGCAATCCCAGCCCTTTGGGAGGCCAAG
GCAGAAGGATTGTTGAAGCCTGGAGTTTGAGACCAGCCTGGGCAACATAGTGAG
ACCCCGTCCCTACAGAAAACATTTTCCGCATGGGTGGTTCATGATATCATGAAAA
AAGCATGTTCTTAAAGCAATTTAAGTGACAGTACAAAAGGTTGGGTCAGGTGGG
GCACGGTAGCTCATGCCTGCAATCCCAGCCCTTTGGGAGGCCAAGGCAGAAGGA
TTGTTGAAGCCTGGAGTTTGAGACCAGCCTGGGCAACATAGTGAGACCCCGTCCC
TACAGAAAACATTTTCCGCATGGGTGGTTCATGATATCATGAAAAAAGCATGTTC
TTAAAGCAATTTAAGTGACAGTACAAAAGGTTGGGTCAGGTGGGGCACGGTAGC
TCATGCCTGCAATCCCAGCCCTTTGGGAGGCCAAGGCAGAAGGATTGTTGAAGC
CTGGAGTTTGAGACCAGCCTGGGCAACATAGTGAGACCCCGTCCCTACAGAAAA
CATTTTCCGAATCTTTAAACTCGAGTGCATGCCTGCAGGTCCTCGGAGGACAGTA
CTCCGCTCGGAGGACAGTACTCCGCTCGGAGGACAGTACTCCGCTCGGAGGACA
GTACTCCGCTCGGAGGACAGTACTCCGACTCTAGAGGATCCCCAGTCCTATATAT
ACTCGCTCTGCACTTGGCCCTTTTTTACACTGTGACTGATTGAGCTGGTGCCGTGT
CGAGTGGTGTTTTTTTAATAGGTTTTCTTTTTTACTGGTAAGGCTGACTGTTATGG
CTGCCGCTGTGGAAGCGCTGTATGTTGTTCTGGAGCGGGAGGGTGCTATTTTGCC
TAGGCAGGAGGGTTTTTCAGGTGTTTATGTGTTTTTCTCTCCTATTAATTTTGTTA
TACCTCCTATGGGGGCTGTAATCTCGAGCTCGCTGGATCCGCATGGGTGGTTCAT
GATATCATGAAAAAAGCATGTTCTTAAAGCAATTTAAGTGACAGTACAAAAGGT
TGGGTCAGGTGGGGCACGGTAGCTCATGCCTGCAATCCCAGCCCTTTGGGAGGC
CAAGGCAGAAGGATTGTTGAAGCCTGGAGTTTGAGACCAGCCTGGGCAACATAG
TGAGACCCCGTCCCTACAGAAAACATTTTCCGCATGGGTGGTTCATGATATCATG
AAAAAAGCATGTTCTTAAAGCAATTTAAGTGACAGTACAAAAGGTTGGGTCAGG
TGGGGCACGGTAGCTCATGCCTGCAATCCCAGCCCTTTGGGAGGCCAAGGCAGA
AGGATTGTTGAAGCCTGGAGTTTGAGACCAGCCTGGGCAACATAGTGAGACCCC
GTCCCTACAGAAAACATTTTCCGCATGGGTGGTTCATGATATCATGAAAAAAGCA

TGTTCTTAAAGCAATTTAAGTGACAGTACAAAAGGTTGGGTCAGGTGGGGCACG
GTAGCTCATGCCTGCAATCCCAGCCCTTTGGGAGGCCAAGGCAGAAGGATTGTT
GAAGCCTGGAGTTTGAGACCAGCCTGGGCAACATAGTGAGACCCCGTCCCTACA
GAAAACATTTTCCGCATGGGTGGTTCATGATATCATGAAAAAAGCATGTTCTTAA
AGCAATTTAAGTGACAGTACAAAAGGTTGGGTCAGGTGGGGCACGGTAGCTCAT
GCCTGCAATCCCAGCCCTTTGGGAGGCCAAGGCAGAAGGATTGTTGAAGCCTGG
AGTTTGAGACCAGCCTGGGCAACATAGTGAGACCCCGTCCCTACAGAAAACATT
TTCCGCATGGGTGGTTCATGATATCATGAAAAAAGCATGTTCTTAAAGCAATTTA
AGTGACAGTACAAAAGGTTGGGTCAGGTGGGGCACGGTAGCTCATGCCTGCAAT
CCCAGCCCTTTGGGAGGCCAAGGCAGAAGGATTGTTGAAGCCTGGAGTTTGAGA
CCAGCCTGGGCAACATAGTGAGACCCCGTCCCTACAGAAAACATTTTATCGGATC
CCGGGCCCGTCGACTGCAGAGGCCTGCATGCAAGCTTGGCGTAATCATGGTCAT
AGCTGTTTCCTGTGTGAAATTGTTATCCGCTCACAATTCCACACAACATACGAGC
CGGAAGCATAAAGTGTAAAGCCTGGGGTGCCTAATGAGTGAGCTAACTCACATT
AATTGCGTTGCGCTCACTGCCCGCTTTCCAGTCGGGAAACCTGTCGTGCCAGCTG
CATTAATGAATCGGCCAACGCGCGGGGAGAGGCGGTTTGCGTATTGGGCGCTCT
TCCGCTTCCTCGCTCACTGACTCGCTGCGCTCGGTCGTTCGGCTGCGGCGAGCGG
TATCAGCTCACTCAAAGGCGGTAATACGGTTATCCACAGAATCAGGGGATAACG
CAGGAAAGAACATGTGAGCAAAAGGCCAGCAAAAGGCCAGGAACCGTAAAAAG
GCCGCGTTGCTGGCGTTTTTCCATAGGCTCCGCCCCCCTGACGAGCATCACAAAA
ATCGACGCTCAAGTCAGAGGTGGCGAAACCCGACAGGACTATAAAGATACCAGG
CGTTTCCCCCTGGAAGCTCCCTCGTGCGCTCTCCTGTTCCGACCCTGCCGCTTACC
GGATACCTGTCCGCCTTTCTCCCTTCGGGAAGCGTGGCGCTTTCTCATAGCTCAC
GCTGTAGGTATCTCAGTTCGGTGTAGGTCGTTCGCTCCAAGCTGGGCTGTGTGCA
CGAACCCCCCGTTCAGCCCGACCGCTGCGCCTTATCCGGTAACTATCGTCTTGAG
TCCAACCCGGTAAGACACGACTTATCGCCACTGGCAGCAGCCACTGGTAACAGG
ATTAGCAGAGCGAGGTATGTAGGCGGTGCTACAGAGTTCTTGAAGTGGTGGCCT
AACTACGGCTACACTAGAAGAACAGTATTTGGTATCTGCGCTCTGCTGAAGCCAG
TTACCTTCGGAAAAAGAGTTGGTAGCTCTTGATCCGGCAAACAAACCACCGCTG
GTAGCGGTGGTTTTTTTGTTTGCAAGCAGCAGATTACGCGCAGAAAAAAAGGAT
CTCAAGAAGATCCTTTGATCTTTTCTACGGGGTCTGACGCTCAGTGGAACGAAAA
CTCACGTTAAGGGATTTTGGTCATGAGATTATCAAAAAGGATCTTCACCTAGATC
CTTTTAAATTAAAAATGAAGTTTTAAATCAATCTAAAGTATATATGAGTAAACTT
GGTCTGACAGTTACCAATGCTTAATCAGTGAGGCACCTATCTCAGCGATCTGTCT
ATTTCGTTCATCCATAGTTGCCTGACTCCCCGTCGTGTAGATAACTACGATACGG
GAGGGCTTACCATCTGGCCCCAGTGCTGCAATGATACCGCGAGACCCACGCTCA
CCGGCTCCAGATTTATCAGCAATAAACCAGCCAGCCGGAAGGGCCGAGCGCAGA
AGTGGTCCTGCAACTTTATCCGCCTCCATCCAGTCTATTAATTGTTGCCGGGAAG
CTAGAGTAAGTAGTTCGCCAGTTAATAGTTTGCGCAACGTTGTTGCCATTGCTAC
AGGCATCGTGGTGTCACGCTCGTCGTTTGGTATGGCTTCATTCAGCTCCGGTTCCC
AACGATCAAGGCGAGTTACATGATCCCCCATGTTGTGCAAAAAAGCGGTTAGCT
CCTTCGGTCCTCCGATCGTTGTCAGAAGTAAGTTGGCCGCAGTGTTATCACTCAT
GGTTATGGCAGCACTGCATAATTCTCTTACTGTCATGCCATCCGTAAGATGCTTTT
CTGTGACTGGTGAGTACTCAACCAAGTCATTCTGAGAATAGTGTATGCGGCGACC
GAGTTGCTCTTGCCCGGCGTCAATACGGGATAATACCGCGCCACATAGCAGAAC
TTTAAAAGTGCTCATCATTGGAAAACGTTCTTCGGGGCGAAAACTCTCAAGGATC
TTACCGCTGTTGAGATCCAGTTCGATGTAACCCACTCGTGCACCCAACTGATCTT
CAGCATCTTTTACTTTCACCAGCGTTTCTGGGTGAGCAAAAACAGGAAGGCAAA
ATGCCGCAAAAAAGGGAATAAGGGCGACACGGAAATGTTGAATACTCATACTCT
TCCTTTTTCAATATTATTGAAGCATTTATCAGGGTTATTGTCTCATGAGCGGATAC

ATATTTGAATGTATTTAGAAAAATAAACAAATAGGGGTTCCGCGCACATTTCCCC
GAAAAGTGCCACCTGACGTCTAAGAAACCATTATTATCATGACATTAACCTATAA
AAATAGGCGTATCACGAGGCCCTTTCGTC

GAATTC-EcoRI

AAGCTT-HindIII

GCGC- HhaI

CCGAATC-Gal4 site

MRP2 cloned in pUC57 in EcoRV site (destroyed during cloning)

**Table S2: Identity and information about the 17 compounds which disrupts BLM-RAD54 interaction in protein complementation assay**

| Name of the compounds | EC50 (nM) | CAS Number | Structure |
|---|---|---|---|
| Azaguanine-8 (C1) | 22.34±1.975 | 134-58-7 | 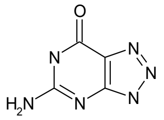 |
| Allantoin (C2) | 17.87±1.817 | 97-59-6 | 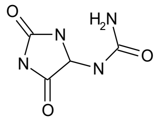 |
| Acetazolamide (C3) | 4.204±0.198 | 59-66-5 | 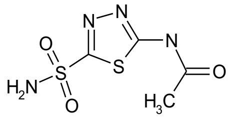 |
| Metformin hydrochloride (C4) | 3.013±0.8124 | 1115-70-4 | 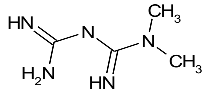 |
| Atracurum besylate (C5) | 5.376±2.1727 | 64228-81-5 | 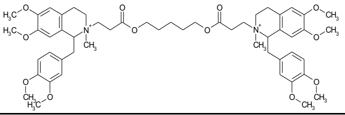 |
| Prednisone (C6) | 6.259±1.999 | 53-03-2 | 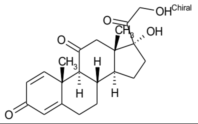 |
| Dipyridamole (C7) | 6.163±0.297 | 58-32-2 | 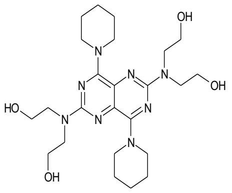 |
| Metronidazole (C8) | 20.51±2.606 | 443-48-1 | 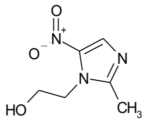 |
| Khellin (C9) | 16.66±1.263 | 82-02-0 | 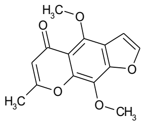 |
| R(-) Apomorphine hydrochloride hemihydrate (C10) | 5.195±1.335 | 41372-20-7 | 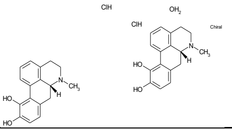 |
| Naloxone hydrochloride (C11) | 6.087±2.249 | 357-08-4 | 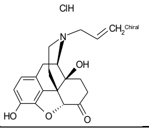 |
| Bromocryptine mesylate (C12) | 41.4±4.459 | 22260-51-1 | 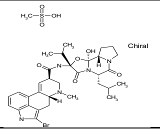 |

| | | | |
|---|---|---|---|
| Glipizide (C13) | 6.748±3.688 | 29094-61-9 | 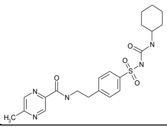 |
| Verapamil hydrochloride (C14) | 4.093±0.322 | 152-11-4 | 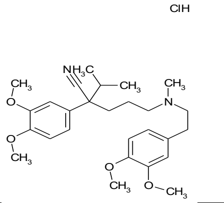 |
| Erythromycin (C15) | 8.119±3.888 | 114-07-8 | 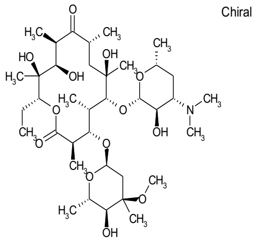 |
| Chloroxine (C16) | 26.41±2.279 | 773-76-2 | 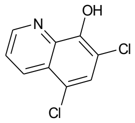 |
| Loxapine succinate (C17) | 22.92±4.247 | 27833-64-3 | 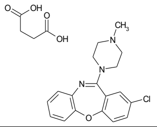 |

## Table S3: EC50 values of HCT116 WT and CPT and CDDP resistant HCT116 derivatives upon treatment with either C3, C7 or C17

**A. Effect of C3 on HCT116 WT and HCT116 CPT$^R$**

| | HCT116 WT | HCT116 WT + C3 | HCT116 WT CPT$^R$ | HCT116 WT CPT$^R$ + C3 |
|---|---|---|---|---|
| **EC50 (µM)** | 42.99±3.697 | 34.14±4.112 | 354.3±5.614 | 32.91±2.794 |
| **p value (t-test)** | 0.3179 | | 0.0005 | |

**B. Effect of C7 on HCT116 WT and HCT116 CPT$^R$**

| | HCT116 WT | HCT116 WT + C7 | HCT116 WT CPT$^R$ | HCT116 WT CPT$^R$ + C7 |
|---|---|---|---|---|
| **EC50 (µM)** | 42.99±3.697 | 19.5 | 354.3±5.614 | 38.762 |
| **p value (t-test)** | 0.0597 | | 0.00018 | |

**C. Effect of C17 on HCT116 WT and HCT116 CPT$^R$**

| | HCT116 WT | HCT116 WT + C17 | HCT116 WT CPT$^R$ | HCT116 WT CPT$^R$ + C17 |
|---|---|---|---|---|
| **EC50 (µM)** | 42.99±3.697 | 32.03 | 354.3±5.614 | 47.41 |
| **p value (t-test)** | 0.06412 | | 0.009342 | |

**D. Effect of C3 on HCT116 WT and HCT116 CDDP$^R$**

| | HCT116 WT | HCT116 WT + C3 | HCT116 WT CDDP$^R$ | HCT116 WT CDDP$^R$ + C3 |
|---|---|---|---|---|
| **EC50 (µM)** | 3.78 | 3.008 | 21.72 | 4.137 |
| **p value (t-test)** | 0.9854 | | 0.03541 | |

**E. Effect of C7 on HCT116 WT and HCT116 CDDP$^R$**

| | HCT116 WT | HCT116 WT + C7 | HCT116 WT CDDP$^R$ | HCT116 WT CDDP$^R$ + C7 |
|---|---|---|---|---|
| **EC50 (µM)** | 3.78 | 1.662 | 21.72 | 0.7216 |
| **p value (t-test)** | 0.05323 | | 0.00183 | |

**E. Effect of C17 on HCT116 WT and HCT116 CDDP$^R$**

| | HCT116 WT | HCT116 WT + C17 | HCT116 WT CDDP$^R$ | HCT116 WT CDDP$^R$ + C17 |
|---|---|---|---|---|
| **EC50 (µM)** | 3.78 | 2.81 | 21.72 | 3.78 |
| **p value (t-test)** | 0.0786 | | 0.00439 | |

## Table S4: HCT116 WT and HCT116 1-OHP$^{R}$ cells upon treatment with C3, C7 or C17

| | **HCT116 WT + 1-OHP** | **HCT116 WT + 1-OHP + C3** | **HCT116 WT + 1-OHP + C7** | **HCT116 WT + 1-OHP + C17** | **HCT116 1-OHP$^{R}$ + 1-OHP** | **HCT116 1-OHP$^{R}$ + 1-OHP + C3** | **HCT116 1-OHP$^{R}$ + 1-OHP + C7** | **HCT116 1-OHP$^{R}$ + 1-OHP + C17** |
|---|---|---|---|---|---|---|---|---|
| **EC50 (µM)** | 4.769± 0.0367 | 3.716± 0.0775 | 4.935± 0.03757 | 3.135± 0.0337 | 14.1± 1.402 | 7.06± 2.66 | 15.1± 1.593 | 5.172± 1.526 |
| **p-value (t-test)** | | 0.0586 | 0.0722 | 0.0714 | | 0.0247 | 0.0055 | 0.0251 |

**Table S5: List of antibodies used in the study**

| Name of antibody | Source | Identifier (Dilution used) |
|---|---|---|
| Anti-BLM (used for WB human samples, IP, IF, PLA, ChIP, ChIP-seq) | Bethyl Laboratories | Cat# A300-110A; RRID:AB_2064794 (WB: 1:2500, IP: 1μg/reaction, ChIP: 1μg/reaction, PLA 1:150) |
| Anti-BLM (used for WB human samples) | Novus Biologicals | Cat# NB 100-214; RRID:AB_350136 (WB: 1:1000) |
| Anti-BLM (used for WB murine samples) | Novus Biologicals | Cat# NB 100-1588; RRID:AB_10001235 (WB: 1:1000) |
| Anti-RAD54 (used for WB human samples, IF, ChIP) | Abcam | Cat# ab10705; RRID:AB_297417 (WB: 1:2000, IF: 1:200, ChIP: 1μg/reaction) |
| Anti-RAD54 (used for WB murine and human samples, IP, PLA) | Santa Cruz Biotechnology | Cat# sc-374598; RRID:AB_10989787 (WB: 1:1000, IP: 1μg/reaction, PLA 1:150) |
| Anti-RAD51 (used for WB, IF) | Santa Cruz Biotechnology | Cat# sc-8349; RRID: AB_2253533 (WB: 1:1000; IF: 1:200) |
| Anti-hsp90 (used for WB) | Santa Cruz Biotechnology | Cat# sc-7947; RRID: AB_2121235 (WB: 1:2000) |
| Anti-PCNA (used for WB) | Santa Cruz Biotechnology | Cat# sc-56; RRID:AB_628110 (WB: 1:1000) |
| Anti-Ki67 (used for IHC) | Abcam | Cat# ab16667; RRID: AB_302459 (IHC: 1:100) |
| Anti-p21 (used for WB) | Santa Cruz Biotechnology | Cat# sc-397; RRID: AB_632126 (WB: 1:2000) |
| Anti-p27 (used for WB) | Santa Cruz Biotechnology | Cat# sc-528; RRID: AB_632129 (WB: 1:2000) |
| Anti-βactin (used for WB) | Santa Cruz Biotechnology | Cat# sc-47778; RRID: AB_2714189 (WB: 1:2000) |
| Anti-γH2AX (used for WB, IF) | Abcam | Cat# ab26350; RRID: AB_470861 (WB: 1:2000, IF: 1:250) |
| Anti-GFP (used for WB) | Santa Cruz Biotechnology | Cat# sc-8334; RRID: AB_641123 (WB: 1:2000) |
| Anti-GFP (Living Colors) (used for WB, IP) | Clontech/Takara | Cat#: 632460; RRID: AB_2314544 (WB: 1:2000, IP: 1μg/reaction) |
| Anti-His probe (used for WB) | Santa Cruz Biotechnology | Cat# sc-8036; AB_627727 (WB: 1:2000) |
| Anti-GST tag (used for WB) | Thermo Fisher Scientific | Cat# 71-7500; RRID:AB_2533994 WB: 1:4000) |
| Anti-MRP2/ABCC2 (used for WB) | Cell Signalling | Cat# 12559; RRID: AB_2797954 (WB: 1:1000) |
| Anti-MRP3/ABCC3 | Cell Signalling | Cat# 39909; RRID: AB_2799164 |

| | | |
|---|---|---|
| (used for WB) | | (WB: 1:1000) |
| Anti-MRP5<br>(used for WB) | Santa Cruz Biotechnology | Cat# sc-376965;<br>(WB: 1:1000) |
| Rabbit Control IgG | Abcam | Cat# ab46540; RRID: AB_2614925<br>(IP: 1μg/reaction) |

WB: Western blotting
IF: Immunofluorescence
IHC: Immunohistochemistry
IP: Immunoprecipitation
ChIP: Chromatin Immunoprecipitation
ChIP-seq: Chromatin Immunoprecipitation followed by sequencing
PLA: Proximity Ligation Assay

**Table S6: List of recombinant DNAs used in the study**

| Name of the recombinant DNA | Source | Identifier |
|---|---|---|
| p2085S-G5E4 | Gifted by Jerry Workman (Stowers Institute for Medical Research, Kansas City, USA) | (17) |
| pET28b-RAD54 (1-747) (referred as His RAD54 WT) | This study | N/A |
| pGEX4T-1 RAD54 (1-747) (referred as GST RAD54 WT) | Present in the lab of corresponding author | (10) |
| pGEX4T-1 BLM (1-1417) (referred as GST BLM WT) | Present in the lab of corresponding author | (10) |
| pGEX4T-1 BLM (1-212) | Gifted by Ian Hickson (University of Copenhagen, Copenhagen, Denmark) | (18) |
| HR plasmid | Gifted by Vera Gorbunova (University of Rochester, New York, USA) | (19) |
| Td-Tomato-N1 | Addgene (Cat# 54642) | (20) |
| pEGFP-C1 BLM (1-1417) (referred as GFP BLM) | Present in the lab of corresponding author | (21) |
| pLVX AcGFP N1 BLM (Δ181-212) [referred as GFP BLM (Δ181-212)] | This study | N/A |
| pEGFP-C1 NLS BLM (181-212) | This study | N/A |
| p3XFlag-Myc-CMV24 BLM (181-212) | This study | N/A |
| pcDNA3.1 N-RAD54 (1-212) *R*luc-F(1) (referred as N-RAD54-F1) | This study | N/A |
| pcDNA3.1 C-RAD54 (213-747) *R*luc-F(2) (referred as C-RAD54-F1) | This study | N/A |
| pcDNA3.1 BLM (181-212) *R*luc-F(1) (referred BLM-F1) | This study | N/A |
| pcDNA3.1 BLM (181-212) *R*luc-F(2) (referred BLM-F2) | This study | N/A |
| pEGFP-C1-NLS BLM (181-212) | This study | N/A |
| pUC57-MRP2 | This study | N/A |
| pDonor | Addgene (Cat# 130634) | (22) |
| PHASE-DEST-CAS9-P2A-GFP | This study | N/A |
| pMDLg/pRRE | Addgene (Cat# 12251) | (23) |
| pRSV/REV | Addgene (Cat# 12253) | (23) |
| psPAX2 | Addgene (Cat# 12260) | N/A |

| | | |
|---|---|---|
| | (Gifted by Didier Trono, University of Geneva, Geneva, Switzerland) | |
| pMD2.G | Addgene (Cat# #12259) (Gifted by Didier Trono, University of Geneva, Geneva, Switzerland | N/A |
| mCherry2-C1 | Addgene (Cat# 54563) (Gifted by Michael Davidson, Florida State University, Tallahassee, USA) | N/A |
| CRISPR Double cut donor vector for BLM for correction of C>A at nucleotide 1784 (exon 7) in the BLM gene | This study | N/A |
| pLVX AcGFP N1 (referred as GFP vector) | Present in the lab of corresponding author | N/A |

N/A: Not applicable

**Table S7: List of reagents used in the study**

| Name | Source | Identifier |
|---|---|---|
| **Chemicals** | | |
| Prestwick Chemical Library | Prestwick Chemical Libraries | N/A |
| IPTG | Merck | Cat# I6758; CAS Number 367-93-1 |
| PMSF | Merck | Cat# P7626; CAS Number 329-98-6 |
| DTT | Merck | Cat# D0632; CAS Number 3483-12-3 |
| Triton-X-100 | Merck | Cat# T9284; CAS Number 9002-93-1 |
| Hydroxyurea | Merck | Cat# H8627; CAS Number 127-07-1 |
| Coelenterazine h | MediLumine Inc | Cat#304CH, CAS Number 50909-86-9 |
| ATP | Merck | Cat# A9187; CAS Number 74804-12-9 |
| (*S*)-(+)-Camptothecin | Merck | Cat# C9911; CAS Number 7689-03-4 |
| (*S*)-(+)-Camptothecin | J&H Chemical Co., Ltd | Custom Synthesis, CAS Number 7689-03-4 |
| Cisplatin | Merck | Cat# P4394; CAS Number 15663-27-1 |
| Oxaliplatin | Merck | Cat# O9512; CAS Number 61825-94-3 |
| Acetazolamide | Merck | Cat# A6011; CAS Number 59-66-5 |
| Dipyridamole | Merck | Cat# D9766; CAS Number 58-32-2 |
| Loxapine succinate | Merck | Cat# L106; CAS Number 27833-64-3 |
| G418 Sulfate | Merck | Cat# A1720; CAS Number 108321-42-2 |
| DMSO | Merck | Cat# D2650; CAS Number 67-68-5 |
| Lithium chloride | Merck | Cat# L4408; CAS Number 7447-41-8 |
| Sodium deoxycholate | Merck | Cat# D6750; CAS Number 302-95-4 |
| Imidazole | Bio Basic | Cat# IB0277; CAS Number 288-32-4 |
| Proteinase K | Bio Basic | Cat# PB0451; CAS Number 39450-01-6 |
| L-Glutathione reduced | Merck | Cat# G6529; CAS Number 70-18-8 |
| Thiazolyl Blue Tetrazolium Bromide (MTT) | Merck | Cat# M2128; CAS Number 298-93-1 |
| Crystal Violet | Merck | Cat# C0775; CAS Number 548-62-9 |
| L-α-Phosphatidylcholine | Merck | Cat# P3556; CAS Number 8002-43-5 |
| Cholesterol | Merck | Cat# C8667; CAS Number 57-88-5 |
| 1,2-distearoyl-sn-glycero-3-phosphoethanolamine-N-[amino(polyethyleneglycol)-2000] ammonium salt (DSPE-PEG2000-Amine) | Avanti Polar Lipids, Inc. | Cat# 880128; CAS Number 474922-26-4 |
| 5(6)-Carboxy-2′,7′-dichlorofluorescein | Merck | Cat#21882; CAS Number 111843-78-8 |
| **Recombinant proteins** | | |
| GST RAD54 | This study | N/A |
| His RAD51 | This study | N/A |

| | | |
|---|---|---|
| GST BLM | This study | N/A |
| GST BLM (Δ181-212) | This study | N/A |
| GST BLM (1-212) | This study | N/A |
| **Cell Lines** | | |
| HEK293T | ATCC | Cat# CRL-3216 |
| Lenti-X 293T | Takara | Cat# 632180 |
| GM03509 GFP-BLM | Present in the lab of corresponding author | (24) |
| GM03509 GFP | Present in the lab of corresponding author | (24) |
| HCT116 WT | Gifted by Bert Vogelstein (Johns Hopkins Medicine, Baltimore, USA) | (25) |
| HCT116 BLM -/- | Gifted by Bert Vogelstein (Johns Hopkins Medicine, Baltimore, USA) | (25) |
| HCT116 BLM -/- + GFP | This study | N/A |
| HCT116 BLM -/- + GFP-BLM (181-212) | This study | N/A |
| HCT116 IC60 $CPT^R$ | This study | N/A |
| HCT116 (Δ181-212) $CPT^R$ | This study | N/A |
| HCT116 IC60 $CDDP^R$ | This study | N/A |
| HCT116 1-$OHP^R$ | Gifted by Lee M Ellis (The University of Texas MD Anderson Cancer Center, Houston, USA) | (2) |
| HT-29 1-$OHP^R$ | Gifted by Lee M Ellis (The University of Texas MD Anderson Cancer Center, Houston, USA) | (2) |
| HCT116 1-$OHP^R$ | This study | N/A |
| CT26 | ATCC | Cat# CRL-2638 |
| DLD1 | Gifted by Bert Vogelstein (Johns Hopkins Medicine, Baltimore, USA) | (26) |
| HT-29 | National Cell Repository, NCCS, Pune, India | N/A |

| | | |
|---|---|---|
| SW480 | National Cell Repository, NCCS, Pune, India | N/A |
| SW620 | National Cell Repository, NCCS, Pune, India | N/A |
| HeLa | ATCC | Cat# CCL-2 |
| GM03509 BLM Clone 9.6 | This study | N/A |
| **Peptides** | | |
| BLM (181-212) peptide (referred as BLM_peptide) | Labex Corporation | N/A |
| Scrambled peptide (referred as SCM_peptide) | Labex Corporation | N/A |
| TAMRA conjugated BLM (181-212) Cell Permeable Peptide (referred as BLM_CPP) | Anaspec Inc. | N/A |
| TAMRA conjugated Scrambled Cell Permeable Peptide (referred as SCM_CPP) | Anaspec Inc. | N/A |
| Biotinylated BLM (181-212) Peptide (referred as Biotin BLM_Peptide | Labex Corporation | N/A |
| **Oligonucleotides** | | |
| siRNA sequences for BLM | Dharmacon | (27) |
| siRNA sequences for RAD54 | Dharmacon | (28) |
| siRNA sequences for MRP2 | Dharmacon | (29) |
| ON-TARGETplus Non-targeting siRNA #1 | Dharmacon | Cat# D-001810-01-05 |
| **Other** | | |
| Fetal bovine Serum | Thermo Fisher Scientific | Cat# 10082147 |
| Advanced DMEM | Thermo Fisher Scientific | Cat# 12491-023 |

| | | |
|---|---|---|
| McCoy's 5A Medium | Thermo Fisher Scientific | Cat# 16600-108 |
| Optimem reduced serum medium | Thermo Fisher Scientific | Cat# 31985-088 |
| Qubit® dsDNA HS Assay Kit | Thermo Fisher Scientific | Cat# Q32851 |
| InsTAclone PCR Cloning Kit | Thermo Fisher Scientific | Cat# K1213 |
| Xfect transfection reagent | Takara | Cat# 631317 |
| Surveyor Mutation Detection Kit | Integrated DNA Technologies | Cat# 706020 |
| NEBNext Ultra II DNA Library Prep with Sample Purification Beads | New England Biolabs | Cat# E7103L |
| LentiX concentrator | Takara | Cat# 631232 |
| [$\gamma^{32}$P] ATP | Perkin Elmer | Cat# BLU002Z250UC |
| [$\alpha^{32}$P] dATP | Perkin Elmer | Cat# NEG012H250UC |
| Lipofectamine 2000 | Thermo Fisher Scientific | Cat# 11668019 |
| FuGENE® HD Transfection Reagent | Promega | Cat# E2311 |
| Complete Protease Cocktail inhibitor | Roche | Cat# 11697498001 |
| BL21 (DE3) competent cells | Thermo Fisher Scientific | Cat# EC0114 |
| Poly-Prep Chromatography column | Biorad | Cat# 73101550 |
| QIAamp DNA Mini Kit | Qiagen | Cat#51304 |
| Mounting medium with DAPI | Vector Laboratories | Cat# H-1200 |
| T7 Quick coupled Transcription/ Translation system | Promega Corporation | Cat# L1170 |
| Fluorometric TUNEL System | Promega Corporation | Cat# G3250 |
| Asp718 | Roche | Cat# 10814253001 |
| ClaI | Roche | Cat# 10656291001 |
| HhaI | New England BioLabs | Cat# R0139L |
| I-SceI | New England BioLabs | Cat# R0694S |
| EcoR1 | New England BioLabs | Cat# R0101L |
| HindIII | New England BioLabs | Cat# R0104L |

| | | |
|---|---|---|
| ΦX174 RFI DNA | New England BioLabs | Cat# N3021L |
| T4 Polynucleotide Kinase | New England BioLabs | Cat# M0201L |
| EpiMark® Nucleosome Assembly Kit | New England BioLabs | Cat# E5350S |
| Glutathione S-Sepharose | Cytiva | Cat# 17-5279-02 |
| His-Select Nickel Affinity Gel | Merck | Cat# P6611 |
| Phenol:Chloroform:Isoamyl Alcohol 25:24:1 Saturated with 10 mM Tris, pH 8.0, 1 mM EDTA | Merck | Cat# P3803 |
| cOmplete™ Protease Inhibitor Cocktail | Merck | Cat# 11697498001 |
| TLC PEI Cellulose F | Merck | Cat# 1.05579.0001 |
| P81 phosphocellulose paper | Merck | Cat# Z753645 |
| Octet® Ni-NTA (NTA) Biosensors | Sartorius | Cat# 18-5101 |
| TRIzol Reagent | Thermo Fisher Scientific | Cat# 15596026 |
| Reverse transcriptase core kit | Eurogentec | Cat# RT-RTCK-05 |
| Duolink® In Situ Red Starter Kit Mouse/Rabbit | Merck | Cat# DUO92101 |
| **Animals** | | |
| NOD SCID mice (male or female, 6-8 weeks) | The Jackson Laboratory | Stock# 001303 |
| NSG mice (male or female, 6-8 weeks) | The Jackson Laboratory | Stock# 005557 |
| BALB/c (male or female, 6-8 weeks) | The Jackson Laboratory | Stock# 000651 |
| **Deposited Data** | | |
| Raw sequencing reads for BLM ChIP-seq | ArrayExpress | Accession E-MTAB-11372. |
| BLM ChIP-seq data analyzed (chromosomal location and TSS) | | |

N/A: Not applicable

**Table S8: Primers used in the present study**

| RT-PCR primers | | |
|---|---|---|
| **Gene Name** | **Forward Primer Sequence (5'-3')** | **Reverse Primer Sequence (5'-3')** |
| MRP1 | AAACCATCCACGACCCTAATC | CCTCCTTGTTTAAGGACCAGAG |
| MXR | TTCAGGTCTGTTGGTCAATCTC | TTATGCTGCAAAGCCGTAAATC |
| MDR1 | GGCCTAATGCCGAACACATT | CAGCGTCTGGCCCTTCTTC |
| MRP2 (human) | GAACACTTAGCCGCAGTTCTA | TTCGTCTTCCTTCAGGCTATTC |
| MRP3 (human) | AAAAGCAGACGGCACGACA | GCAGGCACTGATGAGGAAGC |
| MRP4 (human) | AAGTGAACAACCTCCAGTTCCAG | GGCTCTCCAGAGCACCATCT |
| MRP5 (human) | CGTGAACTGCAGAAGACTAGAG | CACACGATGGACAGGATGAG |
| MRP2 (murine) | TACCAGCGAGTTATCGAAGCGTG | TGCTTCTGACCGCCACTGAGAT |
| MRP3 (murine) | ACTTCCTCCGAAACTACGCACC | GCTGGCTCATTGTCTGTCAGGT |
| MRP5 (murine) | GCAAACTGGTTGGAATCTGCGG | CAAAGGTCCCACTGACGGCAAT |
| ABCA2 | ACACCTCTGGTTCTACTCACGG | CCGACAATGTCTGCACCAGTGA |
| BSEP | GCTCCAAGTCTCAGCTTTCTTA | GGAATGTCCTTGTCCTTTCTATCT |
| ABCB5 | TTTGCCTATGCGGCAGGGTTTC | CAAAACGAGCGTTTCTCCGATGG |
| BLM | AGACAGGATTCTCTGCCACCAGG A | TGGTGTTTCAGCCCAGTTGCT |
| RAD54 | ATGAGGTTGGGAAATGGC | GAGGACTCCAACATGAAG |
| CTTN (control) | GCCGACCGAGTAGACAAG | GTATTTGCCGCCGAAACC |
| β-Actin (control) (murine) | CAGCCTTCCTTCTTGGGTATG | GGCATAGAGGTCTTTACGGATG |
| **ChIP-qPCR primers** | | |
| **Target Promoter s** | **Forward Primer Sequence (5'-3')** | **Reverse Primer Sequence (5'-3')** |
| MRP2 | ATACCGCATGGGTGGTTC | CTTCAACAATCCTTCTGCCTTG |
| MRP3 | CGGACTTGTTCGGGAGTG | GTCTCTCTAAATACCGAGGCTAAC |
| MDR1 | CAATTGCTGTCTTGAAGGATATA CA | GTGACATTCCTTATTAGTTGAATTGG TC |
| GAPDH (control) | GCAGCCCCTTCATACCCTCAC GT | GAGCCACACCATCCTAGTTGC |

**Supplemental References**


1. Stefan E, Aquin S, Berger N, Landry CR, Nyfeler B, Bouvier M, et al. Quantification of dynamic protein complexes using Renilla luciferase fragment complementation applied to protein kinase A activities in vivo. *Proc Natl Acad Sci U S A.* 2007;104(43):16916-21.
2. Yang AD, Fan F, Camp ER, van Buren G, Liu W, Somcio R, et al. Chronic oxaliplatin resistance induces epithelial-to-mesenchymal transition in colorectal cancer cell lines. *Clinical cancer research : an official journal of the American Association for Cancer Research.* 2006;12(14 Pt 1):4147-53.
3. Priyadarshini R, Hussain M, Attri P, Kaur E, Tripathi V, Priya S, et al. BLM Potentiates c-Jun Degradation and Alters Its Function as an Oncogenic Transcription Factor. *Cell reports.* 2018;24(4):947-61.e7.
4. Schmidt D, Wilson MD, Spyrou C, Brown GD, Hadfield J, and Odom DT. ChIP-seq: using high-throughput sequencing to discover protein-DNA interactions. *Methods.* 2009;48(3):240-8.
5. Furlan-Magaril M, Rincon-Arano H, and Recillas-Targa F. Sequential chromatin immunoprecipitation protocol: ChIP-reChIP. *Methods in molecular biology.* 2009;543:253-66.
6. Bolger AM, Lohse M, and Usadel B. Trimmomatic: a flexible trimmer for Illumina sequence data. *Bioinformatics.* 2014;30(15):2114-20.
7. Langmead B, Trapnell C, Pop M, and Salzberg SL. Ultrafast and memory-efficient alignment of short DNA sequences to the human genome. *Genome Biol.* 2009;10(3):R25.
8. Bardet AF, He Q, Zeitlinger J, and Stark A. A computational pipeline for comparative ChIP-seq analyses. *Nat Protoc.* 2011;7(1):45-61.
9. Zhang JP, Li XL, Li GH, Chen W, Arakaki C, Botimer GD, et al. Efficient precise knockin with a double cut HDR donor after CRISPR/Cas9-mediated double-stranded DNA cleavage. *Genome Biol.* 2017;18(1):35.
10. Srivastava V, Modi P, Tripathi V, Mudgal R, De S, and Sengupta S. BLM helicase stimulates the ATPase and chromatin-remodeling activities of RAD54. *Journal of cell science.* 2009;122(Pt 17):3093-103.

11. Olive PL, and Banath JP. The comet assay: a method to measure DNA damage in individual cells. *Nat Protoc.* 2006;1(1):23-9.
12. Seluanov A, Mao Z, and Gorbunova V. Analysis of DNA double-strand break (DSB) repair in mammalian cells. *J Vis Exp.* 2010(43).
13. Chabosseau P, Buhagiar-Labarchede G, Onclercq-Delic R, Lambert S, Debatisse M, Brison O, et al. Pyrimidine pool imbalance induced by BLM helicase deficiency contributes to genetic instability in Bloom syndrome. *Nature communications.* 2011;2:368.
14. Pal S, Medatwal N, Kumar S, Kar A, Komalla V, Yavvari PS, et al. A Localized Chimeric Hydrogel Therapy Combats Tumor Progression through Alteration of Sphingolipid Metabolism. *ACS Cent Sci.* 2019;5(10):1648-62.
15. Vellonen KS, Mannermaa E, Turner H, Hakli M, Wolosin JM, Tervo T, et al. Effluxing ABC transporters in human corneal epithelium. *J Pharm Sci.* 2010;99(2):1087-98.
16. Tripathi V, Nagarjuna T, and Sengupta S. BLM helicase-dependent and -independent roles of 53BP1 during replication stress-mediated homologous recombination. *J Cell Biol.* 2007;178(1):9-14.
17. Neely KE, Hassan AH, Wallberg AE, Steger DJ, Cairns BR, Wright AP, et al. Activation domain-mediated targeting of the SWI/SNF complex to promoters stimulates transcription from nucleosome arrays. *Mol Cell.* 1999;4(4):649-55.
18. Wu L, Davies SL, North PS, Goulaouic H, Riou JF, Turley H, et al. The Bloom's syndrome gene product interacts with topoisomerase III. *J Biol Chem.* 2000;275(13):9636-44.
19. Mao Z, Bozzella M, Seluanov A, and Gorbunova V. DNA repair by nonhomologous end joining and homologous recombination during cell cycle in human cells. *Cell Cycle.* 2008;7(18):2902-6.
20. Shaner NC, Campbell RE, Steinbach PA, Giepmans BN, Palmer AE, and Tsien RY. Improved monomeric red, orange and yellow fluorescent proteins derived from Discosoma sp. red fluorescent protein. *Nat Biotechnol.* 2004;22(12):1567-72.
21. Hu P, Beresten SF, van Brabant AJ, Ye TZ, Pandolfi PP, Johnson FB, et al. Evidence for BLM and Topoisomerase IIIalpha interaction in genomic stability. *Human molecular genetics.* 2001;10(12):1287-98.

22. Klompe SE, Vo PLH, Halpin-Healy TS, and Sternberg SH. Transposon-encoded CRISPR-Cas systems direct RNA-guided DNA integration. *Nature.* 2019;571(7764):219-25.
23. Dull T, Zufferey R, Kelly M, Mandel RJ, Nguyen M, Trono D, et al. A third-generation lentivirus vector with a conditional packaging system. *J Virol.* 1998;72(11):8463-71.
24. Kharat SS, Tripathi V, Damodaran AP, Priyadarshini R, Chandra S, Tikoo S, et al. Mitotic phosphorylation of Bloom helicase at Thr182 is required for its proteasomal degradation and maintenance of chromosomal stability. *Oncogene.* 2016;35(8):1025-38.
25. Traverso G, Bettegowda C, Kraus J, Speicher MR, Kinzler KW, Vogelstein B, et al. Hyper-recombination and genetic instability in BLM-deficient epithelial cells. *Cancer Res.* 2003;63(24):8578-81.
26. Rajagopalan H, Jallepalli PV, Rago C, Velculescu VE, Kinzler KW, Vogelstein B, et al. Inactivation of hCDC4 can cause chromosomal instability. *Nature.* 2004;428(6978):77-81.
27. Tikoo S, Madhavan V, Hussain M, Miller ES, Arora P, Zlatanou A, et al. Ubiquitin-dependent recruitment of the Bloom Syndrome helicase upon replication stress is required to suppress homologous recombination. *EMBO J.* 2013;32(12):1778-92.
28. Spies J, Waizenegger A, Barton O, Surder M, Wright WD, Heyer WD, et al. Nek1 Regulates Rad54 to Orchestrate Homologous Recombination and Replication Fork Stability. *Mol Cell.* 2016;62(6):903-17.
29. Materna V, Stege A, Surowiak P, Priebsch A, and Lage H. RNA interference-triggered reversal of ABCC2-dependent cisplatin resistance in human cancer cells. *Biochem Biophys Res Commun.* 2006;348(1):153-7.
