# Supplementary material for: Small molecules that disrupt RAD54-BLM interaction hamper tumor proliferation in colon cancer chemoresistance models
Source: J Clin Invest. 2024 Feb 29;134(8):e161941. doi: 10.1172/JCI161941 (PMC11014671; doi:10.1172/JCI161941)

Figure 1B
(Lysates)

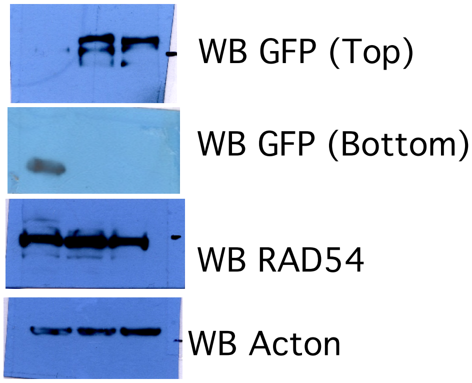

Figure 1B
(Immunoprecipitation)

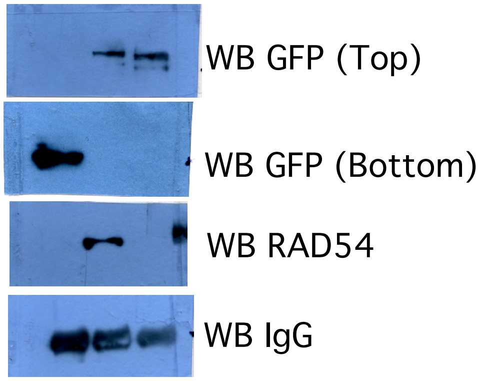

Figure 1C
(Lysates)

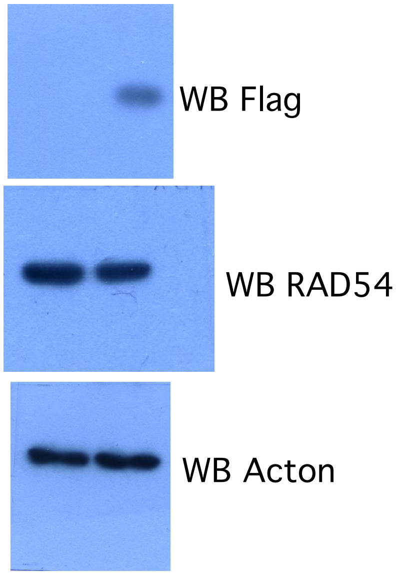

Figure 1C
(Immunoprecipitation)

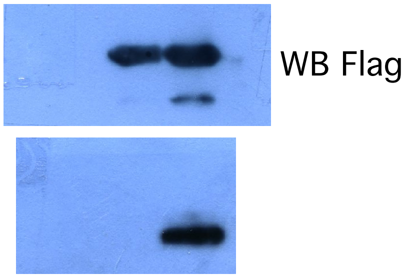

Figure 1D

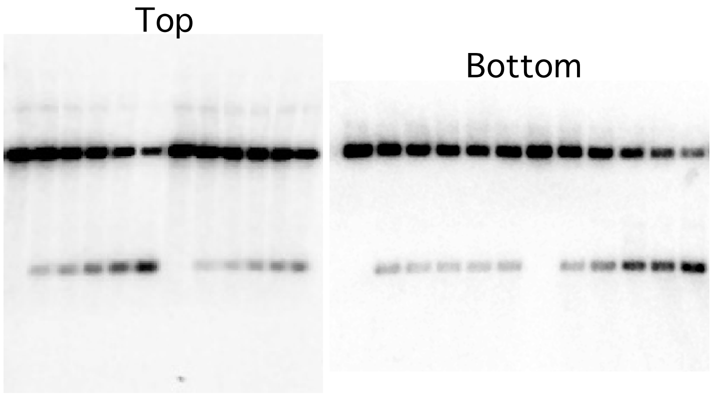

Autoradiogram

Unedited Data Figure 2
Kaur et al.,

Figure 2B

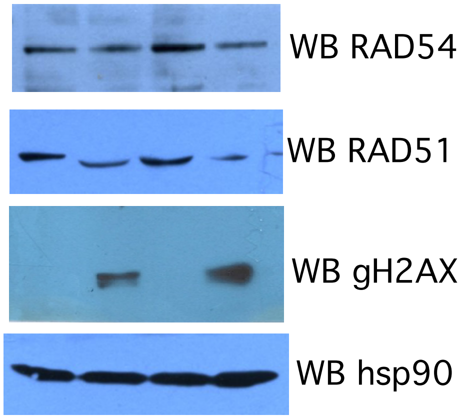

Figure 2C

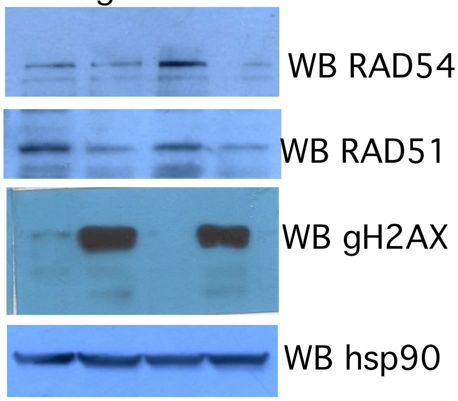

Figure 2F

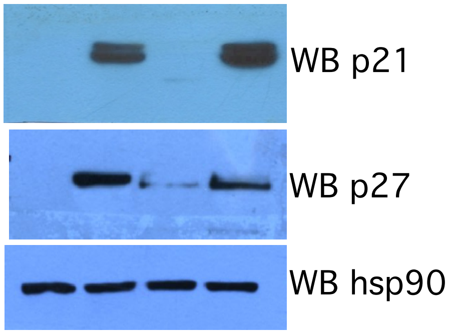

Figure 2G

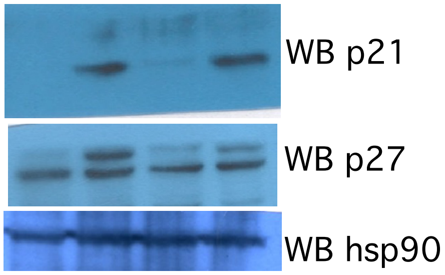

Figure 3D

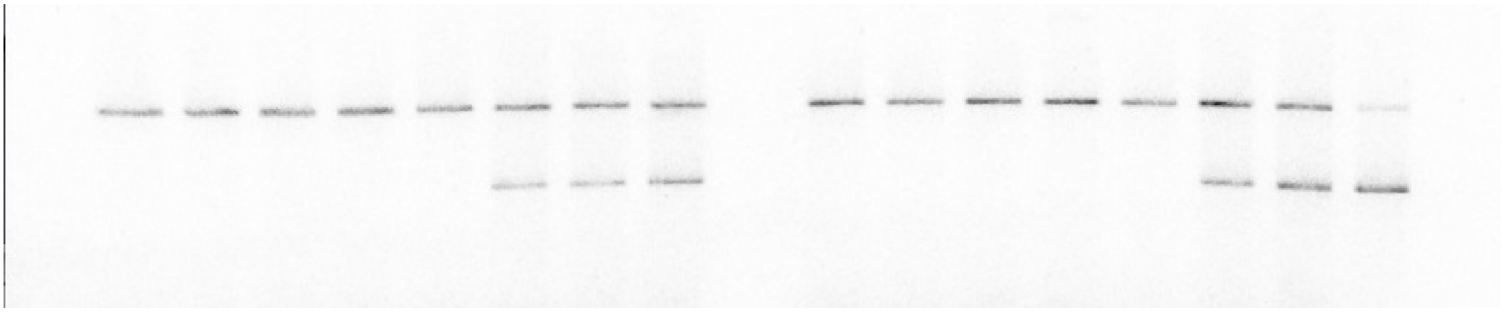

Autoradiogram

Figure 4C

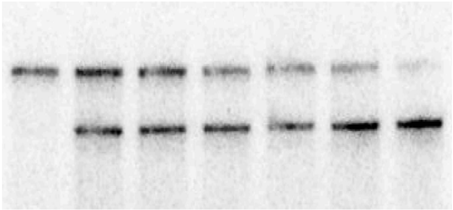

Left

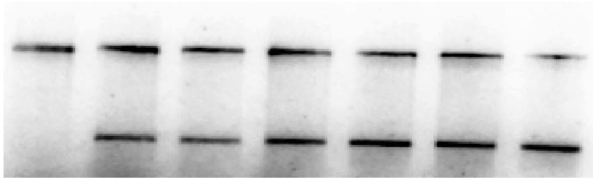

Middle

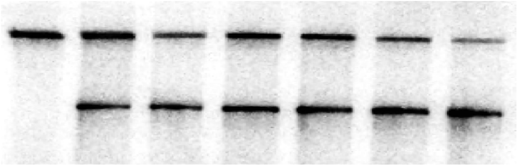

Right

Autoradiogram

Figure 5G

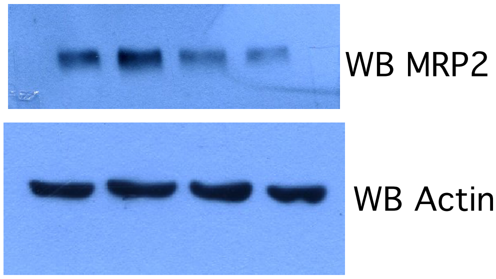

WB MRP2

WB Actin

Figure 6I

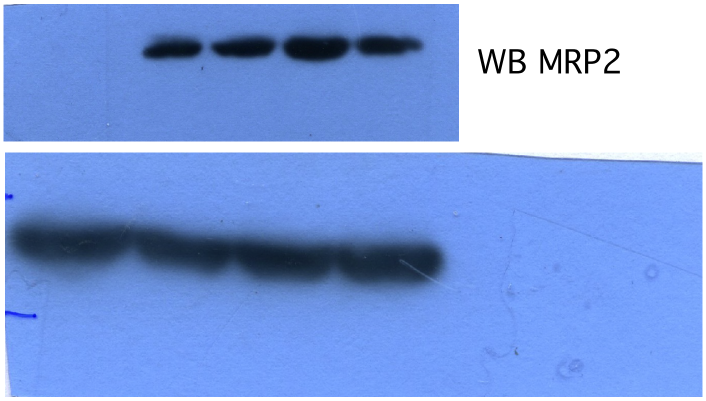

Unedited Data Figure S1
Kaur et al.,

Figure S1A

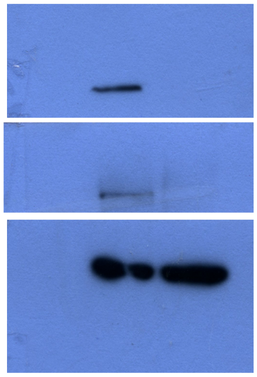

WB RAD54

WB BLM

WB Actin

Figure S1D
(Lysates)

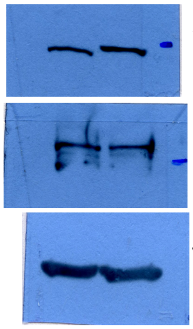

WB RAD54

WB BLM

WB Actin

Figure S1D
(Immunoprecipitation)

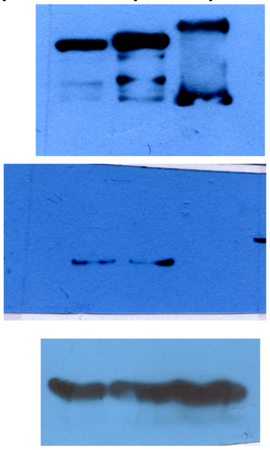

WB RAD54

WB BLM

WB IgG

Figure S1E

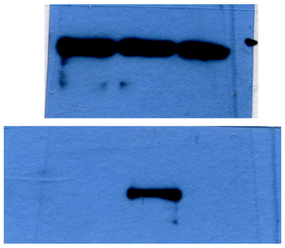

WB His

WB GST

Figure S1F

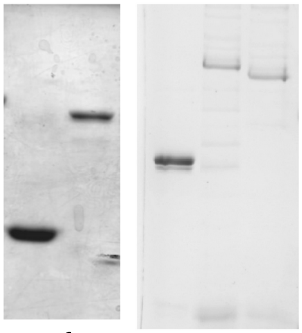

Left

Right

Figure S1G

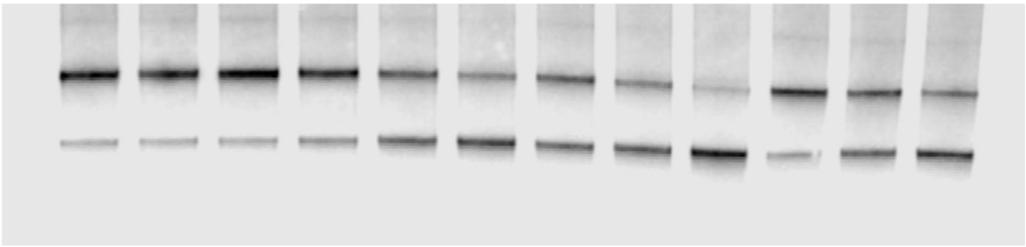

Autoradiography

Figure S2A

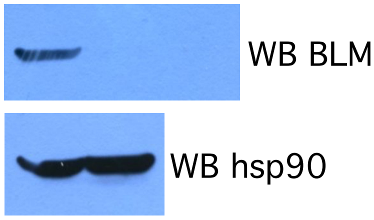

WB BLM

WB hsp90

Figure S2E

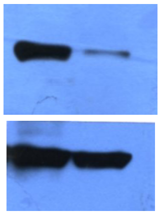

WB BLM

WB hsp90

Figure S2G

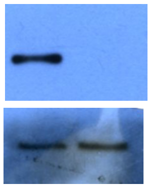

WB BLM

WB hsp90

Figure S2I

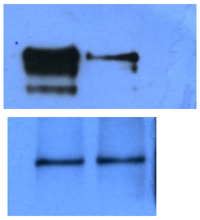

WB BLM

WB hsp90

Figure S2K

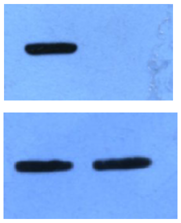

WB BLM

WB hsp90

Figure S3B

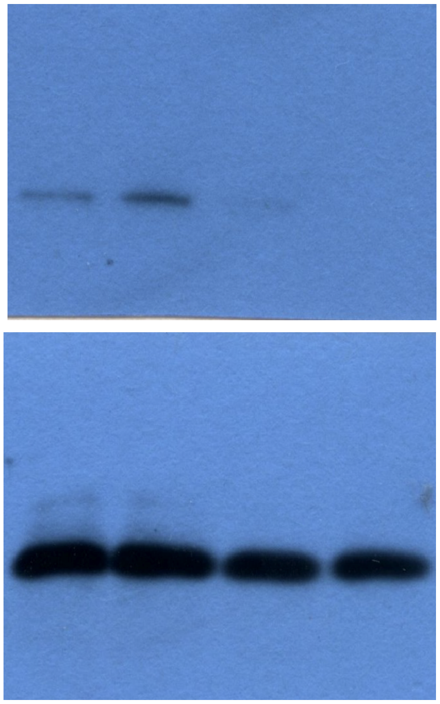

Figure S4B

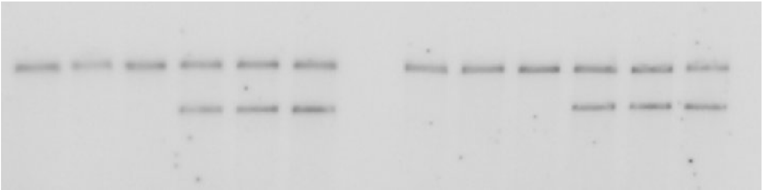

Autoradiography

Figure S4D

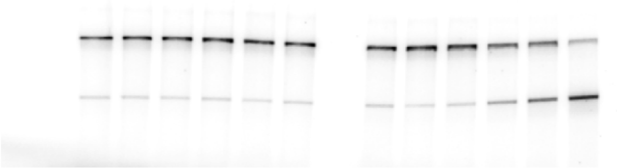

Autoradiography

Unedited Data Figure S5
Kaur et al.,

Figure S5B
(Lysates)

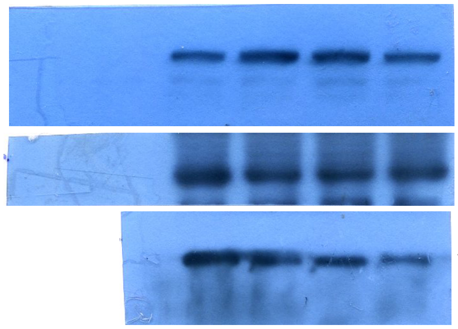

Figure S5B
(Immunoprecipitation)

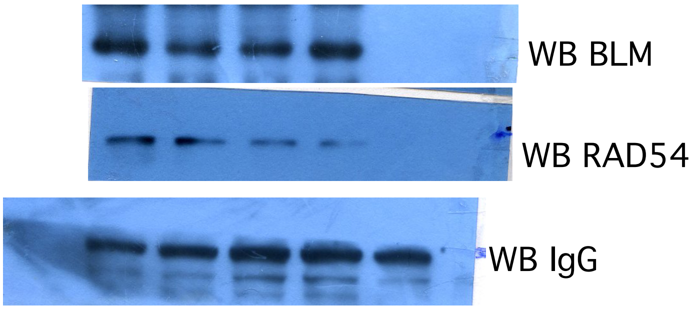

Figure S5C

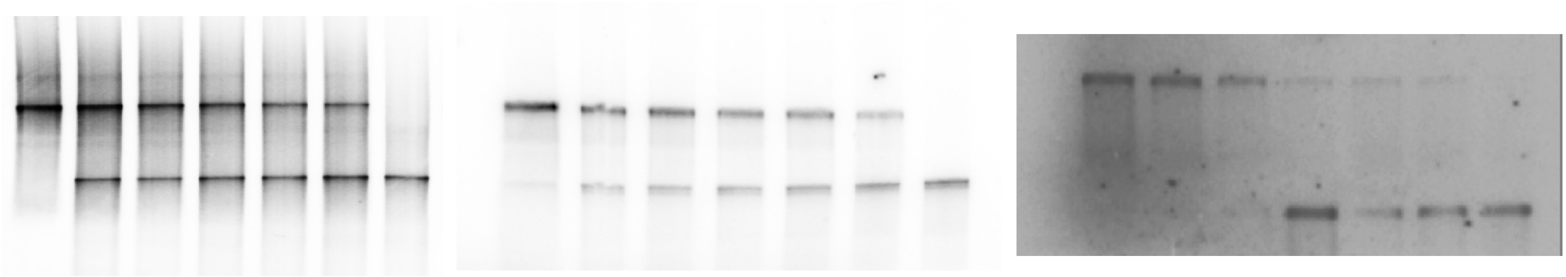

Autoradiography

Figure S6D
(Lysates)

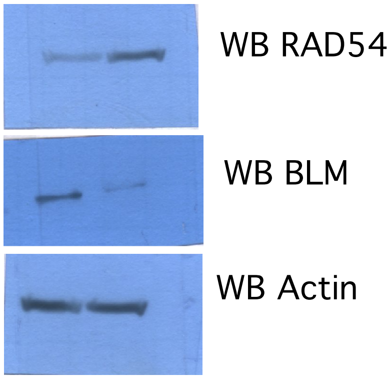

Figure S6D
(Immunoprecipitation)

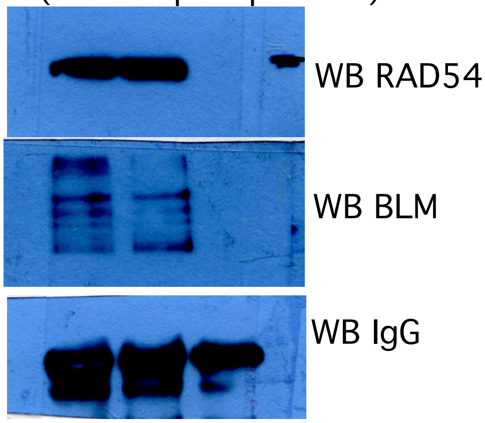

Figure S7E

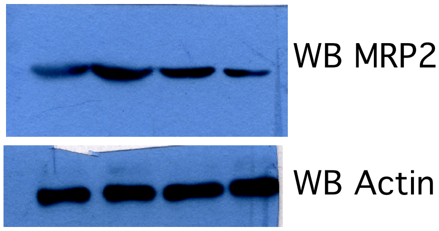

Figure S7G

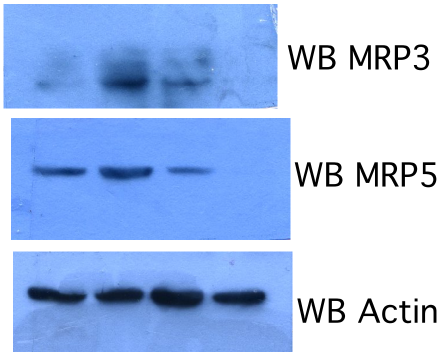

Figure S8E

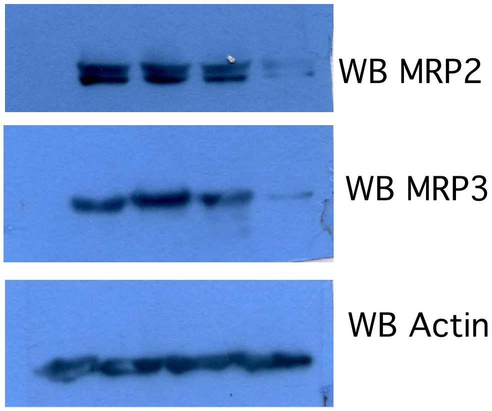

Figure S9C

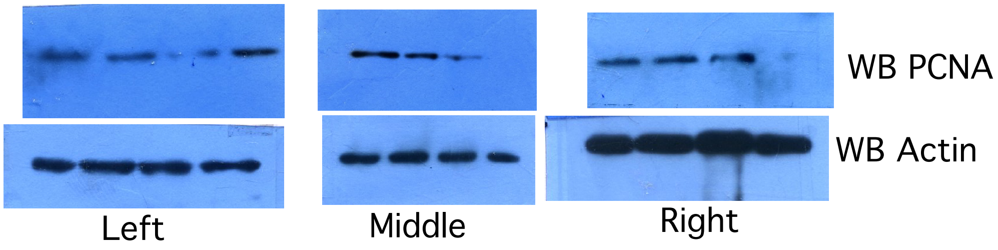

Supplement: Unedited blot and gel images [file jci-134-161941-s062.pdf]
